# COL2-dependent photoperiodic floral induction in *Nicotiana sylvestris* seems to be lost in the *N. sylvestris* × *N. tomentosiformis* hybrid *N. tabacum*

**DOI:** 10.3389/fpls.2023.1249879

**Published:** 2024-01-04

**Authors:** Florentin J. Schmidt, Lena Grundmann, Michael Lahme, Marvin Seidemann, Axel Schwarze, Sophie Lichtenauer, Richard M. Twyman, Dirk Prüfer, Gundula A. Noll

**Affiliations:** ^1^ Institute of Plant Biology and Biotechnology, University of Münster, Münster, Germany; ^2^ Fraunhofer Institute for Molecular Biology and Applied Ecology (IME), Münster, Germany; ^3^ TRM Ltd, Scarborough, United Kingdom

**Keywords:** flowering, photoperiod, *Nicotiana* spp., BBX-family, CONSTANS (CO), FLOWERING LOCUS T (FT)

## Abstract

**Introduction:**

Plants are sessile organisms that maximize reproductive success by adapting to their environment. One of the key steps in the reproductive phase of angiosperms is flower development, requiring the perception of multiple endogenous and exogenous signals integrated via a complex regulatory network. Key floral regulators, including the main transcription factor of the photoperiodic pathway (CONSTANS, CO) and the central floral pathway integrator (FLOWERING LOCUS T, FT), are known in many species.

**Methods and results:**

We identified several CO-like (COL) proteins in tobacco (*Nicotiana tabacum*). The NtCOL2a/b proteins in the day-neutral plant *N. tabacum* were most closely related to Arabidopsis CO. We characterized the diurnal expression profiles of corresponding genes in leaves under short-day (SD) and long-day (LD) conditions and confirmed their expression in phloem companion cells. Furthermore, we analyzed the orthologs of *NtCOL2a/b* in the maternal LD ancestor (*N. sylvestris*) and paternal, facultative SD ancestor (*N. tomentosiformis*) of *N. tabacum* and found that they were expressed in the same diurnal manner. *NtCOL2a/b* overexpression or knock-out using the CRISPR/Cas9 system did not support a substantial role for the CO homologs in the control of floral transition in *N. tabacum*. However, *NsCOL2* overexpression induced flowering in *N. sylvestris* under typically non-inductive SD conditions, correlating with the upregulation of the endogenous *NsFTd* gene.

**Discussion:**

Our results suggest that *NsFTd* is transcriptionally regulated by NsCOL2 and that this COL2-dependent photoperiodic floral induction seems to be lost in *N. tabacum*, providing insight into the diverse genetics of photoperiod-dependent flowering in different *Nicotiana* species.

## Introduction

The transition from vegetative to reproductive growth is essential for the reproductive success of flowering plants, and is also important for plant breeding and agriculture by influencing crop yields, biomass accumulation, seed production, and fruit ripening (Jung and Müller, 2009; Blümel et al., 2015; Schmidt et al., 2020). The molecular regulation of flowering has been investigated in many plant species, including important crops such as rice (*Oryza sativa*), potato (*Solanum tuberosum*), tomato (*Solanum lycopersicum*) (Tsuji et al., 2013; Abelenda et al., 2014; Lifschitz et al., 2014) and more recently common tobacco (*Nicotiana tabacum*), although in the latter case there is much still left to learn (Amaya et al., 1999; Smykal et al., 2007; Harig et al., 2012; Beinecke et al., 2018; Schmidt et al., 2020).

The onset of flowering is controlled by the integration of internal and external signals representing plant age, vernalization and day length, and their coordination with an inbuilt circadian clock (Imaizumi, 2010). Plant species can be assigned to three categories based on their ability to flower in response to long-day (LD) conditions (16-h photoperiod), short-day (SD) conditions (8-h photoperiod), or day-neutral conditions. The model species *Arabidopsis thaliana* (Arabidopsis) is a facultative LD plant, which means that it favors but is not restricted to LD flowering (Kobayashi and Weigel, 2007). The analysis of late-flowering Arabidopsis mutants revealed many genes that regulate flowering time, including *CONSTANS* (*CO*) (Koornneef et al., 1991). *CO* was subsequently found to encode the key transcription factor controlling photoperiod-dependent flowering, coordinating photoperiodic signals with the circadian clock (Putterill et al., 1995; Suárez-López et al., 2001). CO induces flowering by activating the floral pathway integrator gene *FLOWERING LOCUS T* (*FT*) in leaves (Samach et al., 2000; Suárez-López et al., 2001; Valverde et al., 2004). The regulation of *CO* expression at the transcriptional and post-translational levels ensures that photoperiod-dependent floral induction occurs only under LD conditions (Putterill et al., 1995; Samach et al., 2000; Suárez-López et al., 2001; Takada and Goto, 2003; An et al., 2004; Valverde et al., 2004). CO protein levels are low after dawn because *CO* transcription is repressed by cycling DOF factors (CDFs), but levels rise during the day when these negative regulators are degraded in the proteasome (Imaizumi et al., 2005; Sawa et al., 2007; Fornara et al., 2009). The accumulation of *CO* mRNA in the light allows the protein to be stabilized by light-mediated post-translational regulation (Valverde et al., 2004; Liu et al., 2008; Song et al., 2012). Afternoon light induces PHYTOCHROME A (PHYA), CRYPTOCHROME 1 (CRY1) and CRYPTOCHROME 2 (CRY2), which protect CO from degradation (Wang et al., 2001; Valverde et al., 2004; Liu et al., 2008; Zuo et al., 2011; Sheerin et al., 2015). Under LD conditions, FKF1 levels also peak during the day, helping to stabilize CO and ensuring the activation of *FT* (Song et al., 2012). However, under SD conditions the expression of *FKF1* peaks during the night, which prevents CO from reaching the threshold necessary to trigger *FT* transcription (Putterill et al., 1995; Valverde et al., 2004; Laubinger et al., 2006; Jang et al., 2008). This is because CO is degraded by COP1-SPA during the dark period (Suárez-López et al., 2001; Sawa et al., 2007; Fornara et al., 2009).

*CO* and *FT* homologs are present in diverse flowering plants (Crocco and Botto, 2013; Wickland and Hanzawa, 2015) and are assumed to form a highly conserved genetic module, but the nature of the regulatory interactions is species dependent (Ballerini and Kramer, 2011). Rice is a facultative SD plant that mainly flowers during short days but it can also flower under LD conditions, in both cases controlled by CO and FT. However, the nature of the regulatory hierarchy differs from that in Arabidopsis (Tsuji et al., 2011). For example, the rice CO homolog Heading date 1 (Hd1) is modulated by light but is also present in the dark and does not require light for post-translational stabilization (Ishikawa et al., 2011). Hd1 induces the *FT*-like genes *Heading date 3a* (*Hd3a*) and *RICE FLOWERING LOCUS T 1* (*RFT1*) in leaf phloem tissue under SD conditions (Yano et al., 2000; Izawa et al., 2002; Kojima et al., 2002), but other regulators (lacking counterparts in Arabidopsis) are also required (Takada and Goto, 2003; An et al., 2004; Doi et al., 2004; Tamaki et al., 2007; Komiya et al., 2008; Xue et al., 2008; Komiya et al., 2009). Hd3a is a major floral regulator under SD conditions (Kobayashi et al., 1999; Kojima et al., 2002; Komiya et al., 2008), but its expression is repressed by Hd1 under LD conditions (Izawa et al., 2002; Hayama et al., 2003; Ishikawa et al., 2011) and flowering is instead controlled by RFT1 (Komiya et al., 2009). In potato, the role of StCO in flowering is poorly understood and flowering may be controlled in response to other stimuli, such as irradiance (Navarro et al., 2011; González-Schain et al., 2012). In contrast to floral transition, tuber formation in potato *andigenum* genotypes requires short days and is dependent on the FT-like protein StSP6A (Rodríguez-Falcón et al., 2006). Like *StSP3D*, *StSP6A* is expressed in leaves, suggesting that long-distance transport is also important for tuber formation (Navarro et al., 2011; González-Schain et al., 2012). *StSP6A* is regulated by StCO in a photoperiod-dependent manner. StCO is stabilized during long days, allowing the repression of *StSP6A* and preventing tuberization (Navarro et al., 2011; González-Schain et al., 2012). Functional differences among the CO and FT homologs in Arabidopsis, rice and potato highlight the diversity of these protein families.

CONSTANS/CONSTANS-like (CO/COL) proteins are B-box zinc-finger transcription factors and are members of the large BBX protein family. The family has five subgroups (I–V) reflecting the distribution and structure of two conserved domains: an N-terminal B-box domain, which facilitates protein–protein interactions, and a C-terminal CCT domain, which is required for nuclear localization (Putterill et al., 1995; Yano et al., 2000; Robson et al., 2001; Griffiths et al., 2003; Valverde, 2011; Crocco and Botto, 2013). CO/COL proteins are assigned to subgroups I, II or III, all of which possess a CCT domain, whereas subgroups IV and V do not. There are two variants of the B-box domain (B1 and B2) reflecting the amino acid sequence and specificity of the zinc-binding residues. Subgroup I and II CO/COL proteins possess the B1 and B2 types, whereas subgroup III only possess the B1 domain (Robson et al., 2001; Crocco and Botto, 2013).

The model plant in this study (tobacco, *N. tabacum* cv. SR1) belongs to the genus *Nicotiana* (Solanaceae), and like ∼40% of the ∼75 known *Nicotiana* species has an allotetraploid genome arising from the interspecific hybridization of two diploid progenitors (Leitch et al., 2008; Sierro et al., 2014). Several phylogenetic studies have shown that day-neutral *N. tabacum* probably evolved from the maternal ancestor *N. sylvestris*, which shows obligate flowering under LD conditions, and the paternal ancestor *N. tomentosiformis*, which shows facultative flowering under SD conditions (Okamuro and Goldberg, 1985; Aoki and Ito, 2000; Kitamura et al., 2000; Murad et al., 2002; Clarkson et al., 2005; Skalická et al., 2005; Bombarely et al., 2012; Sierro et al., 2014). *N. tabacum* combines the two diploid ancestral genomes and has lost only 4–8% of the total DNA, thus remaining closely related to both species (Skalická et al., 2005; Sierro et al., 2013; Sierro et al., 2014). Tobacco homologs of several floral regulators have been identified, including the floral pathway integrator FT and the key floral transcription factor FD (Amaya et al., 1999; Smykal et al., 2007; Harig et al., 2012; Beinecke et al., 2018; Schmidt et al., 2020). The tobacco genome encodes multiple FT homologs, some of which (NtFT1–NtFT3) are floral inhibitors whereas others (NtFT4 and NtFT5) are floral activators (Harig et al., 2012; Beinecke et al., 2018; Wang et al., 2018). *NtFT1–NtFT4* are expressed predominantly under SD conditions whereas *NtFT5* is expressed under both SD and LD conditions, and unlike *FT* genes from other species none of the *NtFT* genes show a circadian expression profile (Harig et al., 2012; Beinecke et al., 2018). Silencing of the floral activator gene *NtFT5* by RNA interference significantly delayed flowering under LD conditions, whereas knocking out the *NtFT5* gene using CRISPR/Cas9 rendered the mutants completely unable to flower under LD conditions, indicating that NtFT5 is a major floral inducer during long days (Beinecke et al., 2018; Schmidt et al., 2020). Three functional FD homologs have also been identified in tobacco (NtFD1, NtFD3 and NtFD4) and they interact with tobacco FT proteins (Beinecke et al., 2018). Furthermore, *NtFT4* and *NtFT2* (encoding an activator and inhibitor, respectively) are expressed at similar levels under SD conditions, and the proteins show dose-dependent effects on flowering, suggesting they compete at the protein level for FD binding rather than using the mutual transcriptional regulation strategy described in sugar beet (*Beta vulgaris*) and potato (Pin et al., 2010; Abelenda et al., 2016; Beinecke et al., 2018). Indeed, NtFD1 preferentially interacts with the floral activator NtFT4 rather than the inhibitor NtFT2 (Beinecke et al., 2018). Therefore, although tobacco is a day-neutral plant, flowering is in part regulated by the photoperiod-dependent expression of different *FT* genes (Harig et al., 2012; Beinecke et al., 2018). However, it remains unclear how the expression of the different tobacco *FT* genes is regulated by upstream transcription factors such as the B-box protein CO. Recently, Song et al. (2022) identified 43 tobacco B-box encoding genes (*NtBBX*) representing all five subgroups. To determine the potential role of CO/BBX homologs as floral regulators in tobacco we investigated in detail the expression and activity of *NtBBX1* and *NtBBX2* in *N. tabacum* as well as their orthologs in the progenitors *N. sylvestris* and *N. tomentosiformis*. Our results provide insight into the diverse genetics of photoperiod-dependent flowering in different *Nicotiana* species.

## Materials and methods

### Plant material and growth conditions

We used four tobacco species in this study: *Nicotiana benthamiana* Domin, *Nicotiana tabacum* L. cv. SR1, *Nicotiana sylvestris* Speg. & Comes, and *Nicotiana tomentosiformis* Goodsp.. Wild-type tobacco plants were sown and cultivated in soil under LD conditions in the greenhouse (16-h photoperiod, artificial light switched on if natural light fell below 700 μmol m^-2^ s^-1^, 22–25°C under light, 19–25°C in the dark), or under SD conditions in phytochambers (8-h photoperiod, 200 μmol m^-2^ s^-1^, 25–27°C under light, 20°C in the dark). For *Agrobacterium*-mediated transformation, wild-type *N. tabacum* cv. SR1 and *N. sylvestris* plants were germinated and grown under sterile conditions (LD, 16-h photoperiod, 23°C, 100 μmol m^-2^ s^-1^) on MS medium (Murashige and Skoog, 1962). For the analysis of T_1_ transgenic *N. tabacum* lines carrying overexpression, empty vector control or promoter–reporter cassettes and transgenic *N. sylvestris* lines, seeds were germinated in a sterile environment under LD (*N. tabacum* only) or SD conditions on selective MS medium (25 mg/L hygromycin, 100 mg/L kanamycin or 3 mg/L phosphinothricin, as appropriate). Seedlings were then transferred to soil and cultivated in the greenhouse or in phytochambers as described above. In contrast, T_1_ plants and subsequent generations of the CRISPR/Cas9 knockout lines were directly sown and cultivated under LD or SD conditions in soil as described above. Plant material for the isolation of gene sequences, expression analysis, and immunodetection experiments was snap-frozen in liquid nitrogen immediately after harvest and stored at –80°C. Further information on plant material, growth conditions and the harvesting time points is set out in the **Supplementary Materials and Methods** (incl. **Supplementary Table S1**).

### Construct design and cloning

Constructs for overexpression, promoter–reporter analysis, immunodetection of fusion proteins, subcellular localization and CRISPR/Cas9 knockouts are described in the **Supplementary Materials and Methods** (incl. **Supplementary Tables S2**–**S8**).

### Extraction of nucleic acids and cDNA synthesis

Leaf material was ground in a mortar or MM400 bead mill (Retsch). Genomic DNA was extracted using the NucleoSpin Plant II kit (Macherey-Nagel), the protocol of Edwards et al. (1991) or, for analysis in a 96-well plate format, the Chemagic DNA Plant kit (PerkinElmer) and a PSU-2T Mini-Shaker (BioSan) for the resuspension of magnetic beads. Total RNA was isolated from leaf extracts using the innuPREP Plant RNA kit (Analytik Jena) and residual genomic DNA was digested using the TURBO DNA-free kit (Thermo Fisher Scientific). RNA quantity and quality were determined using a NanoPhotometer UV/Vis spectrophotometer (Implen) and by agarose gel electrophoresis. Complementary DNA (cDNA, final concentration 50 ng/mL) was synthesized from total RNA using Perfect Real Time PrimeScript RT Master Mix (Takara Bio Europe).

### Isolation of gene sequences

The *NtCOL2a/b*, *NtomCOL2* and *NsCOL2* gene sequences were isolated by PCR from *N. tabacum*, *N. tomentosiformis* and *N. sylvestris* genomic DNA, respectively. The isolated *NtCOL2a/b* sequence was amplified in two overlapping fragments and included about ∼2.5kb of the upstream promoter. The coding sequences of the genes were isolated by RT-PCR from *N. tabacum*, *N. tomentosiformis* or *N. sylvestris* RNA. Genomic and coding sequences were amplified using gene-specific primers (**Supplementary Table S2**). The resulting amplicons were either transferred to pCRII-TOPO using the TOPO TA Cloning kit (Thermo Fisher Scientific) or to pJET1.2/blunt using the CloneJET PCR Cloning kit (Thermo Fisher Scientific) for sequencing. For P*_NtCOL2a_* and P*_NtCOL2b_* sequence regions, amplicons were directly inserted by ligation into pBsGFP_ER_ (Noll et al., 2007) and pBsGUS (Schmidt et al., 2020) for sequencing, using appropriate restriction enzymes for digestion (for details, see **Supplementary Table S5**). The full-length *NtCOL2a/b* genomic sequences were assembled *in silico* using SeqManPro and SeqbuilderPro in Lasergene v15 (DNASTAR).

### *In silico* analysis of gene structures and protein sequences

The genomic structures of *NtCOL2a*, *NtCOL2b*, *NtomCOL2* and *NsCOL2* were determined by aligning the previously isolated genomic sequences with the corresponding coding sequences using SeqManPro and SeqBuilderPro in Lasergene v15. To determine sequence identities genomic, coding or protein sequences were aligned to determine sequence identities using EMBOSS Needle Pairwise Sequence Alignment (Madeira et al., 2019). The ClustalW module within MEGA-11 (Tamura et al., 2021) was used to align the CO(L)/BBX proteins of *Nicotiana tabacum* (Nt), *Oryza sativa* (Os), *Arabidopsis thaliana* (At), *Solanum lycopersicum* (Sl) and *Solanum tuberosum* (St) (for corresponding accession numbers, see **Supplementary Tables S9**–**11**), and the phylogenetic tree was constructed using the Neighbor-Joining (NJ) method with 1000 bootstrap-replications and generated with the iTOL online tool (https://itol.embl.de/). Domain analysis was carried out using a Clustal OMEGA multiple sequence alignment (Madeira et al., 2022) including CO homologs from *Oryza sativa* (Os), *Arabidopsis thaliana* (At), *Solanum lycopersicum* (Sl) and *Solanum tuberosum* (St) (for corresponding accession numbers, see **Supplementary Tables S9**–**S11**). InterProScan (Jones et al., 2014) was used to detect conserved protein domains. The identified domains were annotated manually according to Robson et al. (2001).

### *Agrobacterium*-mediated tobacco transformation

Transgenic lines of *N. tabacum* cv. SR1 and *N. sylvestris* Speg. & Comes were generated via the leaf disc method (Horsch et al., 1985) using the *A. tumefaciens* strain LBA4404 (Hoekema et al., 1983), in which the appropriate binary vectors were introduced by electroporation. Transgenic plants were selected on MS medium supplemented with 25 mg/L hygromycin, 100 mg/L kanamycin or 3 mg/L phosphinothricin, as appropriate. Independent transgenic plant lines were regenerated from callus tissue in sterile culture media and were tested for genomic transgene integration. To increase the probability of induced mutations, callus passage of the *NtCOL2* knockout lines was repeated by placing leaves of transgenic T_0_ plants on appropriate sterile MS medium and shoots were regenerated from callus tissue. After rooting, transgenic plants were transferred to the greenhouse and cultivated in soil under LD conditions as described above.

For localization studies, Venus-NtCOL2a and Venus-NtCOL2b fusion proteins were expressed in the leaves of 3–4-week-old *N. benthamiana* plants cultivated under LD conditions. For this purpose, *A. tumefaciens* strain GV3101 pMP90 (Koncz and Schell, 1986) was transformed with the appropriate binary vectors. Transient expression was then achieved by the co-infiltration of leaves with strains GV3101 pMP90 and C58C1, carrying the pCH32 helper plasmid and a pBin61 derivative expressing tomato bushy stunt virus RNA silencing suppressor p19 (Hamilton et al., 1996; Garabagi et al., 2012). After infiltration, plants were cultivated for 3 days with continuous illumination before proteins were localized by confocal laser scanning microscopy.

### Screening transgenic and genome edited plants

Regenerated T_0_ transgenic plants representing independent transformation events were identified by PCR using MangoTaq DNA polymerase (Bioline) with the primers listed in **Supplementary Table S2** and genomic DNA as the template. T_0_ and T_1_ generation knockout plants were screened to determine whether a genomic *cas9* gene was present, using *N. tabacum GLYCERALDEHYDE-3-PHOSPHATE DEHYDROGENASE* (*NtGAPDH*) as a template control. Genome editing of *NtCOL2a* and *NtCOL2b* was analyzed by PCR using MyTaq DNA polymerase (Bioline) and the primers listed in **Supplementary Table S2** for the amplification of exon I from genomic DNA. Starting with the T_0_ generation, plants were screened by direct sequencing of purified amplicons. Because the transgenic T_0_ plants were chimeras, selected individuals were analyzed in more detail by sequencing the amplicons following transfer to pCRII-TOPO (TOPO TA Cloning kit, Thermo Fisher Scientific).

### Quantitative real-time PCR (qPCR)

Gene expression in wild-type and transgenic plants was analyzed by qPCR using the CFX 96 Real-Time System in a C1000 Touch Thermal Cycler (Bio-Rad Laboratories) combined with KAPA SYBR FAST qPCR Master Mix (Merck) and gene-specific primers (**Supplementary Table S2**). The reactions contained 500 nM of each primer and 2.5 µL template cDNA (diluted 1:10, equivalent to ∼12.5 ng). After denaturation (95°C, 3 min), the qPCR program comprised 40 cycles of denaturation (95°C, 3 s) and annealing/extension for 30 s at primer-specific temperatures (**Supplementary Table S12**). Melt curve analysis (5 s, 58–95°C, ΔT = 0.5°C) was carried out to ensure amplicon specificity. Each sample was tested in technical triplicates for each gene, along with duplicate no-reverse-transcriptase (NRT) and no-template controls (NTC). Data were analyzed using CFX Manager v3.1 (Bio-Rad Laboratories). Quantification cycle (Cq) values of technical triplicates were averaged and used to determine the mean of each biological replicate. The target gene expression ratio was calculated as previously described (Livak and Schmittgen, 2001). The reference gene *ELONGATION FACTOR-1α* (*EF-1α*) was used for normalization (Schmidt and Delaney, 2010).

### SDS-PAGE and western blotting

NtCOL2a-3xc-myc and NtCOL2b-3xc-myc fusion proteins were detected by cultivating transgenic plants expressing P_Q35S_:*NtCOL2a-3xc-myc* or P_Q35S_:*NtCOL2b-3xc-myc* under LD conditions and grinding harvested leaf tissue in liquid nitrogen using a mortar. Proteins were extracted from 50 mg ground tissue per sample in 50 µL 5× SDS-PAGE buffer (60 mM Tris/HCl, 50% (v/v) glycerol, 10% (w/v) SDS, 500 mM DTT, 0.1% (w/v) bromphenol blue, pH ∼6.8) by vortexing (2 min) and boiling at 95°C (10 min). Mixtures were centrifuged to remove cell debris (10,000 × g, 2 min, room temperature) and the supernatant was fractionated by SDS-PAGE (Laemmli, 1970) on 10% (v/v) SDS polyacrylamide gels. The proteins were then transferred to nitrocellulose membranes (Towbin et al., 1979) and visualized by incubating for ∼1 min in 0.1% (w/v) Ponceau S, 5% (v/v) acetic acid, as a loading control (Romero-Calvo et al., 2010). After documentation, the stain was removed by soaking in phosphate-buffered saline (PBS; 140 mM NaCl, 2.7 mM KCl, 1.8 mM KH_2_PO_4_, 10 mM Na_2_HPO_4_, pH ∼7.2) containing 0.1% (v/v) Tween-20 (PBST). PBST containing 5% (w/v) skimmed milk powder was used for antibody dilution to prevent nonspecific binding. The 3xc-myc-tagged versions of NtCOL2a and NtCOL2b were detected using a mouse monoclonal anti-c-myc antibody (diluted 1:5000, Sigma-Aldrich #M4439). After further washing in PBST, the bound primary antibody was detected using a secondary goat anti-mouse IgG antibody coupled to horseradish peroxidase (Thermo Fisher Scientific #32430). After a final wash, the signal was revealed using the SuperSignal West Dura Extended Duration Substrate (Thermo Fisher Scientific) and G:BOX Chemi XX6 gel documentation system running GeneSys v1.5.2.0 (Syngene).

### Histochemical analysis of β-glucuronidase (GUS) activity

Transgenic plants expressing P*_NtCOL2a_*:*uidA* or P*_NtCOL2b_*:*uidA* were cultivated under LD conditions. Stem and leaf petiole sections and small leaf discs were infiltrated in a vacuum with GUS staining solution and incubated for up to 24 h at 37°C as previously described (Schmidt et al., 2020). Chlorophyll was extracted by incubating the samples in methanol (37°C for up to 3 h). Samples were stored in deionized water at 4°C before imaging with a MZ 16 F stereomicroscope (Leica Microsystems).

### Confocal laser scanning microscopy (CLSM)

Promoter activity in transgenic P*_NtCOL2a_*:*GFP_ER_* and P*_NtCOL2b_*:*GFP_ER_* plants was analyzed by CLSM using a Leica TCS SP5 X microscope (Leica Microsystems). Longitudinal sections of stem and leaf petioles were prepared from plants cultivated under LD conditions. Callose was stained with 0.1% (w/v) aniline blue in a 1:1 (v/v) mix of glycerol/deionized water for ~5 min to visualize phloem sieve tube plates. Sections were washed in the same solution without dye before microscopy. Fluorescence was measured at excitation and emission wavelengths of 488 and 500–600 nm (GFP_ER_), or 405 and 479–533 nm (aniline blue), respectively. Small discs punched from infiltrated *N. benthamiana* leaves were analyzed by CLSM to determine the subcellular localization of Venus-NtCOL2a and Venus-NtCOL2b fusion proteins after transient expression. Venus fluorescence signals were measured in abaxial epidermal cells at excitation and emission wavelengths of 514 and 525–600 nm, respectively.

### Accession numbers

The accession numbers of gene and protein sequences used in this study are listed in **Supplementary Tables S9**–**S11**.

## Results and discussion

### Identification of *NtCOL/BBX* genes and the spatial expression profile of *NtCOL2a/b*

**Figure 1:**
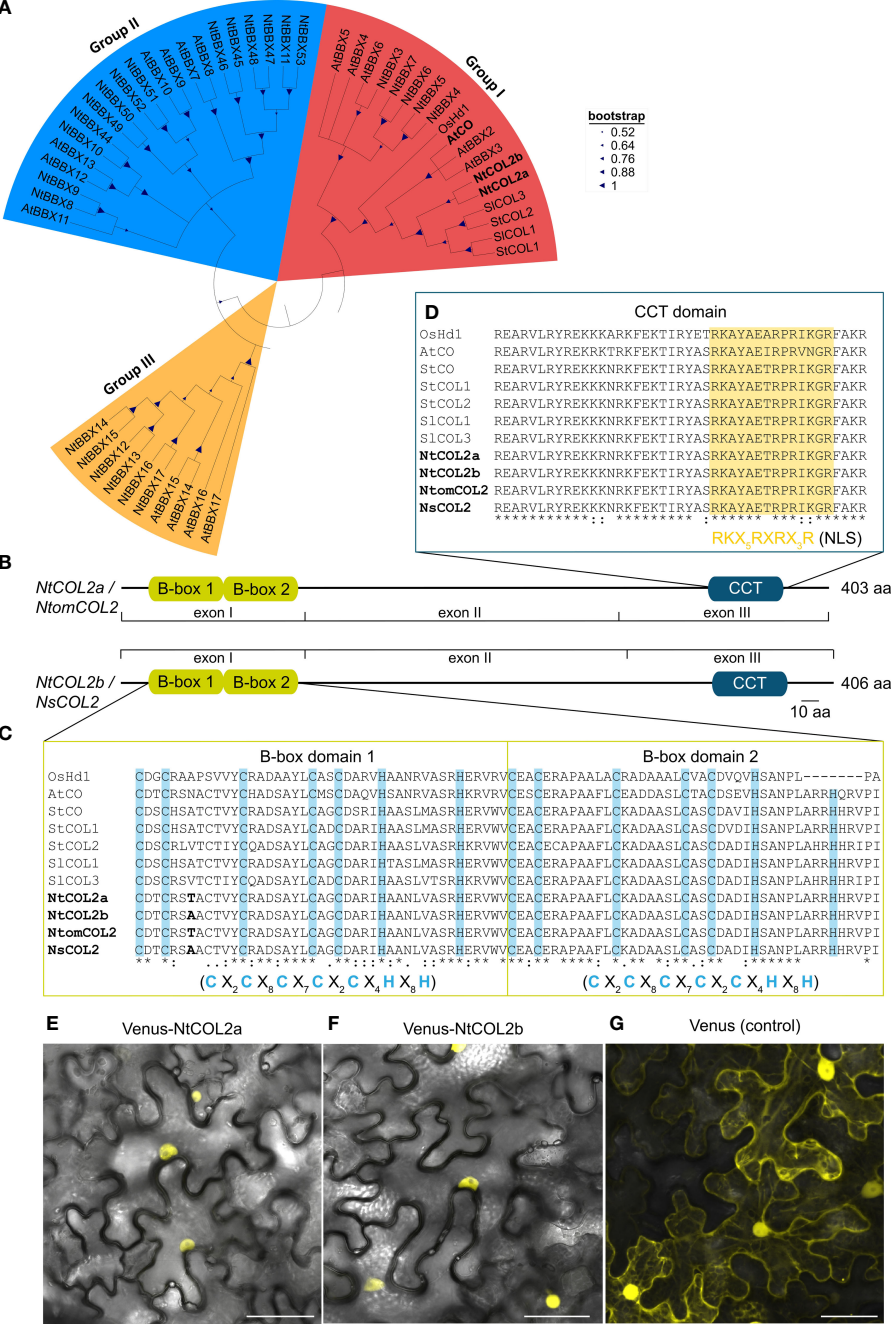
Tobacco COL/BBX homologs identified in *N. tabacum*, *N. tomentosiformis*, and *N. sylvestris*. **(A)** Part of the phylogenetic Neighbor-Joining tree of the identified tobacco COL/BBX protein sequences from **Supplementary Figure S1**. The tree is based on a ClustalW alignment within MEGA-11 (Tamura et al., 2021) and was constructed using the Neighbor-Joining (NJ) method with 1.000 bootstrap-replications, including CO(L)/BBX homologs from *Oryza sativa* (Os), *Arabidopsis thaliana* (At), *Solanum lycopersicum* (Sl) and *Solanum tuberosum* (St) (for corresponding accession numbers, see **Supplementary Tables S9** and **S11**). **(B)** Predicted domain structure of the tobacco COL2 proteins characterized in this study. The conserved domains were identified by comparison with protein sequence databases using the InterProScan online tool (Jones et al., 2014) and were annotated manually according to Robson et al. (2001). Conserved domains are shown as boxes, and residual parts as lines. **(C, D)** Part of the multiple sequence alignment from **Supplementary Figure S2** to show the CO-typical protein domains: B-box1, B-box2 **(C)**, and CCT **(D)**. The domain sequences in the tobacco homologs were compared with those found in the selected reference proteins. Characteristic cysteine and histidine residues conserved in the B-box domains are highlighted in blue (Robson et al., 2001), and the putative nuclear localization signal (NLS) in the CCT domain is highlighted in yellow (Crocco and Botto, 2013). Amino acid differences between the tobacco proteins are shown in bold. X in the consensus sequences of the indicated domains represents any amino acid. In the alignment, an asterisk indicates amino acids that are identical in all sequences, a colon indicates a conserved substitution and a period indicates a semi-conserved substitution. B-box, B-box zinc finger domain; CCT, CO/CO-like/TOC1 domain; CO, CONSTANS; COL, CONSTANS-LIKE; Hd1, Heading date 1; *At*, *A. thaliana*; *Ns*, *N. sylvestris*; *Nt*, *N. tabacum*; *Ntom*, *N. tomentosiformis*; *Os*, *O. sativa*; *Sl*, *S. lycopersicum*; *St*, *S. tuberosum*. **(E–G)** Subcellular localization of NtCOL2a and NtCOL2b in *N. benthamiana* leaf epidermis cells revealed by Venus fluorescence in abaxial epidermal cells expressing P_35S_:*Venus-NtCOL2a* **(E)**, P_35S_:*Venus-NtCOL2b* **(F)** and P_35S_:*Venus* **(G)** detected by confocal laser scanning microscopy. Venus-NtCOL2a **(E)** and Venus-NtCOL2b **(F)** fusion proteins are present in the nucleus. Venus localization **(G)** was used as a control. The representative cells were from *N. benthamiana* plants cultivated for 3 days under continuous light after transient protein expression. Scale bars = 50 µm. For single channel images see **Supplementary Figure S4**.

Initially, we searched for potential CO/BBX-related proteins in tobacco by using the 17 Arabidopsis CO/BBX proteins from subclades I–III (**Supplementary Table S9**) as BLAST queries against tobacco protein sequences in the National Center for Biotechnology Information (NCBI) non-redundant protein sequences (nr) database. This revealed numerous, mainly predicted tobacco sequences (for accession numbers see **Supplementary Table S9**) clustering with the different AtCO/AtBBX proteins (**Figure 1A**). We also mapped these tobacco proteins against the recently identified *NtBBX*/NtCOL sequences (Song et al., 2022; Zhao et al., 2022) and found that we could expand the list of NtBBX proteins (NtBBX44-53; **Supplementary Table S10**). Based on the phylogenetic tree containing all AtCO/AtBBX-family members, NtBBX44-53 were assigned to subgroup II (**Figure 1A**; **Supplementary Figure S1**). In terms of flowering control, AtCO is the first BBX protein characterized and NtBBX1 and NtBBX2 are the most closely related to AtCO (**Figure 1A**). Therefore, we focused on the characterization of these two proteins and refer to them as NtCOL2a (NP_001311813) and NtCOL2b (XP_016462705). Using NtCOL2a and NtCOL2b as BLAST queries, we specifically searched in *Nicotiana tomentosiformis* and *Nicotiana sylvestris* protein sequences in the NCBI nr database for ancestral orthologs and identified two closely related proteins, NtomCOL2 (XP_009630583) and NsCOL2 (XP_009765376). Pair-wise alignments of the protein sequences revealed that NtCOL2a evolved from the predicted NtomCOL2 sequence and NtCOL2b evolved from the predicted NsCOL2 sequence. NtCOL2a and NtCOL2b share 99.8% and 100% identity with the corresponding ancestral proteins, respectively, and are 94.6% identical to each other. Based on this, we identified and verified the genomic and coding sequences of *NtCOL2a, NtCOL2b, NsCOL2* and *NtomCOL2* and isolated the corresponding genomic DNA and cDNA sequences from cultivar SR1 and the progenitor species, respectively. Overall, genomic *NtCOL2a* and *NtCOL2b* shared 99.5% (*NtCOL2a* to *NtomCOL2*) and 99.8% (*NtCOL2b* to *NsCOL2*) identity with their ancestral genes, confirming the progenitor genomes are highly conserved in *N. tabacum*.

An alignment of genomic sequences with the corresponding isolated coding sequences revealed that each gene features three exons (I–III, **Supplementary Figure S3**). Further insight in the regulation of tobacco COL proteins was achieved by comparison of NtCOL2a/b amino acid sequences with CO(L)/BBX proteins from Arabidopsis, rice, potato and tomato (Yano et al., 2000; Robson et al., 2001; Ben-Naim et al., 2006; González-Schain et al., 2012; Abelenda et al., 2016; Zhao et al., 2022). A phylogenetic tree (**Figure 1A**) emphasized the evolutionary origin of NtCOL2a and NtCOL2b and the close relationship between the tobacco proteins and homologs from potato and tomato (solanaceous species) and Arabidopsis. NtCOL2a and NtCOL2b featured the domain structure typical for CO proteins, which includes two N-terminal B-box zinc finger domains and a C-terminal CCT domain (**Figures 1B–D**; **Supplementary Figure S2**), suggesting that the proteins are functional floral regulators (Putterill et al., 1995; Yano et al., 2000; Robson et al., 2001; Crocco and Botto, 2013). The screening of several protein databases verified the presence and location of the two B-box-type zinc finger domains (InterPro accession number IPR000315) and the CCT motif (IPR010402) in each of the four tobacco proteins (**Figure 1B**). The two B-boxes (designated B-box1 and B-box2) are directly adjacent to each other at the N-terminus, and the CCT motif is located in the characteristic C-terminal position (Robson et al., 2001; Griffiths et al., 2003; Crocco and Botto, 2013). The predicted B-boxes and CCT domain were near identical in all four tobacco proteins, with only one amino acid differing between NtCOL2a and NtCOL2b, and between NtomCOL2 and NsCOL2, at the seventh position in B-box 1 (**Figures 1C, D**). Moreover, the tobacco domains were highly similar to those in the reference proteins, apart from distantly-related OsHd1 (**Figures 1C, D**). Both B-boxes featured a CO-typical consensus structure consisting of five cysteine and two histidine residues separated by a defined number of amino acids (Robson et al., 2001; Griffiths et al., 2003; Crocco and Botto, 2013). The CCT motif included a nuclear localization signal (NLS) also found in other, diverse CO homologs (Crocco and Botto, 2013). Indeed, N-terminal fusions of NtCOL2a and NtCOL2b with Venus (Nagai et al., 2002) expressed in *N. benthamiana* leaf epidermal cells were detected in the nucleus (**Figures 1E, F**; **Supplementary Figure S4**). The unfused Venus protein (control) was localized in the cytoplasm and nucleus (**Figure 1G**; **Supplementary Figure S4**).

**Figure 2:**
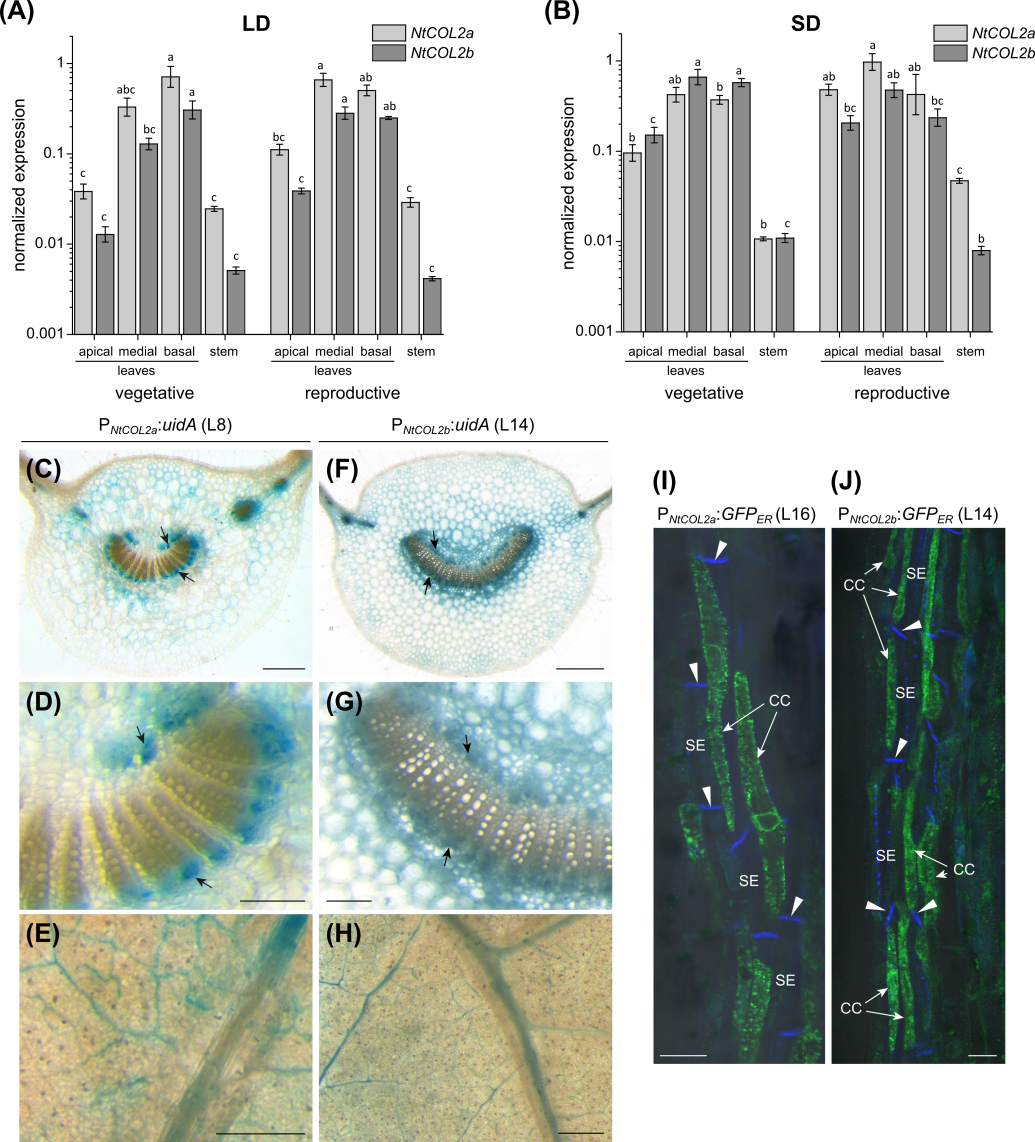
Spatial expression profile of *NtCOL2a* and *NtCOL2b* in *N. tabacum* cv. SR1. **(A, B)** Expression in the leaves and stems of vegetative and reproductive wild-type plants analyzed by quantitative real-time PCR (qPCR) at dawn (0 h) under long-day (LD) **(A)** and short-day (SD) **(B)** conditions. *NtCOL2a* and *NtCOL2b* expression was normalized to the reference gene *NtEF-1α*. Data are means of three biological replicates ± standard errors (SEM) based on log-transformed data. Statistically significant differences for each gene (shown using different lower case letters) were determined by one-way ANOVA and Tukey’s *post hoc* test (*P <*0.05). **(C–H)** GUS activity in the medial leaves of transgenic P*_NtCOL2a_*:*uidA* **(C–E)** and P*_NtCOL2b_*:*uidA* plants **(F–H)**. Stained phloem tissue is indicated with arrows. The panels show cross sections **(C, D, F, G)** of the petiole and small discs punched from the leaf lamina **(E, H)**. **(I, J)** GFP_ER_ fluorescence in transgenic P*_NtCOL2a_*:*GFP_ER_* **(I)** and P*_NtCOL2b_*:*GFP_ER_* **(J)** plants detected by confocal laser scanning microscopy (CLSM). Fluorescence is abundant in the phloem companion cells (CCs) adjacent to sieve elements (SEs) in the petioles (representative longitudinal petiole sections are shown). The callose-containing sieve plates (indicated with arrowheads) are stained with aniline blue. **(C–J)** Representative sections were prepared from T_0_ plants cultivated after regeneration from callus tissue under LD conditions ∼6 weeks (P*_NtCOL2a_*:*uidA*, L8), ∼3 weeks (P*_NtCOL2b_*:*uidA*, L14), ∼5 weeks (P*_NtCOL2a_*:*GFP_ER_*, L16), and ∼2 weeks (P*_NtCOL2b_*:*GFP_ER_*, L14) after transfer from sterile culture to the greenhouse. Scale bars **(C, E, F, H)** = 1 mm; **(D, G)** = 250 µm; **(I, J)** = 2 µm.

Next, we characterized the spatial expression of *NtCOL2a/b* by qPCR and promoter–reporter analysis. *NtCOL2a* and *NtCOL2b* expression was monitored by qPCR at two developmental stages under LD and SD conditions, focusing on the apical, medial, and basal leaves, as well as the stem. The tissues were harvested at dawn from vegetative and reproductive plants, the latter with visible floral buds. *NtCOL2a* and *NtCOL2b* were expressed at similar levels under LD (**Figure 2A**) and SD (**Figure 2B**) conditions, and during vegetative and reproductive growth, but levels were highest in the mature medial and basal leaves and lowest in the stem. Previous studies have shown that CO regulates the expression of *FT* in the phloem companion cells of leaves (Takada and Goto, 2003; An et al., 2004; Wigge et al., 2005; Chen et al., 2018). We therefore used promoter–reporter analysis to define the cell-specific spatial expression profile by fusing the ~2.5 kb P*_NtCOL2a_* or P*_NtCOL2b_* promoter sequences to either *uidA* encoding GUS or to *GFP_ER_*. Stable transformation of *N. tabacum* cv. SR1 plants resulted in several independent T_0_ transformants per construct, which were cultivated and analyzed under LD conditions. Promoter activity was investigated in the medial leaves due to their high expression levels. GUS activity was analyzed in the petiole (**Figures 2C, D, F, G**) and lamina (**Figures 2E, H**), revealing P*_NtCOL2a_* and P*_NtCOL2b_* acted predominantly in the vascular bundles, specifically in the phloem, as reported for the *AtCO* promoter (An et al., 2004). This expression pattern was observed in at least three transgenic T_0_ plants per construct, but P*_NtCOL2a_*:*uidA* L8 (**Figures 2C–E**) and P*_NtCOL2b_*:*uidA* L14 (**Figures 2F–H**) are shown as representative examples. Additional but less intense staining of parenchymal tissue regions was detected in petiole cross-sections (e.g., **Figures 2C, F**), indicating that *NtCOL2a* and *NtCOL2b* expression may not be restricted to the vascular bundles. Promoter activity in the P*_NtCOL2a_*:*GFP_ER_* and P*_NtCOL2b_*:*GFP_ER_* lines was determined by CLSM using longitudinal petiole sections, confirming gene expression in the phloem. In at least five T_0_ plants per construct, GFP_ER_ was detected in phloem companion cells adjacent to the sieve elements, which were visualized by callose-specific aniline blue staining of the sieve plates, but representative sections are shown for P*_NtCOL2a_*:*GFP_ER_* L16 (**Figure 2I**) and P*_NtCOL2b_*:*GFP_ER_* L14 (**Figure 2J**). These experiments confirmed *NtCOL2a* and *NtCOL2b* expression at the site of *FT* transcription, as also observed for *NtFT3* in *N. tabacum* (Harig et al., 2012).

### *Nicotiana COL2* genes are expressed in a diurnal pattern

**Figure 3:**
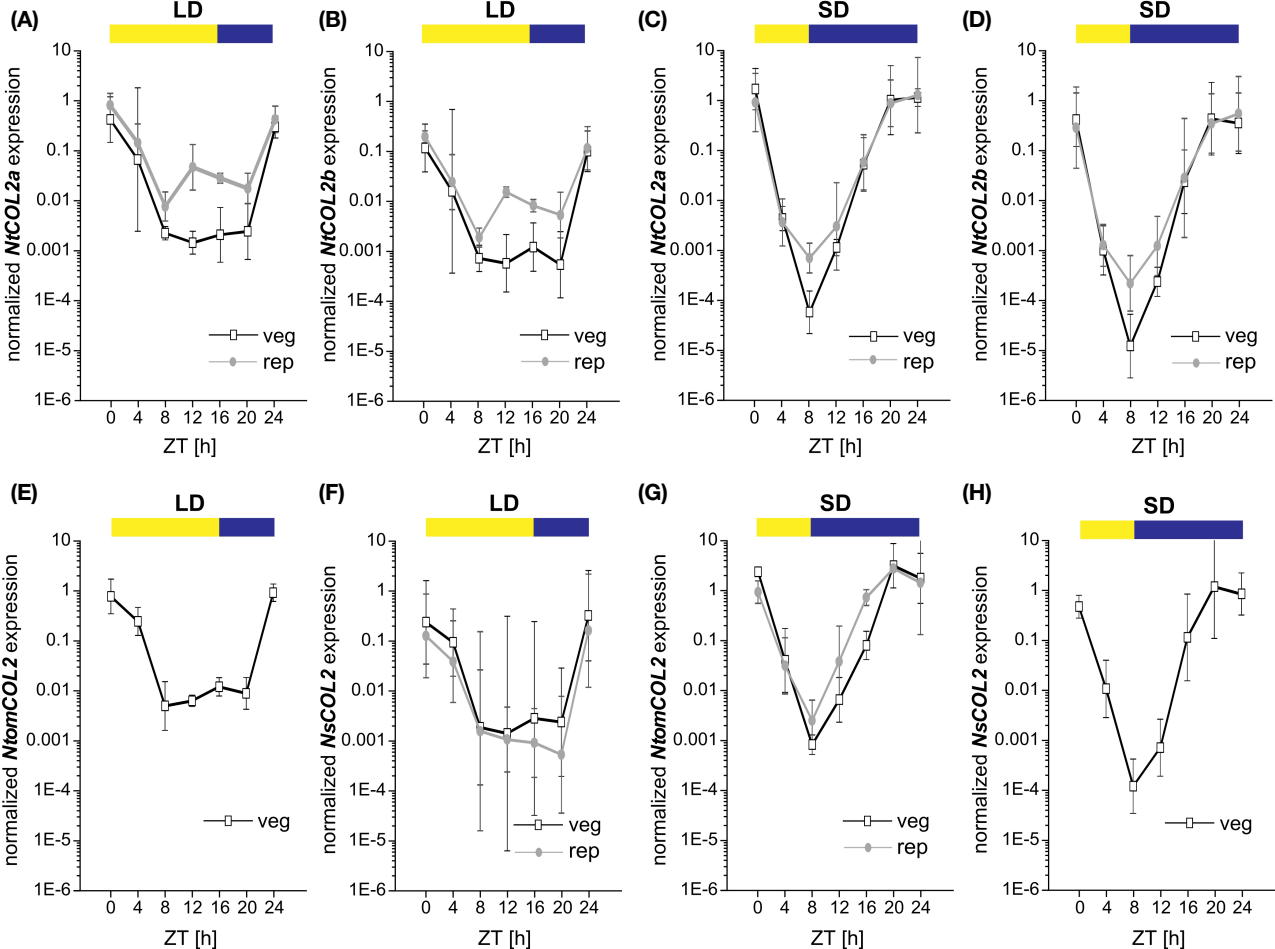
Diurnal expression of *NtCOL2a* and *NtCOL2b* in *N. tabacum* cv. SR1, *NtomCOL2* in *N. tomentosiformis* and *NsCO2* in *N. sylvestris*. **(A–H)** Expression of *NtCOL2a* **(A, C)**, *NtCOL2b* **(B, D)**, *NtomCOL2* **(E, G)**, and *NsCOL2* **(F, H)** in medial leaves of vegetative and reproductive wild-type plants analyzed by quantitative real-time PCR (qPCR) under long-day (LD) **(A, B, E, F)** and short-day (SD) **(C, D, G, H)** conditions. Samples were harvested at the indicated times with 4-h intervals during the day starting at dawn (zeitgeber time (ZT) = 0 h). Yellow and blue boxes indicate light and dark periods, respectively. *NtCOL2a* and *NtCOL2b* expression was normalized to the reference gene *NtEF-1α, NtomCOL2* expression to *NtomEF-1α*, and *NsCOL2* expression to *NsEF-1α*. Data are means of three biological replicates of medial leaves pooled from three plants for each replicate ± 95% confidence intervals of the biological replicates based on log-transformed data.

*CO* expression is precisely controlled by the internal circadian clock and photoperiod. *CO* transcript abundance in diverse plant species thus follows a diurnal rhythm, and the CO protein is stabilized at the post-translational level only when high expression levels coincide with the light period (Song et al., 2014). To examine the temporal expression profile of *NtCOL2a* and *NtCOL2b*, qPCR was carried out at 4-h intervals during one day under LD and SD conditions. Again, we selected medial leaves of vegetative and reproductive individuals due to the high general expression in this tissue. *NtCOL2a* and *NtCOL2b* expression followed similar oscillating patterns (**Figures 3A–D**), with a different daily course under LD (**Figures 3A**, **B**) and SD (**Figures 3C**, **D**) conditions. However, *NtCOL2a* (**Figures 3A**, **C**) and *NtCOL2b* (**Figures 3B**, **D**) expression were comparable in vegetative and reproductive plants grown under the same conditions. The general profile comprised a peak of expression around dawn followed by a steep drop during the light period and a renewed increase in the dark. Under LD conditions (**Figures 3A, B**), *NtCOL2a* and *NtCOL2b* expression peaked sharply at the beginning of the light period. Despite a slight decrease, relatively high expression levels were still detected 4 h after dawn, indicating the coincidence of high transcript abundance with the morning light. After reaching the expression minimum 8 h after dawn, a transient and less intense second peak was detected at the end of the light period (12–16 h after dawn), which was more obvious during reproductive growth. Nevertheless, the expression of both genes remained relatively low until the middle of the dark period (20 h after dawn), suggesting that transcript levels increased only late in the night. In contrast, the highest levels of *NtCOL2a* and *NtCOL2b* mRNA under SD conditions were detected between 20 h and dawn, indicating that the mRNA accumulated mostly in the dark (**Figures 3C, D**). This peak shift towards the late night went along with an earlier and more intense depletion of transcript levels in the morning light, which was already detected 4 h after dawn. The lowest expression levels were observed 8 h after dawn, corresponding with the end of the light period. The transcript abundance then increased continuously during the dark, beginning to peak late at night (20 h after dawn).

The day-neutral flowering behavior of *N. tabacum* emerged as a result of the tetraploidization of *N. tomentosiformis* (facultative SD flowering) and *N. sylvestris*, which strictly flowers under LD conditions (Aoki and Ito, 2000; Murad et al., 2002). To determine whether the diurnal expression patterns of *NtCOL2a* and *NtCOL2b* differ from those of their progenitor genes, we measured the abundance of *NtomCOL2* (**Figures 3E, G**) and *NsCOL2* (**Figures 3F, H**) mRNA by qPCR. Given the flowering behavior of *N. tomentosiformis* and *N. sylvestris*, *NtomCOL2* transcript levels under LD conditions (**Figure 3E**) and *NsCOL2* transcript levels under SD conditions (**Figure 3H**) were only tested in vegetative plants. Under both conditions, *NtomCOL2* and *NsCOL2* expression profiles generally resembled those of the *N. tabacum* genes, showing nearly the same peaks and troughs during the course of the day. However, one minor variation we occasionally observed was a rapid depletion of *NsCOL2* mRNA 20 h after dawn in the reproductive *N. sylvestris* plants under LD conditions. In summary, the diurnal profile of the *Nicotiana COL* genes, with expression levels peaking around dawn, is similar to that reported for the potato *CONSTANS-LIKE 1* (*StCOL1*) gene (Abelenda et al., 2016).

### Overexpression of *NtCOL2* genes has a negligible effect on flowering time in *N. tabacum*

**Figure 4:**
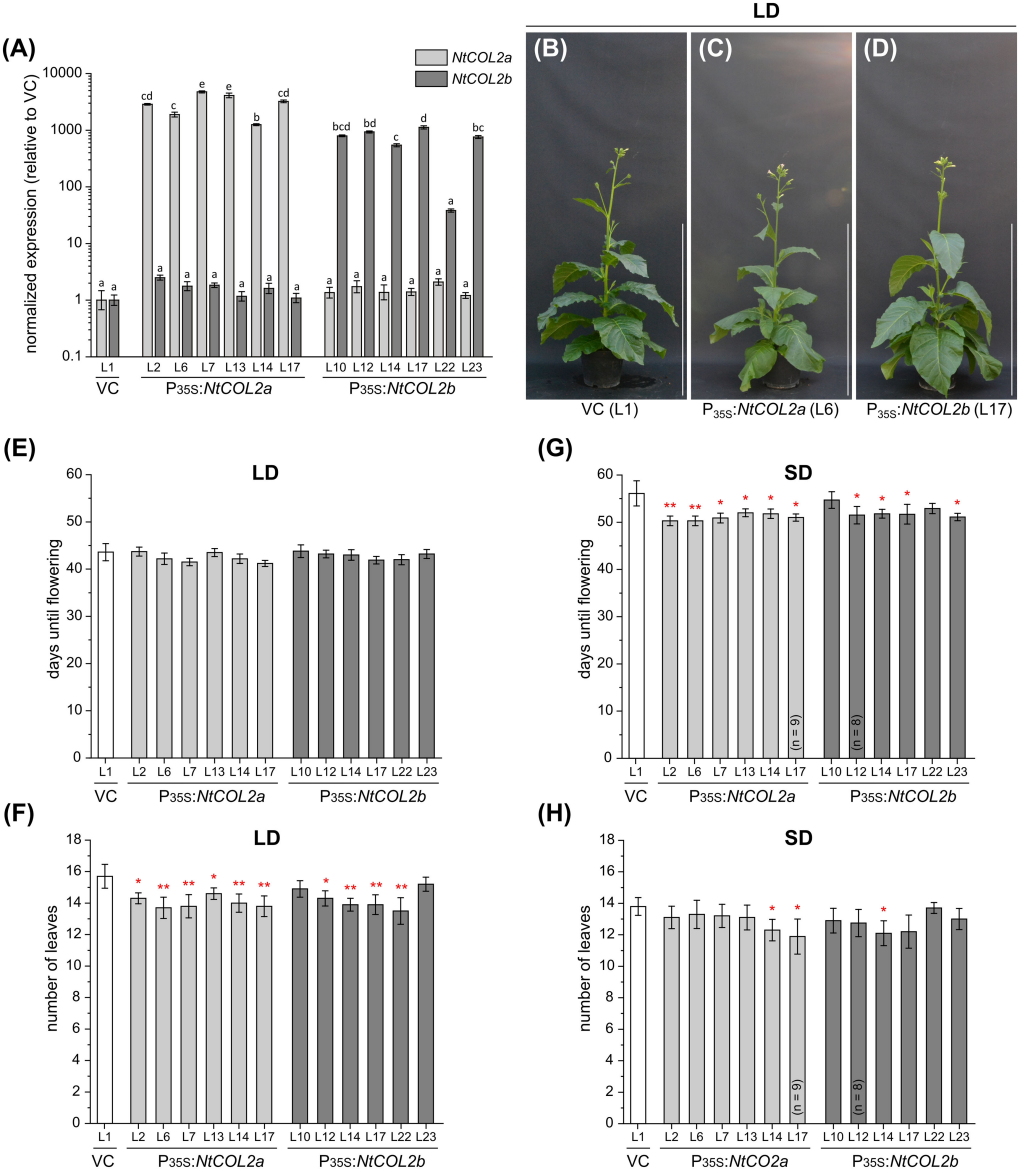
Constitutive overexpression of *NtCOL2a* and *NtCOL2b* in *N. tabacum* cv. SR1. **(A–H)** Analysis of transgenic P_35S_:*NtCOL2a* and P_35S_:*NtCOL2b* T_1_ individuals (six independent lines each) compared with the VC_pBin19 Hyg_ vector control (VC) line (L1) under long-day (LD) **(B–F)** and short-day (SD) **(A, G, H)** conditions. **(A)** Expression of *NtCOL2a* and *NtCOL2b* in seedlings detected by quantitative real-time PCR (qPCR). The seedlings were cultivated under sterile SD conditions and harvested 4 h after dawn. Expression of *NtCOL2a* and *NtCOL2b* was normalized to the reference gene *NtEF-1α.* Expression levels in the VC seedlings served as a reference and were set to 1. Data are means of three technical replicates of at least three pooled seedlings ± standard deviations of the technical replicates based on log-transformed data. Statistically significant differences for each gene between each overexpression line and the VC (shown using different lower case letters) were determined by one-way ANOVA and Tukey’s *post hoc* test (*P <*0.01). **(B–D)** Phenotypes at an early flowering stage when the first individuals had already opened their first flowers. The overexpression lines and VC are each represented by one individual grown under LD conditions ∼5.5 weeks after transfer from sterile culture to the greenhouse. Scale bars = 1 m. **(E, G)** Days until flowering, defined as the period between transfer from sterile culture to the greenhouse and the day the first flower opened. **(F, H)** Number of leaves on the main shoot determined at an early flowering stage. **(E–H)** Data are means (n = 10 unless stated otherwise) ± 95% confidence intervals. Normal distribution of the data was determined by applying the Kolmogorov-Smirnov test. Under each cultivation condition, the statistical significance of the difference between each overexpression line and the VC plants was assessed by applying Welch’s *t*-test. *P*-values were adjusted by applying Holm-Bonferroni correction (**P < 0.01; *P < 0.05).

To determine the effects of *NtCOL2a/b* overexpression, we generated transgenic P_35S_:*NtCOL2a* and P_35S_:*NtCOL2b N. tabacum* lines, cultivated T_1_ individuals representing six independent lines each under LD and SD conditions, and compared them with empty vector control plants (VC_pBin19 Hyg_ L1) carrying pBin19 Hyg T-DNA (Bevan, 1984, modified by Dr. Lena Grundmann, Münster, Germany), hereafter abbreviated to VC (**Figure 4**). All overexpression lines accumulated higher levels of the corresponding transcript than the VC (**Figure 4A**). *NtCOL2a* mRNA levels were at least ~1252-fold higher (L14) and up to ~4752-fold (L7) higher in the P_35S_:*NtCOL2a* lines, and *NtCOL2b* mRNA levels were generally at least ~543-fold higher (L14) and up to ~1122-fold higher (L17) in the P_35S_:*NtCOL2b* lines, except L22 (38-fold). The accumulation of *NtCOL2b* mRNA in the P_35S_:*NtCOL2a* lines and vice versa was comparable to the VC plants, indicating no cross-regulation between *NtCOL2a/b*. The overexpression of neither *NtCOL2a* nor *NtCOL2b* obviously affected the flowering behavior of the T_1_ plants (representative examples under LD conditions shown in **Figures 4B–D**). Under LD conditions, all *NtCOL2a* and *NtCOL2b* overexpression lines flowered at the same average time as the VC plants (**Figure 4E**), although most of the lines tended to produce ~1–2 fewer leaves on the main shoot before flowering (**Figure 4F**), which is statistically significant (suggesting the higher *NtCOL2a* or *NtCOL2b* transcript levels have a slight negative impact on plant development) but not biologically relevant. The P_35S_:*NtCOL2a* and P_35S_:*NtCOL2b* plants cultivated under SD conditions tended to flower up to 5 days earlier than the VC (**Figure 4G**) with the exception of P_35S_:*NtCOL2b* L10 and L22. This may indicate a slight acceleration of floral transition when high *NtCOL2a/b* transcript levels are abundant under SD conditions. However, the slight variations between the lines did not correlate with the transcript levels of the overexpressed gene. Moreover, the slightly shorter vegetative growth phase was not reflected in the number of leaves on the main shoot, which was on average comparable to the VC (**Figure 4H**). In conclusion, overexpression of *NtCOL2a* and *NtCOL2b* had no obvious effect on flowering behavior. In line with these observations, AtCO overexpression in tobacco also has no impact on flowering time (Ben-Naim et al., 2006).

**Figure 5:**
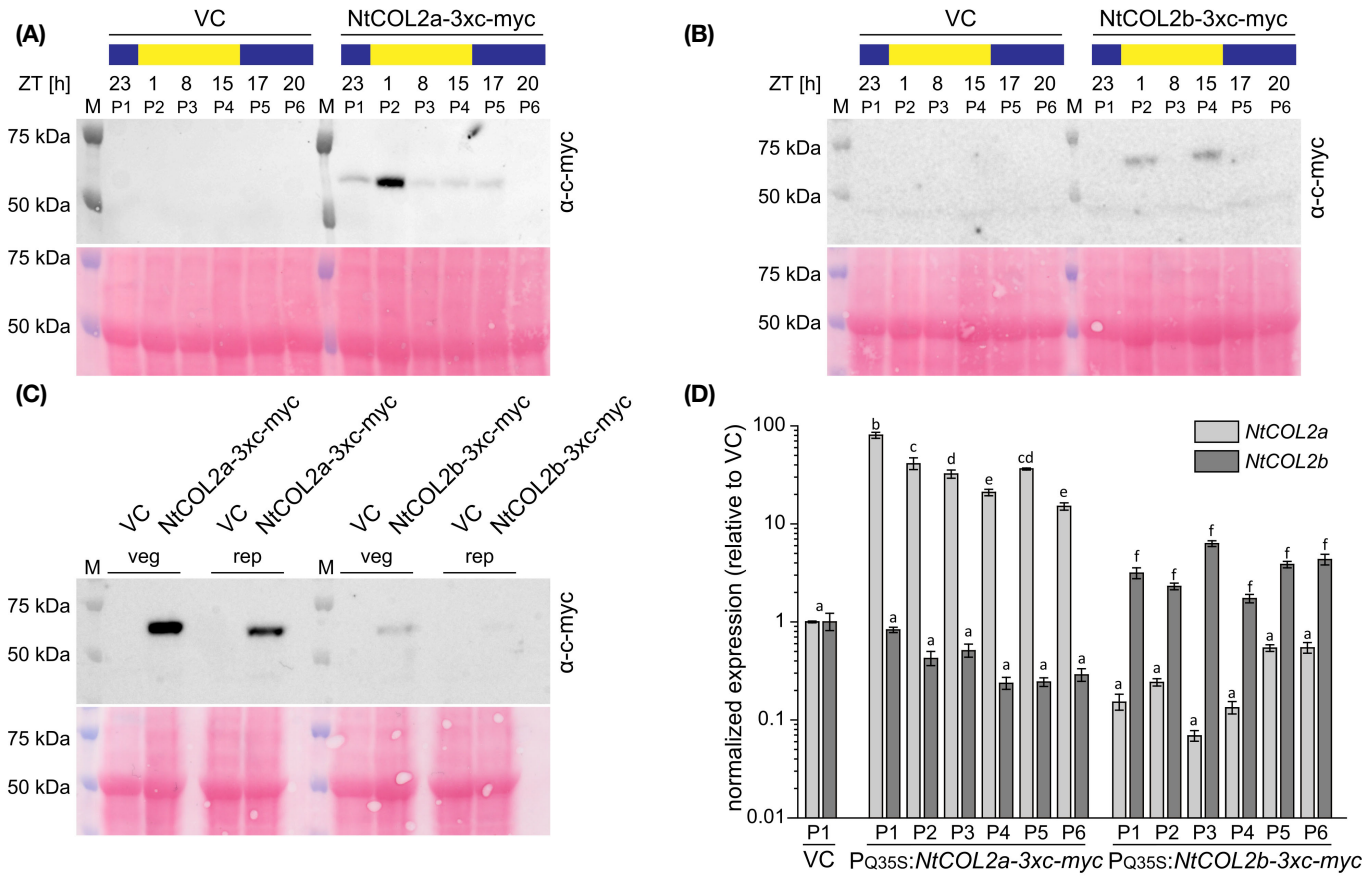
Abundance of NtCOL2a and NtCOL2b proteins in *N. tabacum* cv. SR1 plants under long-day (LD) conditions. **(A–C)** Immunodetection of NtCOL2a-3xc-myc and NtCOL2b-3xc-myc expressed in the leaves of transgenic P_Q35S_:*NtCOL2a-3xc-myc* (L5) and P_Q35S_:*NtCOL2b-3xc-myc* (L11) T_1_ plants, with the VC_pBin19_ vector control (VC) line (L1) as a negative control. Diurnal protein abundance was investigated in vegetative plants at the six indicated zeitgeber times (ZTs) during the day **(A, B)** and compared in vegetative (veg) and reproductive (rep) individuals 1 h after dawn **(C)**. Each sample consisted of leaves from three pooled individuals (pools designated P1–P6). For immunodetection, equal amounts of protein extract were separated by SDS-PAGE. After transfer to a nitrocellulose membrane, NtCOL2a-3xc-myc and NtCOL2b-3xc-myc were detected using a mouse primary anti-c-myc antibody (α-c-myc) and a goat anti-mouse IgG secondary antibody conjugated to horse radish peroxidase (HRP). Reversible Ponceau S staining of the membranes (shown below the immunodetection images) confirmed equal sample loading. **(D)** *NtCOL2a* and *NtCOL2b* expression in the leaves of transgenic P_Q35S_:*NtCOL2a-3xc-myc* (L5) and P_Q35S_:*NtCOL2b-3xc-myc* (L11) T_1_ plants investigated by quantitative real-time PCR (qPCR). Plants were analyzed in the same pools (P1–P6) used for immunodetection. The leaf tissue was harvested from the vegetative plants 4 h after dawn, ∼3 weeks after transfer from sterile culture to the greenhouse. Expression of *NtCOL2a* and *NtCOL2b* was normalized to the reference gene *NtEF-1α.* Expression levels in VC plants (P1) served as a reference and were set to 1. Data are means of three technical replicates of leaves pooled from three plants ± standard deviations of the technical replicates based on log-transformed data. Statistically significant differences between each overexpression line and the VC (shown using different lower case letters) were determined by one-way ANOVA and Tukey’s *post hoc* test (*P <*0.05).

Next, we checked the abundance of NtCOL2 proteins during the day. In Arabidopsis, light mediates the post-translational stabilization of CO, causing diurnal variations in protein abundance (Valverde et al., 2004). To determine whether NtCOL2a and/or NtCOL2b are stabilized in *N. tabacum*, we measured the protein levels by expressing *NtCOL2a-3xc-myc* and *NtCOL2b-3xc-myc* under the control of the quadruple constitutive CaMV 35S promoter (P_Q35S_). Given that NtCO-specific antibodies are not available for immunodetection, we added a C-terminal (3xc-myc) epitope tag (Evan et al., 1985). We recovered several independent *N. tabacum* T_0_ transformants per construct expressing the transgene cassette. Two of the corresponding T_1_ lines (P_Q35S_:*NtCOL2a-3xc-myc* L5 and P_Q35S_:*NtCOL2b-3xc-myc* L11) were chosen for the analysis of protein abundance under LD conditions compared to the pBin19 (Bevan, 1984) VC line (L1). Total protein extracts from young medial leaves were analyzed by SDS-PAGE and western blotting at six different time points during the day (**Figures 5A, B**), each sample representing a plant pool (P1–P6) that consisted of individuals from the same line. NtCOL2a-3xc-myc (**Figure 5A**) and NtCOL2b-3xc-myc (**Figure 5B**) levels varied during the day and the band size (50–75 kDa) appeared slightly larger than calculated *in silico* (~49.5 kDa). Nevertheless, the absence of any corresponding signal in the VC samples confirmed the specificity of these bands. NtCOL2a-3xc-myc was present at all time points, except 20 h after dawn in the middle of the night. Remarkably, the protein strongly accumulated in the morning light (1 h after dawn) and remained at relatively low levels for the rest of the day, despite the constitutive expression of the transgene. In contrast, NtCOL2b-3xc-myc was only present at low levels, and was detected in the early morning (1 h after dawn) and the evening (15 h after dawn), suggesting differences in the post-translational stability of these proteins. The presence of both proteins in the light indicated that the post-translational stabilization of NtCOL2a and NtCOL2b in *N. tabacum* might be dependent on light and/or the circadian clock. This was supported by the accumulation of NtCOL2a-3xc-myc 1 h after dawn, the time when *NtCOL2* transcript levels peak under LD conditions (**Figures 3A, B**). At this time, both fusion proteins were also detected when the plants formed visible floral buds (**Figure 5C**), indicating that protein stabilization also occurs during reproductive growth. However, more proteins tended to accumulate in the vegetative plants (**Figure 5C**).

To determine whether the varying protein levels reflected post-translational modifications or post-transcriptional regulation of the P_Q35S_:*NtCOL2-3xc-myc* transcripts, we analyzed *NtCOL2a* and *NtCOL2b* expression by qPCR in the leaves of the vegetative plant pools 4 h after dawn. Compared to the VC, all pools of the overexpression lines accumulated higher levels of the overexpressed transcript (**Figure 5D**). *NtCOL2a* levels varied between ~15.0-fold (P6) and ~80.2-fold (P1) higher in the P_Q35S_:*NtCOL2a-3xc-myc* (L5) plants, whereas *NtCOL2b* levels were elevated by ~1.7-fold (P4) to ~6.3-fold (P3) in the P_Q35S_:*NtCOL2b-3xc-myc* (L11) plants. These slight variations in expression level did not correlate with the observed diurnal oscillations of the NtCOL2a-3xc-myc or NtCOL2b-3xc-myc proteins. For example, the highest level of *NtCOL2* mRNA was detected in P1 of P_Q35S_:*NtCOL2a-3xc-myc* (L5), where the protein abundance was low. Nevertheless, the low *NtCOL2b* mRNA levels (**Figure 5D**) in all T_1_ plants of P_Q35S_:*NtCOL2b-3xc-myc* (L11) might explain the general low abundance of the NtCOL2b-3x-myc protein (**Figures 5B, C**).

### *NtCOL2* knockout has only a marginal effect on flowering time

**Figure 6:**
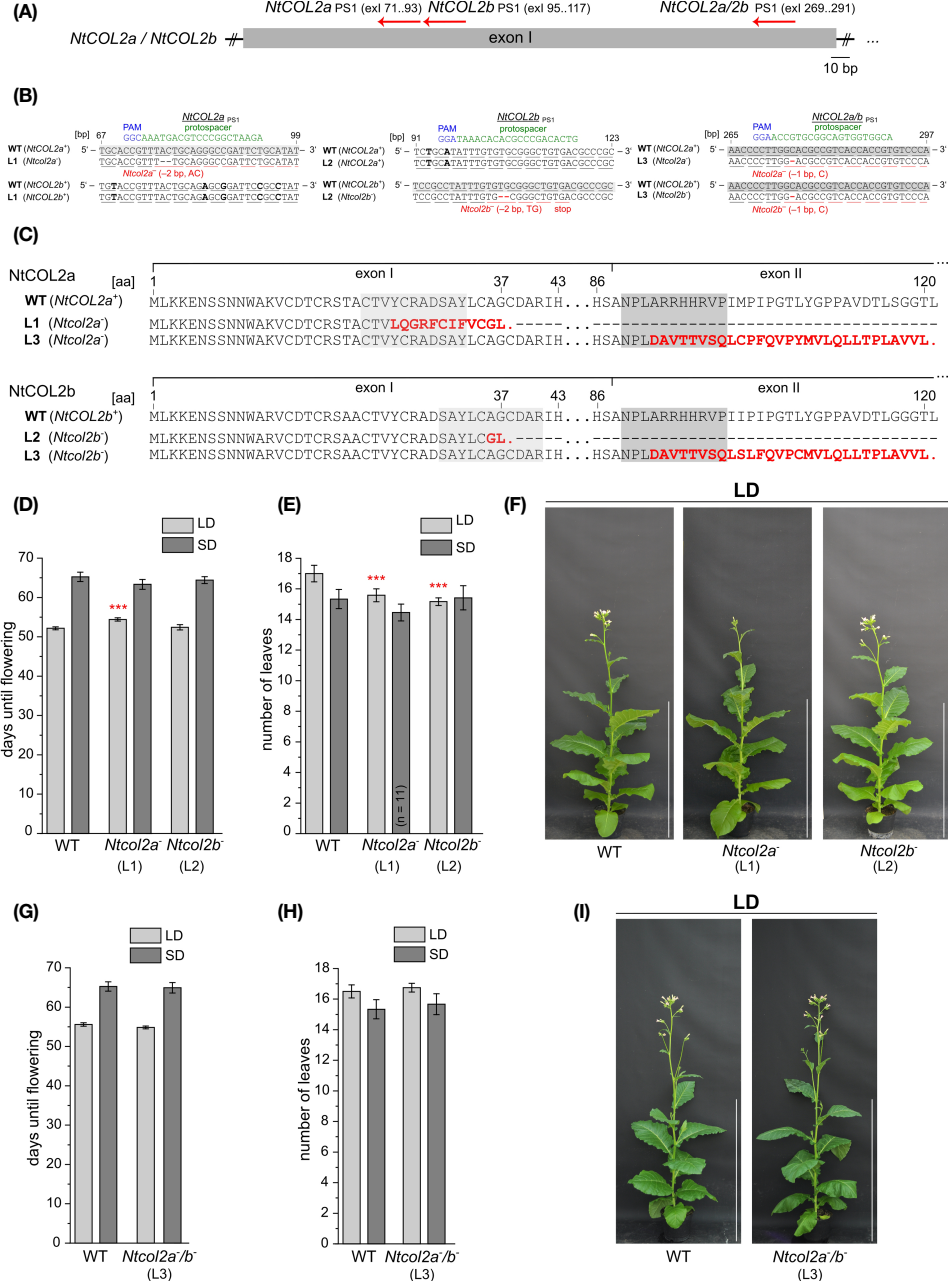
CRISPR/Cas9 genome editing of *NtCOL2a* and *NtCOL2b* in *N. tabacum* cv. SR1. **(A)** Genomic locations of the derived protospacers (PSs) and the corresponding protospacer adjacent motifs (PAMs) on the antisense DNA strand (red arrows) in exon I of *NtCOL2a* and *NtCOL2b*. Three PSs were derived, one specifically targeting *NtCOL2a* (*NtCOL2a*_PS1_), one specifically targeting *NtCOL2b* (*NtCOL2b*_PS1_), and the last targeting both genes (*NtCOL2a/b*_PS1_). Exons are shown as boxes, introns as lines. **(B)** Genotyping of *NtCOL2a* and *NtCOL2b* by direct PCR sequencing. Analysis shown for the representative nullizygous T_1_ individuals *Ntcol2a^–^* L1, *Ntcol2b^–^* L2, and *Ntcol2a^–^/b^–^* L3, each carrying two identical mutated alleles of the appropriate target gene(s) as indicated. Partial alignments of *NtCOL2a* and *NtCOL2b* in the T_1_ plants with the native sequences *NtCOL2a^+^* and *NtCOL2b^+^* in a wild-type (WT) control. The PSs are highlighted in green, the PAMs in blue, and sequence differences between *NtCOL2a* and *NtCOL2b* in bold. Numbers refer to the locations of PSs and PAMs in the *NtCOL2a* and *NtCOL2b* coding regions (in base pairs, bp). Underlines indicate triplets that correspond to gray highlighted amino acids (aa) in NtCOL2a or NtCOL2b **(C)**. **(C)** Alignment of wild-type NtCOL2a and NtCOL2b with their truncated protein versions encoded by the identified *Ntcol2a^–^* and *Ntcol2b^–^* alleles. Numbers refer to the amino acid positions in the wild-type proteins. Deletions and amino acid substitutions are highlighted in red letters. Red stops indicate premature termination. **(D–I)** Detailed phenotyping of nullizygous *Ntcol2a^–^*, *Ntcol2b^–^* single knockout and *Ntcol2a^–^/b^–^* double knockout T_2_ plants. For characterization, T_2_ individuals of one self-fertilized nullizygous T_1_ plant of *Ntcol2a^–^* L1, *Ntcol2b^–^* L2 and *Ntcol2a^–^/b^–^* L3 were cultivated alongside wild-type (WT) controls under long-day (LD) and short-day (SD) conditions (n = 12 per condition). The nullizygous genotypes were confirmed by genotyping (direct PCR sequencing) of representative T_2_ plants (n = 3 per condition). Phenotypic analysis of the nullizygous *Ntcol2a^–^* and *Ntcol2b^–^* single knockout plants **(D–F)**, and the *Ntcol2a^–^/b^–^* plants **(G–I)**. **(D, G)** Days until flowering, defined as the period between seed sowing and the day the first flower opened. **(E, H)** Number of leaves on the main shoot determined at an early flowering stage. **(F, I)** Phenotypes at an early flowering stage, when the plants had already opened their first flowers, ∼7.5 weeks **(F)** and ∼8 weeks **(I)** after seed sowing (WASS). Nullizygous knockout plants and the WT are each represented by one individual grown under LD conditions. Scale bars = 1 m. **(D, E, G, H)** Data are means (n = 12 unless stated otherwise) ± 95% confidence intervals. Normal distribution of the data was determined by applying the Kolmogorov-Smirnov test. The statistical significance of the difference between each knockout line and the WT control plants was assessed by applying Welch’s *t*-test. *P*-values were adjusted by applying Holm-Bonferroni correction (***P < 0.001).

Given that the lack of an obvious phenotype caused by the constitutive overexpression of *NtCOL2a* or *NtCOL2b*, we generated single and double knockout mutants in *N. tabacum* using the CRISPR/Cas9 system (Fauser et al., 2014). Gene specific protospacers were designed *in silico* using CCTop (Stemmer et al., 2015) and used as part of the sgRNA to generate the corresponding binary vector constructs for stable plant transformation. Proximal frameshift mutations were induced by designing protospacers (*NtCOL2a*_PS1_ and *NtCOL2b*_PS1_) within the antisense strand of exon I (**Figure 6A**). For the single knockouts, we used one protospacer per gene, and off-target effects were avoided by ensuring that the corresponding sites in the other *NtCOL2* gene featured at least three mismatches in the protospacer region or lacked a protospacer adjacent motif (PAM) of the “NGG” or “NRG” type recognized by SpCas9 endonuclease (Hsu et al., 2013). For the double knockout (*NtCOL2a/b*_PS1_), we targeted a region that was identical in both genes. Other potential off-target sites were identified by computational screening of the *N. tabacum* genome (cv. Basma Xanthi) as a reference (Sierro et al., 2014). This revealed that all three protospacers showed at least four mismatches when compared to any other putative exonic off-target region.

Several independent T_0_ transformants were generated and screened for induced mutations. T_1_ lines from three selected T_0_ transformants (*NtCOL2a*_PS1_ L1, *NtCOL2b*_PS1_ L2 and *NtCOL2a/b*_PS1_ L3) carrying mutated allelic variants (hereafter *Ntcol2a^–^* and *Ntcol2b^–^*) were cultivated under LD conditions, and three *cas9*-free plants per line were randomly chosen for further analysis. We screened for induced mutations by amplicon sequencing of *NtCOL2a* and *NtCOL2b* genomic DNA. All T_1_ individuals solely carried mutated alleles of the target gene(s), which were identical in all plants of the same line (**Figure 6B**) and had already been found in the T_0_ generation. In the single knockout *Ntcol2a^–^* and *Ntcol2b^–^* plants, the corresponding off-target region in the other *NtCOL2* gene was not mutated, confirming the specificity of the protospacers. The *Ntcol2a^–^* T_1_ plants were homozygous *NtCOL2a* knockouts with deletions of the dinucleotide AC (**Figure 6B**). Similarly, the *Ntcol2b^–^* T_1_ plants were homozygous *NtCOL2b* knockouts with deletions of the dinucleotide TG (**Figure 6B**). In the double knockout *Ntcol2a^–^/b^–^*, the *NtCOL2a* and *NtCOL2b* genes carried the same deletion of a single cytidine residue (**Figure 6B**). All mutations resulted in highly truncated NtCOL2a and/or NtCOL2b proteins (**Figure 6C**). The –2 bp deletions in the nullizygous *Ntcol2a^–^* and *Ntcol2b^–^* single knockout T_1_ plants generated proteins of 37 amino acids, containing only the N-terminus with the first 23 amino acids of B-box 1. Due to the position of the protospacer, the nullizygous *Ntcol2a^–^/b^–^* double knockout T_1_ plants generated proteins of 120 amino acids, which were nevertheless highly likely nonfunctional because they lack the CCT domain including the NLS and only contain the N-terminal B-box domains.

To determine the phenotype of the *NtCOL2* knockouts, we examined the flowering behavior of the mutants in the T_2_ generation. The offspring of one self-fertilized T_1_ plant of *Ntcol2a^–^* L1, *Ntcol2b^–^* L2 and *Ntcol2a^–^/b^–^* L3 were cultivated under LD and SD conditions and compared to wild-type controls. Representative genotyping of at least six individuals per line confirmed the nullizygous genotypes of the T_2_ offspring. Under SD conditions, the loss of NtCOL2 appeared to have no significant impact on flowering behavior. The single and double knockout individuals flowered on average at the same time as wild-type controls (**Figures 6D, G**) and there was no difference in the number of leaves (**Figures 6E, H**). Under LD conditions, the single *Ntcol2a^–^* knockout plants flowered slightly later than controls (~2 days) but this was not observed for the nullizygous *Ntcol2b^–^* plants (**Figure 6D**, representative plants in **Figure 6F**). However, both *Ntcol2a^–^* and *Ntcol2b^–^* plants tended to produce ~2 fewer leaves on the main shoot compared to controls (**Figure 6E**), indicating a mild effect on development that was statistically significant but not biologically relevant. Interestingly, this trend was not confirmed in the double knockouts, which produced a wild-type phenotype (representative plants in **Figure 6I**) with a similar flowering time (**Figure 6G**) and a comparable number of leaves (**Figure 6H**). We also measured by qPCR the expression level of four tobacco *FT* genes in medial leaves (harvested 4 h after dawn) in nullizygous *Ntcol2a^–^*, *Ntcol2b^–^* and *Ntcol2a^–^/b^–^* plants grown under LD and SD conditions. However, we found no differences in expression levels compared to wild-type controls (**Supplementary Figure S5**). These experiments suggested that the loss of NtCOL2 activity has only a marginal effect on flowering behavior, and only under LD conditions.

COL proteins have little or no influence on floral transition in some species, including homologs in day-neutral flowering tomato and potato varieties (Ben-Naim et al., 2006; González-Schain et al., 2012). As shown for NtCOL2a and NtCOL2b, the overexpression of the tomato *COL* genes *SlCOL1* and *SlCOL3* had no obvious impact on the flowering of transgenic tomato plants, and they are unlikely to be key floral regulators (Ben-Naim et al., 2006). In potato *andigenum* genotypes, the CO homologs StCO and StCOL1 also have only a weak influence on flowering (González-Schain et al., 2012; Abelenda et al., 2016). The close phylogenetic relationship between NtCOL2a, NtCOL2b and these tomato and potato proteins (**Figure 1A**) strengthens the hypothesis that the two tobacco homologs have little or no activity as floral regulators. Furthermore, phylogenetic analysis of CO in Arabidopsis and related Brassicaceae species revealed that CO and its homologs evolved by gene duplication from one common ancestral gene. However, the function of CO as a key regulator of photoperiodic flowering seems to have emerged after this duplication event. The regulation of photoperiod-dependent processes by CO homologs in other plant families may reflect the convergent evolution of gene function (Simon et al., 2015), which appears not to be the case for the *NtCOL2* genes.

### Overexpression of *NsCOL2* in *N. sylvestris* induces flowering under SD conditions

The published allotetraploid *N. tabacum* genome contains five *FT*-like genes (*NtFT1–NtFT5*), which can be associated with their ancestral genes in the two diploid progenitor species *N. sylvestris* (strict LD plant) and *N. tomentosiformis* (facultative SD plant) (Aoki and Ito, 2000; Murad et al., 2002; Harig et al., 2012; Sierro et al., 2014; Beinecke et al., 2018). *Nicotiana* FTs act antagonistically to regulate flowering, and for simplicity hereafter we identify the floral repressors (rep) and activators (act) using superscript notation. NtFT1^rep^, NtFT2^rep^, NtFT3^rep^ and NtFT4^act^ are primarily SD-specific floral regulators, whereas NtFT5^act^ induces flowering under SD and agriculturally-relevant LD conditions. Interestingly, NtFT5^act^ originates from *N. tomentosiformis* (NtomFTγ^act^). Therefore, LD flowering in *N. tabacum* seems to be based largely on the facultative SD *N. tomentosiformis* flowering pathway rather than the strict LD-dependent flowering pathway in *N. sylvestris*. Furthermore, under SD conditions, *N. tomentosiformis* flowering is strongly promoted by NtomFTb^act^ (homologous to NtFT4^act^). Under inductive LD conditions, *N. sylvestris* expresses NsFTc^act^ (homologous to NtFT6^act^, carrying a premature stop codon in SR1) and NsFTd^act^ (homologous to NtFT7^act^, not present in SR1), thereby promoting flowering, whereas flowering under SD conditions might be suppressed by the strong expression of NsFTa^rep^ (homologous to NtFT2^rep^) (Beinecke et al., 2018). In this study, the overexpression of *NtCOL2a* and *NtCOL2b* did not substantially affect the flowering time in *N. tabacum* and, at least under LD conditions, 3xc-myc-tagged NtCOL2a and NtCOL2b proteins were present in transgenic plants and followed a diurnal pattern (i.e., largely following the diurnal expression profile of the endogenous *NtCOL2a* and *NtCOL2b* genes despite constitutive expression under the control of the 35S promoter). *NtCOL2a* and/or *NtCOL2b* knockout only marginally affected the flowering time under LD conditions in *N. tabacum*. These results suggest, that, despite their observed abundance, the NtCOL2 proteins appear to have little or no function in terms of day-neutral floral transition and do not act as floral key regulators in *N. tabacum*.

**Figure 7:**
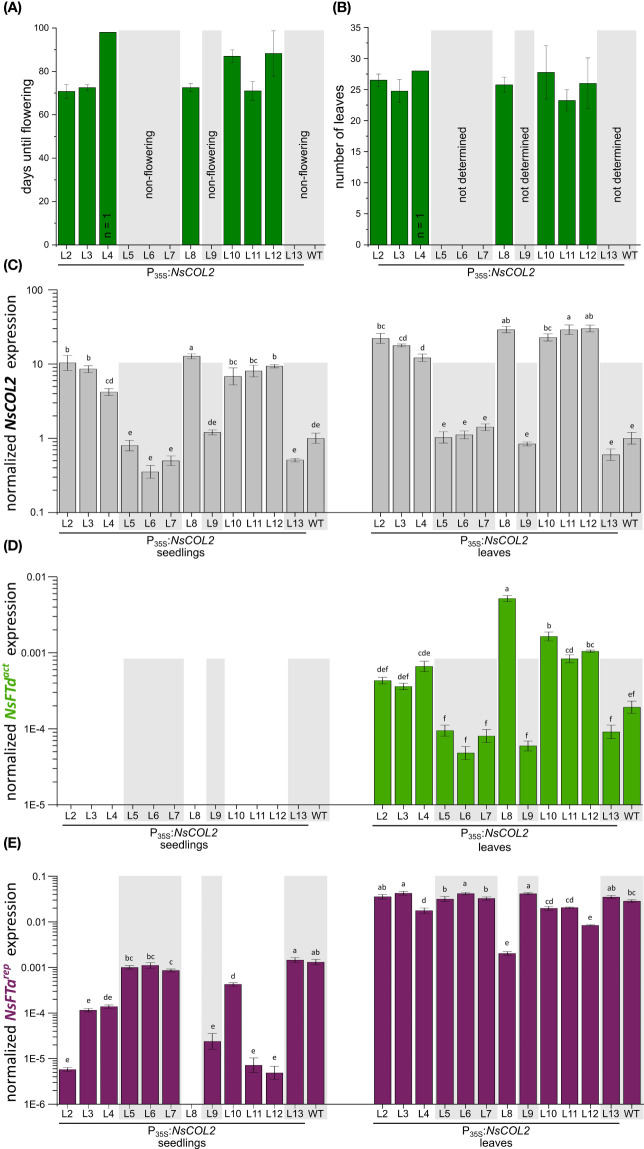
Constitutive overexpression of *NsCOL2* in *N. sylvestris* and analysis of its impact on flowering under typically non-inductive SD conditions. Analysis of transgenic P_35S_:*NsCOL2* T_1_ individuals in comparison to *N. sylvestris* wild-type plants (WT) under SD conditions. **(A, B)** Days until flowering **(A)** and number of leaves of seven transgenic lines overexpressing *NsCOL2* at flowering **(B)**, while five other lines and *N. sylvestris* wild-type plants (WT) did not flower (highlighted with gray boxes). Data are means (n = 4, unless stated otherwise) ± standard deviations. **(C–E)** Relative expressions levels of *NsCOL2* **(C)**, *NsFTd^act^* **(D)** and *NsFTa^rep^* **(E)** in seedlings and leaves. Transgenic seedlings were germinated on selective medium and at least three pooled seedlings were harvested at the 4–6-leaf stage. Four plants per line, including *N. sylvestris* WT control plants, were further cultivated under SD conditions. Leaf material was harvested from individual plants when the seven transgenic lines started flowering. Non-flowering transgenic lines and *N. sylvestris* WT plants are highlighted with gray boxes. *NsCOL2*, *NsFTd ^act^* and *NsFTa ^rep^* expression was normalized to the reference gene *NsEF-1α*. Data are means of three technical replicates ± standard errors (SEM) based on log-transformed data. Statistically significant differences between each overexpression line and the WT control (shown using different lower case letters) were determined by one-way ANOVA and Tukey’s *post hoc* test (*P <*0.05).

Although we introduced the P_35S_:*NtCOL2a* construct into *N. tomentosiformis*, we were unable to produce transgenic lines. However, we introduced the P_35S_:*NtCOL2b* construct (encoding NtCOL2b 100% identical to NsCOL2) into the strict LD plant *N. sylvestris* and recovered 12 independent transgenic T_0_ lines. In the T_1_ generation, seeds of all lines germinated under SD conditions on selective medium (with *N. sylvestris* wild-type seeds as controls on non-selective medium) and four seedlings per line were transferred to a phytochamber for phenotyping under SD conditions 6 weeks after germination. Seedling material was also harvested for qPCR analysis. *N. sylvestris* wild-type plants and all plants of five transgenic lines (L5, L6, L7, L9 and L13) grew solely in a vegetative manner and remained at the rosette growth stage, reflecting normal growth under these typically non-inductive SD conditions. Interestingly, the remaining seven lines (L2, L3, one of four L4 plants, L6, L8, L10, L11 and L12) started bolting and flowering 10–13 weeks after transfer to the phytochamber, resulting in only 24–27 leaves (**Figures 7A, B**).

Flowering in *Nicotiana* species strongly depends on antagonistically acting FTs, so we determined the levels of *NsCOL2*, the floral repressors *NsFTa^rep^* and *NsFTb^rep^*, and the floral activators *NsFTc^act^* and *NsFTd^act^* in seedlings and leaves of mature transgenic plants in the T_1_ generation (**Figures 7C–E**; **Supplementary Figure S5**). This revealed 12–30-fold higher *NsCOL2* expression levels in the leaves of flowering transgenic lines compared to the *N. sylvestris* wild-type, while non-flowering transgenic lines showed comparable or even lower *NsCOL2* expression levels (**Figure 7C**). *NsCOL2* overexpression thus correlated with the induction of flowering in the transgenic lines. *NsCOL2* levels in seedlings were lower than in mature plants but showed the same trend, with higher levels in lines that started flowering after cultivation under SD conditions. Furthermore, although we could not detect *NsFTd^act^* expression in seedlings from any transgenic or wild-type lines, *NsFTd^act^* expression increased in the leaves of flowering plants, despite being typically very low in wild-type *N. sylvestris* plants under these condition (**Figure 7D**; Beinecke et al., 2018). Non-flowering transgenic plants from the other lines accumulated even less *NsFTd^act^* mRNA than wild-type *N. sylvestris* plants. We also observed lower expression (near qPCR detection limit) of the floral repressor *NsFTa^rep^* in seedlings from lines that started flowering, ranging from 3-fold (L10) and 11-fold (L3) to more than 260-fold (L12) lower than wild-type levels (**Figure 7E**). In the seedlings of non-flowering lines, *NsFTa^rep^* remained at wild-type expression levels as expected (with the exception of non-flowering L9, where expression was ~50-fold lower than wild-type plants). In the leaves of mature plants, *NsFTa^rep^* expression levels increased for all plants compared to the seedling stage, with no correlation between *NsFTa^rep^* expression levels and a flowering phenotype (except L8, where expression is more than 10-fold lower than in other lines or WT; **Figure 7E**). We also checked the expression of *NsFTb^rep^* and *NsFTc^act^* (**Supplementary Figures S6A, B**) but found no correlation with the flowering or non-flowering phenotype.

In wild-type *N. sylvestris* plants, the floral repressor *NsFTa^rep^* is strongly expressed during vegetative growth under SD conditions, probably repressing flowering, although *NsFTc^act^* is also expressed (Beinecke et al., 2018). The overexpression of *NsCOL2* in transgenic *N. sylvestris* plants under SD conditions potentially suppressed *NsFTa^rep^* expression in transgenic seedlings, enabling flowering after a certain period of vegetative growth. In contrast, *NsFTa^rep^* expression levels in the leaves of mature transgenic lines were comparable to wild-type levels regardless of the flowering/non-flowering phenotype (**Figure 7E**). However, although *NsFTd^act^* expression was near the qPCR detection level in wild-type *N. sylvestris* and non-flowering transgenic lines (Beinecke et al., 2018; **Figure 7D**), it increased in flowering *NsCOL2* overexpression lines, suggesting that *NsFTd^act^* expression is activated by *NsCOL2* overexpression, which overcomes the relatively high expression of *NsFTa^rep^* detected in all lines. In flowering transgenic lines, the floral activator/repressor ratio is higher than in non-flowering lines or wild-type *N. sylvestris* plants and is thus shifted toward floral induction. We have already proposed the importance of the activator/repressor ratio in the transition to flowering in *N. tabacum*, where the floral promoter *NtFT4^act^* is expressed at a lower level than *NtFT1^rep^* and *NtFT2^rep^* under SD conditions, but the fold increase in abundance during development is much higher than the two floral repressors (Harig et al., 2012). Likewise, others have discussed local ratios of FT-like and TFL1-like proteins that control the balance between determinate and indeterminate growth in tomato (Shalit et al., 2009; McGarry and Ayre, 2012).

NsCOL2 may have a dual role, acting as a repressor of *NsFTa^rep^* while inducing the expression of *NsFTd^act^*. A dual role has also been described for OsHd1: although this protein acts as a floral repressor under LD conditions by repressing the expression of *Hd3a* (FT homolog and floral activator), it induces the expression of the *FT*-like genes *Hd3a* and *RFT1* under SD conditions (Yano et al., 2000; Izawa et al., 2002; Kojima et al., 2002; Hayama et al., 2003; Ishikawa et al., 2011). NsCOL2 may also influence *NsFT* expression indirectly or in concert with other factors that are only present at a certain developmental stage, given that the downregulation of *NsFTa^rep^* was only detected in seedlings and the upregulation of *NsFTd^act^* was only observed in flowering plants. The potential indirect effect of NsCOL2 on *NsFTd^act^* expression is supported by the absence of CO-specific regulatory elements (COREs) in the *NsFTd^act^* promoter, 5 kb of which we checked *in silico* using PLANTPAN3.0 (Chow et al., 2019) for the COREs TGTG(N_2–3_) ATG (Tiwari et al., 2010) and TGTGGT (Abelenda et al., 2016). However, our experiments did not reveal any major influence of NtCOL2a and NtCOL2b on the regulation of flowering time in *N. tabacum*, whereas NsCOL2 was able to induce flowering under otherwise non-inductive SD conditions in *N. sylvestris*. The assumed inability of NtCOL2 to regulate flowering time in *N. tabacum* cv. SR1 may reflect the absence of the *NsFTd^act^* homolog *NtFT7^act^* in the SR1 genome (Beinecke et al., 2018). Although the two *N. tabacum* COL2 homologs are unlikely to be key regulators of the floral transition, the observed abundance of 3xc-myc-tagged NtCOL2a and NtCOL2b (at least during long days) suggests that their abundance is controlled by light and/or the circadian clock and that the proteins are not completely functionless, but may regulate other clock/photoperiod-dependent processes. This hypothesis is supported by the circadian expression profile of the genes. The involvement of StCO and StCOL1 in the control of photoperiod-dependent tuberization in potato demonstrates that such scenarios are possible (Navarro et al., 2011; González-Schain et al., 2012; Abelenda et al., 2016). Furthermore, the B-box (BBX) protein family is known to be involved in diverse developmental processes influenced by light, including shade avoidance, seedling de-etiolation, and photomorphogenesis (Crocco and Botto, 2013). The role of the other *NtBBX* genes remains elusive, Song et al. (2022) suggested a role in the response to multiple stresses and we assume that some of them may help to control the photoperiod-dependent expression of *FTs* in day-neutral *N. tabacum*. Preliminary computational analysis of the remaining *Nicotiana FT* promoter regions supports this by revealing the presence of putative COREs (**Supplementary Table S13**; Accession numbers in **Supplementary Table S11**). Recently, the expression of *SlCOL*, *SlCOL4a* and *SlCOL4b* in day-neutral tomato was negatively associated with flowering time (Yang et al., 2020). Thus, future studies should elucidate the role of the remaining uncharacterized tobacco *BBX* genes (e.g., *NtBBX3*–*NtBBX7*) to determine which other processes they might control in *N. tabacum.*

## Data availability statement

The raw data supporting the conclusions of this article will be made available by the authors, without undue reservation.

## Author contributions

FS, LG, DP and GN contributed to the conception and design of the study. FS, ML, MS, AS, SL and LG conducted the experiments. FS, LG and RT analyzed the data. DP and GN contributed the reagents, materials, and the analytical tools. FS and LG wrote the first draft of the manuscript. FS, LG, RT and GN wrote sections of the manuscript. All authors contributed to the article and approved the submitted version.

## References

[B1] Abelenda J. A. Cruz-Oró E. Franco-Zorrilla J. M. Prat S. (2016). Potato StCONSTANS-like1 suppresses storage organ formation by directly activating the FT-like StSP5G repressor. Curr. Biol. 26, 872–881. doi: 10.1016/j.cub.2016.01.066 26972319

[B2] Abelenda J. A. Navarro C. Prat S. (2014). Flowering and tuberization: a tale of two nightshades. Trends Plant Sci. 19, 115–122. doi: 10.1016/j.tplants.2013.09.010 24139978

[B3] Amaya I. Ratcliffe O. J. Bradley D. J. (1999). Expression of CENTRORADIALIS (CEN) and CEN-like genes in tobacco reveals a conserved mechanism controlling phase change in diverse species. Plant Cell 11, 1405–1418. doi: 10.1105/tpc.11.8.1405 10449576 PMC144298

[B4] An H. Roussot C. Suárez-López P. Corbesier L. Vincent C. Piñeiro M.. (2004). CONSTANS acts in the phloem to regulate a systemic signal that induces photoperiodic flowering of Arabidopsis. Development 131, 3615–3626. doi: 10.1242/dev.01231 15229176

[B5] Aoki S. Ito M. (2000). Molecular phylogeny of Nicotiana (Solanaceae) based on the nucleotide sequence of the matK gene. Plant Biol. 2, 316–324. doi: 10.1055/s-2000-3710

[B6] Ballerini E. S. Kramer E. M. (2011). In the light of evolution: A reevaluation of conservation in the CO–FT regulon and its role in photoperiodic regulation of flowering time. Front. Plant Sci. 2. doi: 10.3389/fpls.2011.00081 PMC3355682 22639612

[B7] Beinecke F. A. Grundmann L. Wiedmann D. R. Schmidt F. J. Caesar A. S. Zimmermann M.. (2018). The FT/FD-dependent initiation of flowering under long-day conditions in the day-neutral species Nicotiana tabacum originates from the facultative short-day ancestor Nicotiana tomentosiformis. Plant J. 96, 329–342. doi: 10.1111/tpj.14033 30030859

[B8] Ben-Naim O. Eshed R. Parnis A. Teper-Bamnolker P. Shalit A. Coupland G.. (2006). The CCAAT binding factor can mediate interactions between CONSTANS-like proteins and DNA. Plant J. 46, 462–476. doi: 10.1111/j.1365-313X.2006.02706.x 16623906

[B9] Bevan M. (1984). Binary Agrobacterium vectors for plant transformation. Nucleic Acids Res. 12, 8711–8721. doi: 10.1093/nar/12.22.8711 6095209 PMC320409

[B10] Blümel M. Dally N. Jung C. (2015). Flowering time regulation in crops-what did we learn from Arabidopsis? Curr. Opin. Biotechnol. 32C, 121–129. doi: 10.1016/j.copbio.2014.11.023 25553537

[B11] Bombarely A. Edwards K. D. Sanchez-Tamburrino J. Mueller L. A. (2012). Deciphering the complex leaf transcriptome of the allotetraploid species Nicotiana tabacum: a phylogenomic perspective. BMC Genomics 13, 406. doi: 10.1186/1471-2164-13-406 22900718 PMC3582432

[B12] Chen Z. Han Y. Ning K. Ding Y. Zhao W. Yan S.. (2018). Inflorescence development and the role of LsFT in regulating bolting in lettuce (Lactuca sativa L.). Front. Plant Sci. 8. doi: 10.3389/fpls.2017.02248 PMC5778503 29403510

[B13] Chow C.-N. Lee T.-Y. Hung Y.-C. Li G.-Z. Tseng K.-C. Liu Y.-H.. (2019). PlantPAN3.0: a new and updated resource for reconstructing transcriptional regulatory networks from ChIP-seq experiments in plants. Nucleic Acids Res. 47, D1155–D1163. doi: 10.1093/nar/gky1081 30395277 PMC6323957

[B14] Clarkson J. J. Lim K. Y. Kovarik A. Chase M. W. Knapp S. Leitch A. R. (2005). Long-term genome diploidization in allopolyploid Nicotiana section Repandae (Solanaceae). New Phytol. 168, 241–252. doi: 10.1111/j.1469-8137.2005.01480.x 16159337

[B15] Crocco C. D. Botto J. F. (2013). BBX proteins in green plants: Insights into their evolution, structure, feature and functional diversification. Gene 531, 44–52. doi: 10.1016/j.gene.2013.08.037 23988504

[B16] Doi K. Izawa T. Fuse T. Yamanouchi U. Kubo T. Shimatani Z.. (2004). Ehd1, a B-type response regulator in rice, confers short-day promotion of flowering and controls FT-like gene expression independently of Hd1. Genes Dev. 18, 926–936. doi: 10.1101/gad.1189604 15078816 PMC395851

[B17] Edwards K. Johnstone C. Thompson C. (1991). A simple and rapid method for the preparation of plant genomic DNA for PCR analysis. Nucleic Acids Res. 19, 1349. doi: 10.1093/nar/19.6.1349 2030957 PMC333874

[B18] Evan G. I. Lewis G. K. Ramsay G. Bishop J. M. (1985). Isolation of monoclonal antibodies specific for human c-myc proto-oncogene product. Mol. Cell. Biol. 5, 3610–3616. doi: 10.1128/mcb.5.12.3610 3915782 PMC369192

[B19] Fauser F. Schiml S. Puchta H. (2014). Both CRISPR/Cas-based nucleases and nickases can be used efficiently for genome engineering in Arabidopsis thaliana. Plant J. 79, 348–359. doi: 10.1111/tpj.12554 24836556

[B20] Fornara F. Panigrahi K. C. Gissot L. Sauerbrunn N. Rühl M. Jarillo J. A.. (2009). Arabidopsis DOF transcription factors act redundantly to reduce CONSTANS expression and are essential for a photoperiodic flowering response. Dev. Cell 17, 75–86. doi: 10.1016/j.devcel.2009.06.015 19619493

[B21] Garabagi F. Gilbert E. Loos A. McLean M. D. Hall J. C. (2012). Utility of the P19 suppressor of gene-silencing protein for production of therapeutic antibodies in Nicotiana expression hosts. Plant Biotechnol. J. 10, 1118–1128. doi: 10.1111/j.1467-7652.2012.00742.x 22984968

[B22] González-Schain N. D. Díaz-Mendoza M. Zurczak M. Suárez-López P. (2012). Potato CONSTANS is involved in photoperiodic tuberization in a graft-transmissible manner. Plant J. 70, 678–690. doi: 10.1111/j.1365-313X.2012.04909.x 22260207

[B23] Griffiths S. Dunford R. P. Coupland G. Laurie D. A. (2003). The evolution of CONSTANS-like gene families in barley, rice, and Arabidopsis. Plant Physiol. 131, 1855–1867. doi: 10.1104/pp.102.016188 12692345 PMC166942

[B24] Hamilton C. M. Frary A. Lewis C. Tanksley S. D. (1996). Stable transfer of intact high molecular weight DNA into plant chromosomes. Proc. Natl. Acad. Sci. 93, 9975–9979. doi: 10.1073/pnas.93.18.9975 8790442 PMC38540

[B25] Harig L. Beinecke F. A. Oltmanns J. Muth J. Müller O. Rüping B.. (2012). Proteins from the FLOWERING LOCUS T-like subclade of the PEBP family act antagonistically to regulate floral initiation in tobacco. Plant J. 72, 908–921. doi: 10.1111/j.1365-313X.2012.05125.x 22889438

[B26] Hayama R. Yokoi S. Tamaki S. Yano M. Shimamoto K. (2003). Adaptation of photoperiodic control pathways produces short-day flowering in rice. Nature 422, 719–722. doi: 10.1038/nature01549 12700762

[B27] Hoekema A. Hirsch P. R. Hooykaas P. J. J. Schilperoort R. A. (1983). A binary plant vector strategy based on separation of vir- and T-region of the Agrobacterium tumefaciens Ti-plasmid. Nature 303, 179–180. doi: 10.1038/303179a0

[B28] Horsch R. B. Fry J. E. Hoffmann N. L. Wallroth M. Eichholtz D. Rogers S. G.. (1985). A simple and general method for transferring genes into plants. Science 227, 1229–1231. doi: 10.1126/science.227.4691.1229 17757866

[B29] Hsu P. D. Scott D. A. Weinstein J. A. Ran F. A. Konermann S. Agarwala V.. (2013). DNA targeting specificity of RNA-guided Cas9 nucleases. Nat. Biotechnol. 31, 827–832. doi: 10.1038/nbt.2647 23873081 PMC3969858

[B30] Imaizumi T. (2010). Arabidopsis circadian clock and photoperiodism: time to think about location. Curr. Opin. Plant Biol. 13, 83–89. doi: 10.1016/j.pbi.2009.09.007 19836294 PMC2818179

[B31] Imaizumi T. Schultz T. F. Harmon F. G. Ho L. A. Kay S. A. (2005). FKF1 F-box protein mediates cyclic degradation of a repressor of CONSTANS in Arabidopsis. Science 309, 293–297. doi: 10.1126/science.1110586 16002617

[B32] Ishikawa R. Aoki M. Kurotani K.-I. Yokoi S. Shinomura T. Takano M.. (2011). Phytochrome B regulates Heading date 1 (Hd1)-mediated expression of rice florigen Hd3a and critical day length in rice. Mol. Genet. Genomics 285, 461–470. doi: 10.1007/s00438-011-0621-4 21512732

[B33] Izawa T. Oikawa T. Sugiyama N. Tanisaka T. Yano M. Shimamoto K. (2002). Phytochrome mediates the external light signal to repress FT orthologs in photoperiodic flowering of rice. Genes Dev. 16, 2006–2020. doi: 10.1101/gad.999202 12154129 PMC186415

[B34] Jang S. Marchal V. Panigrahi K. C. S. Wenkel S. Soppe W. Deng X.-W.. (2008). Arabidopsis COP1 shapes the temporal pattern of CO accumulation conferring a photoperiodic flowering response. EMBO J. 27, 1277–1288. doi: 10.1038/emboj.2008.68 18388858 PMC2291449

[B35] Jones P. Binns D. Chang H.-Y. Fraser M. Li W. McAnulla C.. (2014). InterProScan 5: genome-scale protein function classification. Bioinformatics 30, 1236–1240. doi: 10.1093/bioinformatics/btu031 24451626 PMC3998142

[B36] Jung C. Müller A. E. (2009). Flowering time control and applications in plant breeding. Trends Plant Sci. 14, 563–573. doi: 10.1016/J.TPLANTS.2009.07.005 19716745

[B37] Kitamura S. Inoue M. Ohmido N. Fukui K. (2000). Quantitative chromosome maps and rDNA localization in the T subgenome of Nicotiana tabacum L. and its putative progenitors. Theor. Appl. Genet. 101, 1180–1188. doi: 10.1007/s001220051595

[B38] Kobayashi Y. Kaya H. Goto K. Iwabuchi M. Araki T. (1999). A pair of related genes with antagonistic roles in mediating flowering signals. Science 286, 1960–1962. doi: 10.1126/science.286.5446.1960 10583960

[B39] Kobayashi Y. Weigel D. (2007). Move on up, it’s time for change-mobile signals controlling photoperiod-dependent flowering. Genes Dev. 21, 2371–2384. doi: 10.1101/gad.1589007 17908925

[B40] Kojima S. Takahashi Y. Kobayashi Y. Monna L. Sasaki T. Araki T.. (2002). Hd3a, a rice ortholog of the Arabidopsis FT gene, promotes transition to flowering downstream of Hd1 under short-day conditions. Plant Cell Physiol. 43, 1096–1105. doi: 10.1093/pcp/pcf156 12407188

[B41] Komiya R. Ikegami A. Tamaki S. Yokoi S. Shimamoto K. (2008). Hd3a and RFT1 are essential for flowering in rice. Development 135, 767–774. doi: 10.1242/dev.008631 18223202

[B42] Komiya R. Yokoi S. Shimamoto K. (2009). A gene network for long-day flowering activates RFT1 encoding a mobile flowering signal in rice. Development 136, 3443–3450. doi: 10.1242/dev.040170 19762423

[B43] Koncz C. Schell J. (1986). The promoter of TL-DNA gene 5 controls the tissue-specific expression of chimaeric genes carried by a novel type of Agrobacterium binary vector. Mol. Gen. Genet. 204, 383–396. doi: 10.1007/BF00331014

[B44] Koornneef M. Hanhart C. J. van der Veen J. H. (1991). A genetic and physiological analysis of late flowering mutants in Arabidopsis thaliana. Mol. Gen. Genet. 229, 57–66. doi: 10.1007/BF00264213 1896021

[B45] Laemmli U. K. (1970). Cleavage of structural proteins during the assembly of the head of bacteriophage T4. Nature 227, 680–685. doi: 10.1038/227680a0 5432063

[B46] Laubinger S. Marchal V. Le Gourrierec J. Wenkel S. Adrian J. Jang S.. (2006). Arabidopsis SPA proteins regulate photoperiodic flowering and interact with the floral inducer CONSTANS to regulate its stability. Development 133, 3213–3222. doi: 10.1242/dev.02481 16854975

[B47] Leitch I. J. Hanson L. Lim K. Y. Kovarik A. Chase M. W. Clarkson J. J.. (2008). The ups and downs of genome size evolution in polyploid species of Nicotiana (Solanaceae). Ann. Bot. 101, 805–814. doi: 10.1093/aob/mcm326 18222910 PMC2710205

[B48] Lifschitz E. Ayre B. G. Eshed Y. (2014). Florigen and anti-florigen - a systemic mechanism for coordinating growth and termination in flowering plants. Front. Plant Sci. 5. doi: 10.3389/fpls.2014.00465 PMC4165217 25278944

[B49] Liu L.-J. Zhang Y.-C. Li Q.-H. Sang Y. Mao J. Lian H.-L.. (2008). COP1-mediated ubiquitination of CONSTANS is implicated in cryptochrome regulation of flowering in Arabidopsis. Plant Cell 20, 292–306. doi: 10.1105/tpc.107.057281 18296627 PMC2276438

[B50] Livak K. J. Schmittgen T. D. (2001). Analysis of relative gene expression data using real-time quantitative PCR and the 2–ΔΔCT method. Methods 25, 402–408. doi: 10.1006/METH.2001.1262 11846609

[B51] Madeira F. Park Y. M. Lee J. Buso N. Gur T. Madhusoodanan N.. (2019). The EMBL-EBI search and sequence analysis tools. Nucleic Acids Res. 47, W636–W641. doi: 10.1093/nar/gkz268 30976793 PMC6602479

[B52] Madeira F. Pearce M. Tivey A. R. N. Basutkar P. Lee J. Edbali O.. (2022). Search and sequence analysis tools services from EMBL-EBI. Nucleic Acids Res. 50, W276–W279. doi: 10.1093/nar/gkac240 35412617 PMC9252731

[B53] McGarry R. C. Ayre B. G. (2012). Manipulating plant architecture with members of the CETS gene family. Plant Sci. 188-189, 71–81. doi: 10.1016/j.plantsci.2012.03.002 22525246

[B54] Murad L. Lim K. Y. Christopodulou V. Matyasek R. Lichtenstein C. P. Kovarik A.. (2002). The origin of tobacco’s T genome is traced to a particular lineage within Nicotiana tomentosiformis (Solanaceae). Am. J. Bot. 89, 921–928. doi: 10.3732/ajb.89.6.921 21665691

[B55] Murashige T. Skoog F. (1962). A revised medium for rapid growth and bio assays with tobacco tissue cultures. Physiologia Plantarum 15, 473–497. doi: 10.1111/j.1399-3054.1962.tb08052.x

[B56] Nagai T. Ibata K. Park E. S. Kubota M. Mikoshiba K. Miyawaki A. (2002). A variant of yellow fluorescent protein with fast and efficient maturation for cell-biological applications. Nat. Biotechnol. 20, 87–90. doi: 10.1038/nbt0102-87 11753368

[B57] Navarro C. Abelenda J. A. Cruz-Oró E. Cuéllar C. A. Tamaki S. Silva J.. (2011). Control of flowering and storage organ formation in potato by FLOWERING LOCUS T. Nature 478, 119–122. doi: 10.1038/nature10431 21947007

[B58] Noll G. A. Fontanellaz M. E. Rüping B. Ashoub A. van Bel A. J. E. Fischer R.. (2007). Spatial and temporal regulation of the forisome gene for1 in the phloem during plant development. Plant Mol. Biol. 65, 285–294. doi: 10.1007/s11103-007-9217-0 17694275

[B59] Okamuro J. K. Goldberg R. B. (1985). Tobacco single-copy DNA is highly homologous to sequences present in the genomes of its diploid progenitors. Mol. Gen. Genet. 198, 290–298. doi: 10.1007/BF00383009

[B60] Pin P. A. Benlloch R. Bonnet D. Wremerth-Weich E. Kraft T. Gielen J. J. L.. (2010). An antagonistic pair of FT homologs mediates the control of flowering time in sugar beet. Science 330, 1397–1400. doi: 10.1126/science.1197004 21127254

[B61] Putterill J. Robson F. Lee K. Simon R. Coupland G. (1995). The CONSTANS gene of Arabidopsis promotes flowering and encodes a protein showing similarities to zinc finger transcription factors. Cell 80, 847–857. doi: 10.1016/0092-8674(95)90288-0 7697715

[B62] Robson F. Costa M. M. Hepworth S. R. Vizir I. Piñeiro M. Reeves P. H.. (2001). Functional importance of conserved domains in the flowering-time gene CONSTANS demonstrated by analysis of mutant alleles and transgenic plants. Plant J. 28, 619–631. doi: 10.1046/j.1365-313x.2001.01163.x 11851908

[B63] Rodríguez-Falcón M. Bou J. Prat S. (2006). Seasonal control of tuberization in potato: conserved elements with the flowering response. Annu. Rev. Plant Biol. 57, 151–180. doi: 10.1146/annurev.arplant.57.032905.105224 16669759

[B64] Romero-Calvo I. Ocón B. Martínez-Moya P. Suárez M. D. Zarzuelo A. Martínez-Augustin O.. (2010). Reversible Ponceau staining as a loading control alternative to actin in Western blots. Analytical Biochem. 401, 318–320. doi: 10.1016/J.AB.2010.02.036 20206115

[B65] Samach A. Onouchi H. Gold S. E. Ditta G. S. Schwarz-Sommer Z. Yanofsky M. F.. (2000). Distinct roles of CONSTANS target genes in reproductive development of Arabidopsis. Science 288, 1613–1616. doi: 10.1126/science.288.5471.1613 10834834

[B66] Sawa M. Nusinow D. A. Kay S. A. Imaizumi T. (2007). FKF1 and GIGANTEA complex formation is required for day-length measurement in Arabidopsis. Science 318, 261–265. doi: 10.1126/science.1146994 17872410 PMC3709017

[B67] Schmidt G. W. Delaney S. K. (2010). Stable internal reference genes for normalization of real-time RT-PCR in tobacco (Nicotiana tabacum) during development and abiotic stress. Mol. Genet. Genomics 283, 233–241. doi: 10.1007/s00438-010-0511-1 20098998

[B68] Schmidt F. J. Zimmermann M. M. Wiedmann D. R. Lichtenauer S. Grundmann L. Muth J.. (2020). The major floral promoter NtFT5 in tobacco (Nicotiana tabacum) is a promising target for crop improvement. Front. Plant Sci. 10. doi: 10.3389/fpls.2019.01666 PMC6966700 31998348

[B69] Shalit A. Rozman A. Goldshmidt A. Alvarez J. P. Bowman J. L. Eshed Y.. (2009). The flowering hormone florigen functions as a general systemic regulator of growth and termination. Proc. Natl. Acad. Sci. 106, 8392–8397. doi: 10.1073/PNAS.0810810106 19416824 PMC2688896

[B70] Sheerin D. J. Menon C. zur Oven-Krockhaus S. Enderle B. Zhu L. Johnen P.. (2015). Light-activated phytochrome A and B interact with members of the SPA family to promote photomorphogenesis in Arabidopsis by reorganizing the COP1/SPA complex. Plant Cell 27, 189–201. doi: 10.1105/tpc.114.134775 25627066 PMC4330587

[B71] Sierro N. Battey J. N. D. Ouadi S. Bakaher N. Bovet L. Willig A.. (2014). The tobacco genome sequence and its comparison with those of tomato and potato. Nat. Commun. 5, 3833. doi: 10.1038/ncomms4833 24807620 PMC4024737

[B72] Sierro N. Battey J. N. D. Ouadi S. Bovet L. Goepfert S. Bakaher N.. (2013). Reference genomes and transcriptomes of Nicotiana sylvestris and Nicotiana tomentosiformis. Genome Biol. 14, R60–R60. doi: 10.1186/gb-2013-14-6-r60 23773524 PMC3707018

[B73] Simon S. Rühl M. Montaigu A. de, Wötzel S. Coupland G. (2015). Evolution of CONSTANS regulation and function after gene duplication produced a photoperiodic flowering switch in the Brassicaceae. Mol. Biol. Evol. 32, 2284–2301. doi: 10.1093/molbev/msv110 25972346 PMC4540966

[B74] Skalická K. Lim K. Y. Matyasek R. Matzke M. Leitch A. R. Kovarik A. (2005). Preferential elimination of repeated DNA sequences from the paternal, Nicotiana tomentosiformis genome donor of a synthetic, allotetraploid tobacco. New Phytol. 166, 291–303. doi: 10.1111/j.1469-8137.2004.01297.x 15760371

[B75] Smykal P. Gennen J. Bodt S. de, Ranganath V. Melzer S. (2007). Flowering of strict photoperiodic Nicotiana varieties in non-inductive conditions by transgenic approaches. Plant Mol. Biol. 65, 233–242. doi: 10.1007/s11103-007-9211-6 17660946

[B76] Song K. Li B. Wu H. Sha Y. Qin L. Chen X.. (2022). The Function of BBX Gene Family under Multiple Stresses in Nicotiana tabacum. Genes 13, 1841–1858. doi: 10.3390/genes13101841 36292726 PMC9602306

[B77] Song Y. H. Shim J. S. Kinmonth-Schultz H. A. Imaizumi T. (2014). Photoperiodic flowering: time measurement mechanisms in leaves. Annu. Rev. Plant Biol. 66, 441–464. doi: 10.1146/annurev-arplant-043014-115555 25534513 PMC4414745

[B78] Song Y. H. Smith R. W. To B. J. Millar A. J. Imaizumi T. (2012). FKF1 conveys timing information for CONSTANS stabilization in photoperiodic flowering. Science 336, 1045–1049. doi: 10.1126/science.1219644 22628657 PMC3737243

[B79] Stemmer M. Thumberger T. Del Sol Keyer M. Wittbrodt J. Mateo J. L. Maas S. (2015). CCTop: An intuitive, flexible and reliable CRISPR/Cas9 target prediction tool. PloS One 10, e0124633–e0124633. doi: 10.1371/journal.pone.0124633 25909470 PMC4409221

[B80] Suárez-López P. Wheatley K. Robson F. Onouchi H. Valverde F. Coupland G. (2001). CONSTANS mediates between the circadian clock and the control of flowering in Arabidopsis. Nature 410, 1116–1120. doi: 10.1038/35074138 11323677

[B81] Takada S. Goto K. (2003). TERMINAL FLOWER2, an Arabidopsis homolog of HETEROCHROMATIN PROTEIN1, counteracts the activation of FLOWERING LOCUS T by CONSTANS in the vascular tissues of leaves to regulate flowering time. Plant Cell 15, 2856–2865. doi: 10.1105/tpc.016345 14630968 PMC282816

[B82] Tamaki S. Matsuo S. Wong H. L. Yokoi S. Shimamoto K. (2007). Hd3a protein is a mobile flowering signal in rice. Science 316, 1033–1036. doi: 10.1126/science.1141753 17446351

[B83] Tamura K. Stecher G. Kumar S. (2021). MEGA 11: Molecular Evolutionary Genetics Analysis version 11. Molecular Biology and Evolution 38, 3022–3027. doi: 10.1093/molbev/msab120 33892491 PMC8233496

[B84] Tiwari S. B. Shen Y. Chang H.-C. Hou Y. Harris A. Ma S. F.. (2010). The flowering time regulator CONSTANS is recruited to the FLOWERING LOCUS T promoter via a unique cis-element. New Phytol. 187, 57–66. doi: 10.1111/j.1469-8137.2010.03251.x 20406410

[B85] Towbin H. Staehelin T. Gordon J. (1979). Electrophoretic transfer of proteins from polyacrylamide gels to nitrocellulose sheets: Procedure and some applications. Proc. Natl. Acad. Sci. 76, 4350–4354. doi: 10.1073/pnas.76.9.4350 388439 PMC411572

[B86] Tsuji H. Taoka K. I. Shimamoto K. (2011). Regulation of flowering in rice: Two florigen genes, a complex gene network, and natural variation. Curr. Opin. Plant Biol. 14, 45–52. doi: 10.1016/j.pbi.2010.08.016 20864385

[B87] Tsuji H. Taoka K. Shimamoto K. (2013). Florigen in rice: complex gene network for florigen transcription, florigen activation complex, and multiple functions. Curr. Opin. Plant Biol. 16, 228–235. doi: 10.1016/J.PBI.2013.01.005 23453779

[B88] Valverde F. Mouradov A. Soppe W. Ravenscroft D. Samach A. Coupland G. (2004). Photoreceptor regulation of CONSTANS protein in photoperiodic flowering. Science 303, 1003–1006. doi: 10.1126/science.1091761 14963328

[B89] Valverde F. (2011). CONSTANS and the evolutionary origin of photoperiodic timing of flowering. JXB 62, 2453–2463. doi: 10.1093/jxb/erq449 21239381

[B90] Wang H. Ma L. G. Li J. M. Zhao H. Y. Deng X. W. (2001). Direct interaction of Arabidopsis cryptochromes with COP1 in light control development. Science 294, 154–158. doi: 10.1126/science.1063630 11509693

[B91] Wang G. Wang P. Gao Y. Li Y. Wu L. Gao J.. (2018). Isolation and functional characterization of a novel FLOWERING LOCUS T homolog (NtFT5) in Nicotiana tabacum. J. Plant Physiol. 231, 393–401. doi: 10.1016/J.JPLPH.2018.10.021 30391867

[B92] Wickland D. P. Hanzawa Y. (2015). The FLOWERING LOCUS T/TERMINAL FLOWER 1 gene family: Functional evolution and molecular mechanisms. Mol. Plant 8, 983–997. doi: 10.1016/j.molp.2015.01.007 25598141

[B93] Wigge P. A. Kim M. C. Jaeger K. E. Busch W. Schmid M. Lohmann J. U.. (2005). Integration of spatial and temporal information during floral induction in Arabidopsis. Science 309, 1056–1059. doi: 10.1126/science.1114358 16099980

[B94] Xue W. Xing Y. Weng X. Zhao Y. Tang W. Wang L.. (2008). Natural variation in Ghd7 is an important regulator of heading date and yield potential in rice. Nat. Genet. 40, 761–767. doi: 10.1038/ng.143 18454147

[B95] Yang T. He Y. Niu S. Yan S. Zhang Y. (2020). Identification and characterization of the CONSTANS (CO)/CONSTANS-like (COL) genes related to photoperiodic signaling and flowering in tomato. Plant Sci. 301, 110653. doi: 10.1016/j.plantsci.2020.110653 33218623

[B96] Yano M. Katayose Y. Ashikari M. Yamanouchi U. Monna L. Fuse T.. (2000). Hd1, a major photoperiod sensitivity quantitative trait locus in rice, is closely related to the Arabidopsis flowering time gene CONSTANS. Plant Cell 12, 2473–2484. doi: 10.1105/tpc.12.12.2473 11148291 PMC102231

[B97] Zhao X. Yu F. Guo Q. Wang Y. Zhang Z. Liu Y. (2022). Genome-wide identification, characterization, and expression profile analysis of CONSTANS-like genes in woodland strawberry (Fragaria vesca). Front. Plant Sci. 13. doi: 10.3389/fpls.2022.931721 PMC9318167 35903224

[B98] Zuo Z. Liu H. Liu B. Liu X. Lin C. (2011). Blue light-dependent interaction of CRY2 with SPA1 regulates COP1 activity and floral initiation in Arabidopsis. Curr. Biol. 21, 841–847. doi: 10.1016/j.cub.2011.03.048 21514160 PMC3150455

