# Supplementary material for: COL2-dependent photoperiodic floral induction in Nicotiana sylvestris seems to be lost in the N. sylvestris × N. tomentosiformis hybrid N. tabacum
Source: Front Plant Sci. 2024 Jan 4;14:1249879. doi: 10.3389/fpls.2023.1249879 (PMC10794312; doi:10.3389/fpls.2023.1249879)
Supplement: Supplementary file 1 [file DataSheet_1.pdf]

Supplementary Figures, Materials and Methods

COL2-dependent photoperiodic floral induction in *Nicotiana sylvestris* seems to be lost in the *N. sylvestris* × *N. tomentosiformis* hybrid *N. tabacum*

Florentin J. Schmidt[1†], Lena Grundmann[2†], Michael Lahme[2], Marvin Seidemann[1], Axel Schwarze[1], Sophie Lichtenauer[1], Richard M. Twyman[3], Dirk Prüfer[1,2], Gundula A. Noll[1,2]

Supplementary Figures

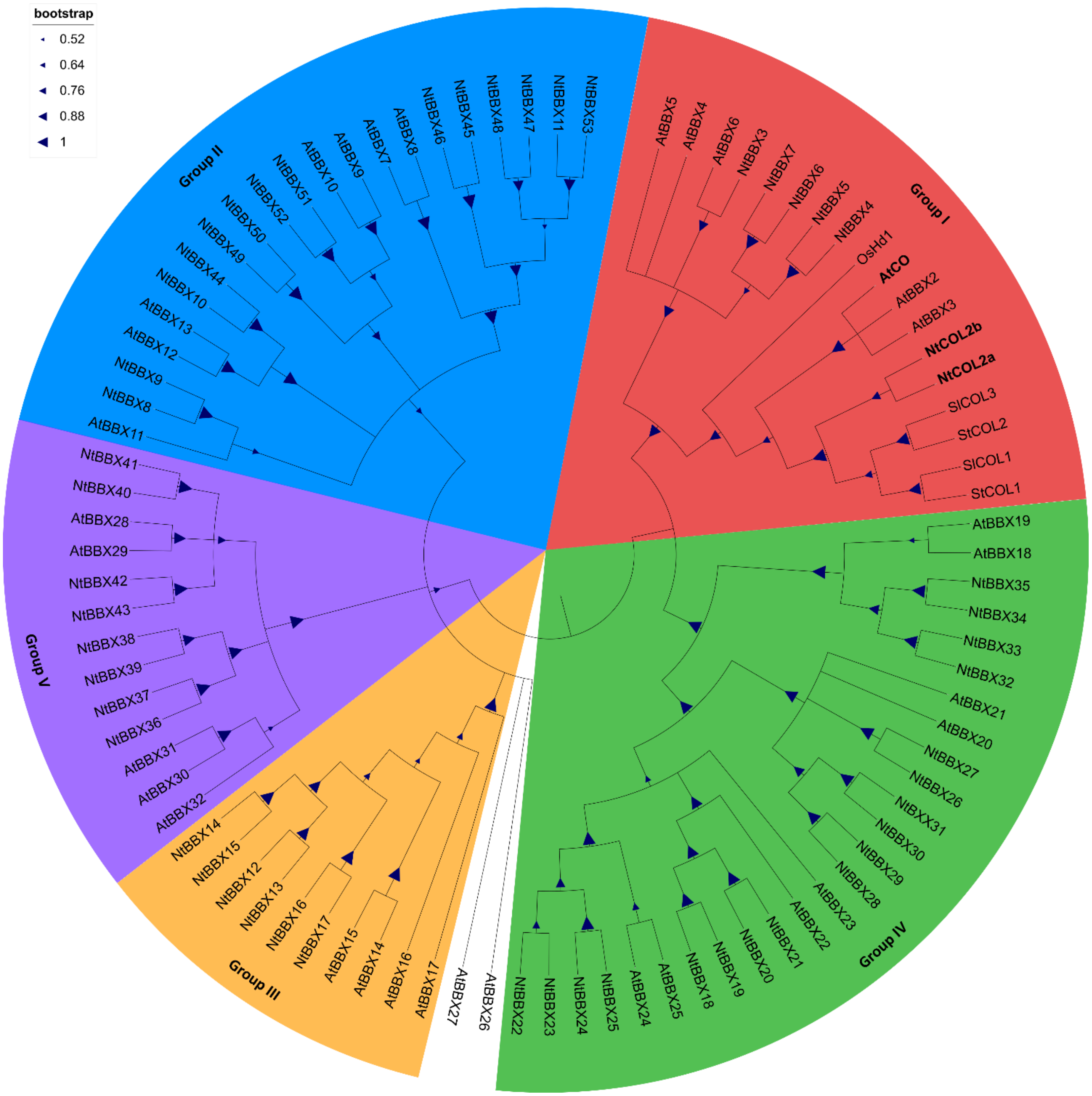

Supplementary Figure S1. Phylogenetic tree of CO(L)/BBX proteins from tobacco (Nt), Arabidopsis (At), rice (Os), tomato (Sl) and potato (St). The corresponding protein sequences (for accession numbers, see Supplementary Tables S9 and S11) were aligned with MEGA-11 software (Tamura et al., 2021) using ClustalW and this alignment was used to construct the phylogenetic tree using the Neighbor-Joining (NJ) method and 1.000 bootstrap replicates. The phylogenetic tree was then generated with the iTOL online tool (https://itol.embl.de/). The tree is divided into five groups, and the bootstrap values are indicated by the size of triangles.

```
                                                   B-box1
AtBBX17     --------------MISKY---QEDVKQ---------PRACELCLNK-HAVWYCASDDAF   33
AtBBX16     ----------MVVDVESRTASVTGEKMA---------ARGCDACMKRSRASWYCPADDAF   41
AtBBX14     --------------MMKSLASAVGGK-T---------ARACDSCVKR-RARWYCAADDAF   35
AtBBX15     --------------MMKSLANAVGAK-T---------ARACDSCVKR-RARWYCAADDAF   35
NtBBX16     -----------MMTSESKTANAIGGK-T---------ARACDCCLSK-RARWFCPADDAF   38
NtBBX14     ------------MVSDRKLASAMGGK-T---------TRACDNCIRK-RARWYCPADDAF   37
NtBBX12     -----------MTSSEKKIANVVGAK-T---------ARACDSCIRK-RARWYCAADDAF   38
OsHd1       MNYNFGGNVFDQEVGVGGEGGGGGEGSGCPW------ARPCDGCRAA-PSVVYCRADAAY   53
SlCOL3      --------------MLKKENSGGLDGSSNYW------ARVCDSCRSV-TCTIYCQADSAY   39
StCOL2      --------------MLKKENSGGFDGSSNYW------ARVCDSCRLV-TCTIYCQADSAY   39
NtCOL2b     --------------MLKKE------NSSNNW------ARVCDTCRSA-ACTVYCRADSAY   33
NsCOL2      --------------MLKKE------NSSNNW------ARVCDTCRSA-ACTVYCRADSAY   33
NtCOL2a     --------------MLKKE------NSSNNW------AKVCDTCRST-ACTVYCRADSAY   33
NtomCOL2    --------------MLKKE------NSSNNW------AKVCDTCRST-ACTVYCRADSAY   33
SlCOL1      --------------MLKK-------ENSNNW------ARVCDSCHSA-TCTVYCRADSAY   32
StCO        --------------MLKKEKSGGFDRSSNNW------ARVCDSCHSA-TCTVYCRADSAY   39
StCOL1      --------------MLKKEKSGGFDGSSNNW------ARVCDSCHSA-TCTVYCRADSAY   39
AtBBX2      --------------ML---------KVESNW------AQACDTCRSA-ACTVYCRADSAY   30
AtCO        --------------MLK-QESNDIGSGENNR------ARPCDTCRSN-ACTVYCHADSAY   38
AtBBX3      --------------MLK-EESNESG----TW------ARACDTCRSA-ACTVYCEADSAY   34
NtBBX3      -------------MG---ILRGGANCFSGGWGAAAAVAKPCEYCHLD-AALVFCRTDNMF   43
AtBBX6      -------------MG---FGLESIKSISGGWGAA---ARSCDACKSV-TAAVFCRVDSAF   40
NtBBX4      -------------------------MGSENWSLT---VKLCDSCKTT-SATVFCRADSAF   31
NtBBX6      -------------------------MVAESWSAT---AKRCDSCKST-AATVFCKADMAF   31
AtBBX4      ------------------------------MASS---SRLCDSCKST-AATLFCRADAAF   26
AtBBX5      --------------------------------MA---SKLCDSCKSA-TAALYCRPDAAF   24
NtBBX46     ------------------------------------MGYICEFCGEQ-RSIVYCRSDAAC   23
NtBBX48     ------------------------------------MGHICEFCGEQ-RSIVYCRSDAAC   23
NtBBX11     ------------------------------------MGFTCEFCGEQ-RSIVYCRSDAAC   23
AtBBX7      ------------------------------------MGYMCDFCGEQ-RSMVYCRSDAAC   23
AtBBX8      ------------------------------------MGYMCDFCGEQ-RSMVYCRSDAAC   23
AtBBX9      ------------------------------------MEARCDFCGTE-KALIYCKSDSAK   23
AtBBX10     --------------------------MS------PSMEPKCDHCATS-QALIYCKSDLAK   27
NtBBX49     ------------------------------------MEPLCDVCGVE-RAVVYCKSDAAK   23
NtBBX52     ------------------------------------MDPVCELCGVV-RGLVYCKSDSAR   23
AtBBX11     ----------------------MEAEEG------HQRDRLCDYCDSS-VALVYCKADSAK   31
NtBBX9      ---------------MTELKEDQEDQNQ------QPQKRLCDFCGEN-TALLYCRADSAK   38
NtBBX44     -----------------------MSQRN------TGDSRPCDFCNQQ-IAVLYCRADSAK   30
AtBBX12     -----------------------MGTST------TESVVACEFCGER-TAVLFCRADTAK   30
AtBBX13     --------------------------MS------SSERVPCDFCGER-TAVLFCRADTAK   27
                                                    *: *        :*  *
```

```
                B-box1                    ----------B-box2---------------------
AtBBX17   LCHVCDESVHSANHVATKHERVCL RTNEISNDVR---------------------GGTTL    72
AtBBX16   LCQSCDASIHSANHLAKRHERVRL QSSSPTETADK------------------------T    77
AtBBX14   LCHACDGSVHSANPLARRHERVRL KSASAGKYRHA--------------------S-PPH    74
AtBBX15   LCQSCDSLVHSANPLARRHERVRL KTASPAVVKHSNH---------------SSASPPHE    80
NtBBX16   LCQSCDVSVHSANQLASRHERVRL ETSSNKSTDTK--F----------------VDQTVS    80
NtBBX14   LCQNCDSSVHSANPLARRHERVRL KTSPLKESSPDENFPN------------LESPVSVS    85
NtBBX12   LCQSCDSSVHSANPLARRHERVRL KTSSLKSSDE--------------------FSNSVS    78
OsHd1     LCASCDARVHAANRVASRHERVRV CEACERAPAALACRADAAALCVACDVQVHSANP---    110
SlCOL3    LCADCDARIHAASLVTSRHKRVWV CEACERAPAAFLCKADAASLCASCDADIHSANP---    96
StCOL2    LCAGCDARIHAASLVASRHKRVWV CEACECAPAAFLCKADAASLCASCDADIHSANP---    96
NtCOL2b   LCAGCDARIHAANLVASRHERVWV CEACERAPAAFLCKADAASLCASCDADIHSANP---    90
NsCOL2    LCAGCDARIHAANLVASRHERVWV CEACERAPAAFLCKADAASLCASCDADIHSANP---    90
NtCOL2a   LCAGCDARIHAANLVASRHERVWV CEACERAPAAFLCKADAASLCASCDADIHSANP---    90
NtomCOL2  LCAGCDARIHAANLVASRHERVWV CEACERAPAAFLCKADAASLCASCDADIHSANP---    90
SlCOL1    LCAGCDARIHTASLMASRHERVWV CEACERAPAAFLCKADAASLCASCDADIHSANP---    89
StCO      LCAGCDSRIHAASLMASRHERVWV CEACERAPAAFLCKADAASLCASCDAVIHSANP---    96
StCOL1    LCADCDARIHAASLMASRHERVWV CEACERAPAAFLCKADAASLCASCDVDIHSANP---    96
AtBBX2    LCSSCDAQVHAANRLASRHERVRV CQSCERAPAAFFCKADAASLCTTCDSEIHSANP---    87
AtCO      LCMSCDAQVHSANRVASRHKRVRV CESCERAPAAFLCEADDASLCTACDSEVHSANP---    95
AtBBX3    LCTTCDARVHAANRVASRHERVRV CQSCESAPAAFLCKADAASLCTACDAEIHSANP---    91
NtBBX3    MCLGCDTRVHGN----ARHERVWM CEVCEQAAASVTCKADAAALCLSCDRDIHSANP---    96
AtBBX6    LCIACDTRIHSF----TRHERVWV CEVCEQAPAAVTCKADAAALCVSCDADIHSANP---    93
NtBBX4    LCLACDCKIHAANKLASRHARVWV CEVCEQAPASVTCKADAAALCVTCDRDIHSANP---    88
NtBBX6    LCLSCDSKIHAANKLASRHARVWV CEVCEHAPASVTCKADAAALCVTCDQDIHSANP---    88
AtBBX4    LCGDCDGKIHTANKLASRHERVWL CEVCEQAPAHVTCKADAAALCVTCDRDIHSANP---    83
AtBBX5    LCLSCDSKVHAANKLASRHARVWM CEVCEQAPAHVTCKADAAALCVTCDRDIHSANP---    81
NtBBX46   LCLSCDRNVHSANALSERHSRTLI CERCNSQPAVVRCIEEKTSLCQNCDWSGHASSSS--    81
NtBBX48   LCLSCDRNVHSANALSQRHSRTLL CERCNSQPAVVRRVEEKISLCKNCDSIGHA------    77
NtBBX11   LCLSCDRHVHSANALSQRHSRTLV CERCNSQPAIFRCDEDRISLCQNCDWIGHANS-S--    80
AtBBX7    LCLSCDRSVHSANALSKRHSRTLV CERCNAQPATVRCVEERVSLCQNCDWSGHNNSNNNN    83
AtBBX8    LCLSCDRNVHSANALSKRHSRTLV CERCNAQPASVRCSDERVSLCQNCDWSGHDGKNS--    81
AtBBX9    LCLNCDVNVHSANPLSQRHTRSLL CEKCSLQPTAVHCMNENVSLCQGCQWTASNCT----    79
AtBBX10   LCLNCDVHVHSANPLSHRHIRSLI CEKCFSQPAAIRCLDEKVSYCQGCHWHESNCS----    83
NtBBX49   LCFPCDGSVHSANCLSRKHVRSLI CDNCSSEPAIVRCMIIVMCLCEGCDSNTIGCN----    79
NtBBX52   LCLQCDENVHSANCLSRRHSRSLI CDKCSSQPAVVRCMDEEMCLCESCDWNENGCT----    79
AtBBX11   LCLACDKQVHVANQLFAKHFRSLL CDSCNESPSSLFCETERSVLCQNCDWQHHTA-S---    87
NtBBX9    LCFSCDREVHSTNQLFTKHTRWLL CDSCDSSPASILCCTDSCVFCQNCDWESHNL-S---    94
NtBBX44   LCLFCDQHVHSANALSKKHLRSQI CDNCCSEPVSIRCATDNLVLCQECDWDAHGSCA---    87
AtBBX12   LCLPCDQHVHSANLLSRKHVRSQI CDNCSKEPVSVRCFTDNLVLCQECDWDVHGSCS---    87
AtBBX13   LCLPCDQQVHTANLLSRKHVRSQI CDNCGNEPVSVRCFTDNLILCQECDWDVHGSCS---    84
          :*  **  :*        :* *  :
```

```
                 ----B-box2------
AtBBX17          TSVWHSGFRRKARTPRSRYEKKPQQKIDD-----ERR----R-EDPRVPEI---------      113
AtBBX16          TSVWYEGFRRKARTPRSKSCAFEKL--------LQIE----S-NDPLVPEL---------      115
AtBBX14          QATWHQGFTRKARTPRGG-KKSHTM----------------V-FHDLVPEM---------      107
AtBBX15          VATWHHGFTRKARTPRGSGKKNNSS----------------I-FHDLVPDI---------      114
NtBBX16          QPSWHQGFTRKARTPRNGKNKSTQIGQRKK-----------D-EENMVPEM---------      119
NtBBX14          LPSWHRGFTRKARTPRNGRKVSKSTEEEEEEEVILKN----P-I-HLVPEI---------      130
NtBBX12          VPSWHRGFTRKARTPRNGKNSRK-----------VKN----P-IQLVVPEI---------      113
OsHd1            ------L-------PAITIPATSVLAEAVVAT----------------------------      129
SlCOL3           ------LAHRHHRIPIITIPGTLYGPPAVETVGGDSMMI---------------SGSTGE      135
StCOL2           ------LAHRHHRIPIIPIPGTLYGPPAVDTVGGDSMMI---------------GGSTGE      135
NtCOL2b          ------LARRHHRVPIIPIPGTLYGPPAVDTLGGGTLMI---------------GGPEGD      129
NsCOL2           ------LARRHHRVPIIPIPGTLYGPPAVDTLGGGTLMI---------------GGPEGD      129
NtCOL2a          ------LARRHHRVPIMPIPGTLYGPPAVDTLSGGTLMI---------------GGPEGD      129
NtomCOL2         ------LARRHHRVPIMPIPGTLYGPPAVDTLSGGTLMI---------------GGPEGD      129
SlCOL1           ------LARRHHRVPIMPIPGTIYGPPAVHTITGGSMMI---------------GGTTGE      128
StCO             ------LARRHHRVPIMPIPGTLYGPPAVHTVSGGSMMI---------------GGTTGE      135
StCOL1           ------LARRHHRVPIMPIPGTLYGPPAVHTVSGGSMMI---------------GGTTGE      135
AtBBX2           ------LARRHQRVPILPISEYSYSSTAT-NHSCETTV----------------------      118
AtCO             ------LARRHQRVPILPISGNSFSSMTTTHHQSEKTM----------------------      127
AtBBX3           ------LARRHQRVPILPLSANSCSSMAPSET----------------------------      117
NtBBX3           ------LAQRHERIPVVPFYDPVESVVKSTAATLLVSIN---------------NSNSTS      135
AtBBX6           ------LASRHERVPVETFFDSAETAVAKISASSTFGIL---------------GS----      128
NtBBX4           ------LARRHERFPVVPFYDSAVAKSHGVNDEEK-------------------------      117
NtBBX6           ------LARRHDRFPIVPFYDSASTKSRGADEI---------------------------      115
AtBBX4           ------LSRRHERVPITPFYDAVGPAKSAS-SSVNF--V---------------D-----      114
AtBBX5           ------LARRHERVPVTPFYDSVSSDGSVKHTAVNF--L---------------DD----      114
NtBBX46          ------SGSTHMRQALSSYTGCPSAAELSNSWSFLLDDP--SIGA-------SCEQRMGT      126
NtBBX48          ------AGSMHKRQALSCYTGCPSAAELSTIWSFLLD------GD------STCQKGMGA      119
NtBBX11          ------SGSTHKRQALSCYTGCPSAVELSVIWSFLLDNP--SVGD------STCEQGMSS      126
AtBBX7           SSSSSTSPQQHKRQTISCYSGCPSSSELASIWSFCLDLA----GQ------SICEQELGM      133
AtBBX8           -----TTTSHHKRQTINCYSGCPSSAELSSIWSFCMDLNISSAEE------SACEQGMGL      130
AtBBX9           ------GLG-HRLQSLNPYSDCPSPSDFGKIWSSTLEP--S---V------TSLVSPFS-      120
AtBBX10          ------ELG-HRVQSLNPFSGCPSPTDFNRMWSSILEP--P---V------SGLLSPFV-      124
NtBBX49          ------MVSRHQHQKLDSYTGCPSPAEFMTKILSTTFDQMPNNNV------HNV------      121
NtBBX52          ------GTG-HRLKQLNPYNGSLSPTEFTRMLSE-V-LEIPDTNF------GSFSTPCSS      124
AtBBX11          -------SSLHSRRPFEGFTGCPSVPELLAIVGLDDLTL---------------------      119
NtBBX9           ------LSPAHERRPLEGFTGCPSVSELLSILGFEDLGKKALLYGD------SVTSDGGG      142
NtBBX44          ----------HDRTPVEGFSGCPCASELASAWGLDVEAKKPPTPTHLQPTWNGFLEPCNW      137
AtBBX12          ------SSATHERSAVEGFSGCPSVLELAAVWGIDLKGKKKEDDE------DELTKN-FG      134
AtBBX13          ------VSDAHVRSAVEGFSGCPSALELAALWGLDLEQGRKDEENQVP-MMAMMMDN-FG      136
```

```
AtBBX17    -GGEVM-----FFIP---------EANDDDMTSLVPEFEGFTEMGFFLSNHNGTEETT--    156
AtBBX16    -GGDEDDGFFSFSSV---------EETEESLNCCVPVFDPFSDMLIDDINGFCL--VPDE    163
AtBBX14    -STEDQ------AES---------YEVEEQLIFEVPVMNSMVEEQCFNQSLEKQNEFP--    149
AtBBX15    -SIEDQ------TDN---------YELEEQLICQVPVLDPLVSEQFLNDVVEPKIEFP--    156
NtBBX16    -GSEENSPD---ENE---------FENEEQLLYRVPVFDPFEAELCCTVPNEVV-DETTT    165
NtBBX14    -LSDENSLD----------------ENEEQLLYRVPIFDPFVAELCTSATRNDQIENVQT    173
NtBBX12    -LSDENSQD----------------DNEEQLLYRVPILDPFVADDAIGNEY---------    147
OsHd1      ---------------------------ATVLGDKDEEVDSWLL----LSKDSDN----NN    154
SlCOL3     GTEDDGFLSLTQDAD---------DTIIDEEDEDEDEAASWLL----LNHPVKN----NN    178
StCOL2     GTEDDGFLSLTQDAD---------DTTIDEED--EDEAASWLL----LNHPVKD----NN    176
NtCOL2b    ATEDDRFLSLTQGAD---------DTTIDEED--EDEAASWLL----LNPPVKN----NN    170
NsCOL2     ATEDDRFLSLTQGAD---------DTTIDEED--EDEAASWLL----LNPPVKN----NN    170
NtCOL2a    ATEDDGFLSLTQDAD---------DTTIDEED--KDEAASWLL----LNLPVKN----NN    170
NtomCOL2   ATEDDGFLSLTQDAD---------DTTIDEED--KDEAASWLL----LNLPVKN----NN    170
SlCOL1     GTEDDGFLSLNQDAD---------DTTIDEED--EDEAASWLL----LNPPVKN----NN    169
StCO       GTEDDGFLSLTQDAD---------DTTIDEED--ENEAASWLL----LNPPVKN----NN    176
StCOL1     GTEDDGFLSLTQDAD---------DTTIDEED--ENEAASWLL----LNPPVKN----NN    176
AtBBX2     -TDPENRLVLGQE-------------E---EDEDEAEAASWLL----PNSGKNS----G-    152
AtCO       -TDPEKRLVVDQE-------------EGEEGDKDAKEVASWLF----PNSDKNN----N-    164
AtBBX3     ---------------------------DADNDEDDREVASWLL----PNPGKNI----G-    141
NtBBX3     MTTTTTTAIAPDFSK---------VTACLRHHEEDYKFDPWIS----PNTMTTK----L-    177
AtBBX6     ----------STTVD---------LT-AVPVMADDLGLCPWLL----PNDFNEP----AK    160
NtBBX4     -----------YF-D---------SHENNPQPEEEAEAASWIL----PTPKEG-------    145
NtBBX6     -------------------------NPPPPETEEEAEAESWLL----QAPNNNA------    140
AtBBX4     -------------------------------EDGGDVTASWLL----AKEGIEI------    133
AtBBX5     ----------CYFSD---------IDGNGSREEEEEEAASWLL----LPNPKTT----TT    147
NtBBX46    MSIKDNRPRDGQGPQGKENSQNMRAAVEVNDMD-ISEKSNLLVESSTPALDNKQ----HN    181
NtBBX48    MSITDNQPRDNEDPQGKYNSQDMSAAVEMSELHTSSEKSTTLIESSLPALDNKQ----NK    175
NtBBX11    MSITDCRPGDSRGSQAKDKSQDMSVAVEANDLMG----------SLMLSLDSKL----HN    172
AtBBX7     MNIDDDGPTDKKTCNEDKKDV----------LVGSSS---------IPETSS--------    166
AtBBX8     MTIDEDGTGEKSGVQKINV-----------------E---------QPETSS--------    156
AtBBX9     ---------------DTLL------QE-----LDD-----WN--GSSTSVVTQT----QN    143
AtBBX10    ---------------GSFP------LND----LNNTMFDTAY--SMVPHNISYT----QN    153
NtBBX49    SSFDTTSSLSVNENNCSIV------ASKLNELASSMKFEPWA--I--PSNSTYL----TT    167
NtBBX52    LSINENSSLETKGNGGSLV------ANKLNELASNYKFDPWA--I--PSNLNCL----TS    170
AtBBX11    ---DSGLLWE---SP---------EIVSLNDLI-------------VSGGSG-T----HN    146
NtBBX9     YGFSDWLVWD---TP---------SVVSLDDLI-------------APNDS--G----HN    171
NtBBX44    MCKD---VIP---PP----------SVLLQDLM-------------VPNANN-N----SG    163
AtBBX12    MGLD---SWG---SG----------SNIVQELI-------------VPYDVS--------    157
AtBBX13    MQLD---SWV---LG----------SN---ELI-------------VPSDTT--------    156
```

```
AtBBX17     ------------------------------------KQFNFEEEADTMEDLYYNGEEE--     178
AtBBX16     VNNT-------TTNGELGEVEK-AIMDDEGFMGFVPLDMDLEDLTMDVESLLEEEQLC--     213
AtBBX14     ---------MMPLSFKSSDEED-DDNAESCLNGLFPTDMELAQFTADVETLLGGGDR---     196
AtBBX15     ---------MIRSGLMI--EEE-EDNAESCLNGFFPTDMELEEFAADVETLLGRGLDT--     202
NtBBX16     I--NDLDILL-----NSEDVCD-DL----GIPEFLSSEIELAEFAADVESLLGEEFRV--     211
NtBBX14     ATETDPEFKLESKEMQKHDICN-DDLNRFHGM--LPSEMELAEFAADVESLLGKGLED--     228
NtBBX12     -----------SKDQMKNDFND-DHMSKFNGMLNIPSEMELAEFAADVESLLGRRLDD--     193
OsHd1       NNNNN--------NDNDNND---NNNSNSSNNGMYFG--EVDEYF----DLVGYNSY---     194
SlCOL3      KNNVN--------N---------N-NNQTNNYDMLFGGEVVDDYL----DLAEYGGD---     213
StCOL2      KNNVH--------N---------N-NNQTNIYGMLFAGEVVDDYL----DLAEYGGD---     211
NtCOL2b     KNINN--------N-----------NNNQSNYGMLFGGEVVDEYL----DLAEYGGD---     204
NsCOL2      KNINN--------N-----------NNNQSNYGMLFGGEVVDEYL----DLAEYGGD---     204
NtCOL2a     KNINN--------N-----------NNNQNNYGMLFGGEVVDEYL----DLAEYGGD---     204
NtomCOL2    KNINN--------N-----------NNNQNNYGMLFGGEVVDEYL----DLAEYGGD---     204
SlCOL1      ---------------------------KNNNYGMLFGGEVVDDYL----DLAEYGGD---     195
StCO        KNNIN--------N---------NNNNQNNNYGMLFGGEVVDEYL----DLAEYGGD---     212
StCOL1      KNNIN--------N---------NNNNQNNNYGMLFGGEVVDEYL----DLAEYGGD---     212
AtBBX2      ----------------------------N-NNGFSIG----DEFL----NLVDYSSS---     172
AtCO        ----------------------------NQNNGLLFS----DEYL----NLVDYNSS---     185
AtBBX3      ----------------------------NQNNGFLFG----VEYL----DLVDYSSS---     162
NtBBX3      ------------------PV----NTMEMKPMDFLFS--DSENIL----DFD-YSVS---     205
AtBBX6      I-----------------EI---GTENMKGSSDFMFS--DFDRLI----DFE-FPN----     189
NtBBX4      ------------------------TDNQYKSADYLFN--DMDSYL----DMD-IMSC---     171
NtBBX6      ------------------------QGIEYKSAEYLFS--DVDPYV----EMD-MITD---     166
AtBBX4      ----------------------------------------TNLFS----DLD-YPKI---     145
AtBBX5      ATAGIVA----VTSAEEVPG---DSPEMNTGQQYLFS--DPDPYL----DLD-YGNV---     190
NtBBX46     VE----S----PIR-FSNSTMSKGGYMGAKGSSLFEE----DPYC----DNLIM------     218
NtBBX48     AE----L----YAG-SKNYSSSKGCYSGMKGSTIYED----DPFA----QDFNM------     212
NtBBX11     VE----P----PVG-STNLTWSKVSNSATKGSNIFDD----DRFY----DDFNM------     209
AtBBX7      ------V----P----------Q-GKSSSAKDVGMCE----DDFY----GNLGM------     191
AtBBX8      ------A----AQG-MDHSS-VP-ENSSMAKELGVCE----DDFN----GNLIS------     189
AtBBX9      LK-DYSS----FFP-MESN-LP-KV-----IEEECSG----LDLC----EGINL------     176
AtBBX10     FS-DNLS----FFS-TESKGYPDMV-----LKLEEGE----EDLC----EGLNL------     188
NtBBX49     YNIDQAP----FFS--EGSSLPTQSCTTIKDHGIYGG----DDLA----EGVDL------     207
NtBBX52     YKRDPVP----F-S--EGSGLSKQDC-PIKDLGFQEG----DDLA----KGVNF------     208
AtBBX11     FRATDVP----PLPKNRHATCGKYKDEMIRQLRGLSR----SEPG----CLKFETPDA--     192
NtBBX9      FQAIGVP----PLPKNRNAACGKHKEEILCQLRELSK----LEPN----SSDD----Q--     213
NtBBX44     IYSTT-R----EVGRKQNPTCGKQKQVILKQLIELFK----RDFA----DGVGGGSEDLV     210
AtBBX12     ------C----KKQS---FSFGRSKQVVFEQLE-LLK----RGFV----EGEGEIMVPE-     194
AtBBX13     ------F----KKRGSCGSSCGRYKQVLCKQLEELLK----SGVV----GGDGDDGDRDR     198
```

```
AtBBX17      -------------DK------------TDG------AEAC--------------------      187
AtBBX16      -------------LGFK--EPNDV-GVIKEENKVGFEINCKDLKRVKDEDEEEEEA----      253
AtBBX14      -------------EF-H--SIEEL-GL--G----------EMLKIEKEEVEEEGVV----      223
AtBBX15      -------------ES-Y--AMEEL-GLSNS----------EMFKIEKDEIEEEVEEIKA-      234
NtBBX16      -------------VAEE--EEQ-S-RLIN----------GSVEDNKAIKIEVEDEEMRA-      243
NtBBX14      -------------ES-F--DMEGL-GLLGGCNKEENSMECSMVSNEKVKI--EDEVVMD-      268
NtBBX12      -------------EESF--NYMEL-G-LGFLEKHDDSMGCSLVDE-KIKV--ENEGEME-      232
OsHd1        -----------YDNRIEN-NQDRQYGMHEQ---QEQQQQQQEMQKEFAEKEGSECVVPS-      238
SlCOL3       -------------SQFND-QY----NVNQQ---QQ---QYFVPQM----SYGGDSVVPV-      244
StCOL2       -------------SQFND-QY----NVNQQ---QQ---HYSVPQK----SYGGDSVVPV-      242
NtCOL2b      -------------SQFND-QY----SVNQQ---QQ---HYSVPQK----NYGGDSVVPV-      235
NsCOL2       -------------SQFND-QY----SVNQQ---QQ---HYSVPQK----NYGGDSVVPV-      235
NtCOL2a      -------------SQFND-QY----SVNQQ---QQ---NYSVPQK----NYGGDSVVPV-      235
NtomCOL2     -------------SQFND-QY----SVNQQ---QQ---NYSVPQK----NYGGDSVVPV-      235
SlCOL1       -------------SQFND-QY----SVNQQ---QQ---HYSVPQK----SYVEDSVVPV-      226
StCO         -------------SQFND-QY----SVNQQ---QQ---HYSVPQK----SYVEDSVVPV-      243
StCOL1       -------------SQFND-QY----SVNQQ---QQ---HYSVPQK----SYVEDSVVPV-      243
AtBBX2       ------------DKQFTD-QS------NQY---QL---DCNVPQR----SYGEDGVVPL-      202
AtCO         -----------MDYKFTG-EY------SQH---QQ---NCSVPQT----SYGGDRVVPL-      216
AtBBX3       -----------MDNQFED-NQ------YT------------HYQR----SFGGDGVVPL-      187
NtBBX3       --------------------ID---TNSQ-------------PNY----NSANDSVVPV-      224
AtBBX6       -------------------------SFNH-------------HQN----NAGGDSLVPV-      206
NtBBX4       --------------------DQKPHNILQ-------------LHQ----QYSSDGVVPV-      193
NtBBX6       --------------------QKPCTDIQL-------------HVQ----EYKDDCVVPH-      188
AtBBX4       --------------------EV---TSE---------------EN----SSGNDGVVPV-      162
AtBBX5       --------------------DPKVESLE---------------QN----SSGTDGVVPV-      210
NtBBX46      ---------DAVDLSIE--NYEEL-FGASL---N-------YPDE-LFENENFDGLFGM-      254
NtBBX48      ---------DEVDLSFE--NYEEL-FSGSL---D-------NPNQ-FFENEDINGLFGT-      248
NtBBX11      ---------DEVDLSIE--TYEEL-FGVSL---D-------NPDQ-LFKNEDIDGLFGM-      245
AtBBX7       ---------DEVDMALE--NYEEL-FGTAF---N-------PSEE-LFGHGGIDSLFHK-      227
AtBBX8       ---------DEVDLALE--NYEEL-FGSAF---N-------SSRY-LFEHGGIGSLFEK-      225
AtBBX9       ---------DDAPLNFNASNDII--GCSSL---D-------NT---K-------CYEYE-      204
AtBBX10      ---------DDAPLNFDVGDDII--GCSSE---V-------HI---EPDHTVPNCLLID-      223
NtBBX49      ---------DELSSD--C-SYNIF-SSLQQ---S-------HSRY-YSEDRGLDCLIME-      242
NtBBX52      ---------DDVTLSFDCGNYELL-GSSQQ---S-------HPIY-SSEDKELDCLVME-      246
AtBBX11      --------------------EIDA-GF-QF---L-------APD--LFSTCELESGL---      215
NtBBX9       --------------------DDIV-AF-QF---M-------EPA--QNRQLGFK-GS---      235
NtBBX44      PKTPNGSSDWQGNVNVTEGSDAVM-GG-AN---Q-------QLE---PQSVPFTSLLMMQ      255
AtBBX12      -----GIN-G---------------GG-SI---S-------QPS--PTT--SFTS---LL      215
AtBBX13      DCDREGACDGDGD--------GEA-GE-GL---M-------VPE--MSERLKWSRDVEEI      236
```

```
AtBBX17     --------------------------------------PGQYLMSCKKDYDNVITVSEKT   209
AtBBX16     ------------------------------------------------KCE---------   256
AtBBX14     ----------------TREV--HDQDE----------GDETSPFEISFDYEYTHKTTFDE   255
AtBBX15     ---------------MSMDIFDDDRKD----------VDGTVPFELSFDYESSHKTSE--   267
NtBBX16     ---------------VVACHLDPEL------------DMAREALNWDFEYEETVEQKV--   274
NtBBX14     ---------------TTT--TSHDI------------DINGDTFEFKFDYDSPINING--   297
NtBBX12     ---------------VIIDRSSYNQ------------VDYSDTFDLKLDCDSPIITLE--   263
OsHd1       ------------QITMLSE------------------QQHSGYGVVGADQAASM-TAG--   265
SlCOL3      ------------QDGQGKPLIFYQQQQ-------QQQQSHHQNFQLGMEYDNSN-TRL--   282
StCOL2      ------------QDGQGKSLFFYHHQ----------QQSHHLNFQLGMDYDNSY-TRL--   277
NtCOL2b     ------------QGGHGKSMILYHQQQQQHH--QQQQQNHHLSFQLGMEYDNSN-TGY--   278
NsCOL2      ------------QGGHGKSMILYHQQQQQHH--QQQQQNHHLSFQLGMEYDNSN-TGY--   278
NtCOL2a     ------------QDRQGKSMILYQQQQQ-----QQQQHNHHLSFQLGMEYDNSN-TGY--   275
NtomCOL2    ------------QDRQGKSMILYQQQQQ-----QQQQHNHHLSFQLGMEYDNSN-TGY--   275
SlCOL1      ------------QNGQRKSLILYQTPQQ--------QQSHHLNFQLGMEYDNSN-TGY--   263
StCO        ------------QNGQRKSLILYHQPQQQQ---QQQQQSHHLNFQLGMEYDNSN-TGY--   285
StCOL1      ------------QNGQRKSLILYHQPQQQQ---QQQQQSHHLNFQLGMEYDNSN-TGY--   285
AtBBX2      ------------QIEVSKGMY-----------------QEQQNFQLSINCGSWGALRS--   231
AtCO        ------------KLEESRGHQ----------------CHNQQNFQFNIKYGSS-GTHY--   245
AtBBX3      ------------QVEESTSHL----------------QQSQQNFQLGINYGFSSGAHY--   217
NtBBX3      ------------QSTIKPL----------------P--FQHQEKHFEIDFTQSH---I--   249
AtBBX6      ------------QTKTEPL----------------P--LTNNDHCFDIDFCRS-KLSA--   233
NtBBX4      ------------QKKNENNHLQG------------P--VVDGFPTYEMDFIGS-KPYL--   224
NtBBX6      ------------VQNKNEIHLQG------------P--VVNGYPTYEMDFSGS-KPFM--   219
AtBBX4      ------------QNKLF----------------------L-NEDYFNFDLSASK---I--   182
AtBBX5      ------------ENRTVRI----------------P--TV-NENCFEMDFTGGSKGFT--   237
NtBBX46     K--DI--------------------------------KSADSNCRGANAAEGSSIARV--   278
NtBBX48     K--DM--------------------------------SVSGSSCQDADAIEGSSIRRV--   272
NtBBX11     K--DM--------------------------------AVAESSCQGDTAVEGSALGRV--   269
AtBBX7      H--QT--------------------------------APE--------------------   233
AtBBX8      D--EA--------------------------------HEG--------------------   231
AtBBX9      D------------------------------------SF------------------K--   208
AtBBX10     KTNTS--------------------------------SFTGSNFTVDKALEASPPGQQ--   249
NtBBX49     K--NL--------------------------------SVIGPNSHVETALEAPCS-----   263
NtBBX52     K--NS--------------------------------SVTGSNSHVETSLEATSSGPQ--   270
AtBBX11     --------K-WFD--------QQDHEDFPYCSLLKNLSESDEKP----------------   242
NtBBX9      --------G-FMQNSEHAVVPSSEGSAFNWCSDTGKFTDQGFSSSLADCFIETKC-----   281
NtBBX44     KPHNSKDSDRMVQGNILSSGNSYVQNTQIWDFNLGQLRSHEQSSSVEADYSESDMA----   311
AtBBX12     M--SQSLCGNGMQWNAT--NHSTGQNTQIWDFNLGQSRNPDEPSPVETK------GST--   263
AtBBX13     N--GGGGGGVNQQWNATTTNPSGGQSSQIWDFNLGQSRGPEDTSRVEAAYVGKGAASS--   292
```

```
AtBBX17     -------------------------------EEIEDCYENNARHRLNYENVIAAWDKQES     238
AtBBX16     -----------NGGSK--DSDR--------EASNDKDRKTSLFLRLDYGAVISAWDNHGS     295
AtBBX14     GEEDEKEDVMKNVMEMGVNEMS--------GGIKEEKKEKALMLRLDYESVISTWGGQGI     307
AtBBX15     ------EEVMKNVESSG--ECV--------VKVKEEEHKNVLMLRLNYDSVISTWGGQGP     311
NtBBX16     -NMTAAT----EFVPS----AE--------CGGSKADAKRRLLLRLNYEAVISAWPNQSS     317
NtBBX14     -EDEVGEVVMKINGED----GV--------VGDGNYNEKKKILLNLDYESVLTAWADQRS     344
NtBBX12     -EDDKGD-----------------------------TKNNKILLNLDYGGVLNAWADQRS     293
OsHd1       -------V--SAYTDS----------------ISNS------------------------     276
SlCOL3      -------G----YPAS----------------MSHS------------------------     291
StCOL2      -------G----YPAS----------------MSHS------------------------     286
NtCOL2b     -------G----YPAS----------------MSHS------------------------     287
NsCOL2      -------G----YPAS----------------MSHS------------------------     287
NtCOL2a     -------G----YPAS----------------MSHS------------------------     284
NtomCOL2    -------G----YPAS----------------MSHS------------------------     284
SlCOL1      -------G----YPAS----------------LSHS------------------------     272
StCO        -------G----YPAS----------------LSHS------------------------     294
StCOL1      -------G----YPAS----------------LSHS------------------------     294
AtBBX2      ----------SNG--S----------------LSHM------------------------     239
AtCO        -------N--DNG--S----------------INHN------------------------     254
AtBBX3      -------N--NNSLKD----------------LNHS------------------------     228
NtBBX3      -------K--SYTTPS----------------LSHS------------------------     260
AtBBX6      -------F--TYPSQS----------------VSHS------------------------     244
NtBBX4      -------Y--NFNSQS----------------ISQS------------------------     235
NtBBX6      -------Y--NFSSQS----------------ISQS------------------------     230
AtBBX4      -------S--QQGFNF----------------INQT------------------------     193
AtBBX5      -------Y--GGGYNC----------------ISHS------------------------     248
NtBBX46     -------NTVQPTCS----------------------------NAESADSAMSCKTDSIL     303
NtBBX48     -------MTMQPACS----------------------------NAESADSLVSCKTEPSI     297
NtBBX11     -------NTVQPACS----------------------------NAASAESTMSCKTEPTL     294
AtBBX7      -------G--GNSVQ----------------------------PAGSNDSFMSSKTEPII     256
AtBBX8      -------SMQQPALS----------------------------NNASADSFMTCRTEPII     256
AtBBX9      -------EENN-----------------------------------------IGLPSLLL     220
AtBBX10     -------MNIN-----------------------------------------TGLQLPLS     261
NtBBX49     ----------------------------------------VSGTANSMFMNPSCNGDTVL     283
NtBBX52     -------EYMGLQASQMAAAASST----------NL-FQTMSATANCMLMNPSSIGL---     309
AtBBX11     ---------------------ENVDR-------------------ESSVMVPVSGC----     258
NtBBX9      ---------------------LLPDR-------------------DSDVGDA-SGG----     296
NtBBX44     -------YMMKSYGELIKGTSLASSKGLELSGINRS-VAHEDMTALSNNSNNRGGS----     359
AtBBX12     -------FTFNNVTHLKNDTRTTNMNA-----FKES-Y-------QQEDSVHSTST----     299
AtBBX13     -------FTINNFVDHMNETCSTNVKG-----VKEI-K-------KDDYKRSTSGQ----     328
```

```
AtBBX17      PRDVKNNTS--------SFQLV-------------------------PP----------      254
AtBBX16      PWKTGIKPEC-MLGGNTCLPHVVGGYE-KLMSSDGSVTR---------QQ----------      334
AtBBX14      PWTARVPSEI-DLDM-VCFPTHTMGE----SG-AEAHHH---------NHFRG------L      345
AtBBX15      PWSSGEPPER-DMDI-SGWPAFSMVE----NG-GESTHQ---------KQYVG------G      349
NtBBX16      PWTNGIRPHF-NPD--DCWPDFLETC----IG-EGGVHQ---------PYGGG------K      354
NtBBX14      PWTNGERPEL-DSN--DCWPD----C----MG-NCGTIH---------PYGI--------      375
NtBBX12      PWTTGERPEV-DFN--DCWPV----C----MG-NCGKNH---------LYGDM------G      326
OsHd1        -------ISFSSME--AGIVPDSTVI--DM-----PNSRILTPAGAINLF-SGP-S---L      315
SlCOL3       -------VSVVSMD--VSVVPESALC--ET-----SNSQPRPQKGTIELF-SGH-P---I      330
StCOL2       -------VSVSSMD--VSVVPESALS--ET-----SNSHSRPQKGTIDLF-SGP-P---I      325
NtCOL2b      -------VSMSSMD--VSVVPESALS--ET-----SNSHPRPPKGTIDLF-SGP-P---I      326
NsCOL2       -------VSMSSMD--VSVVPESALS--ET-----SNSHPRPPKGTIDLF-SGP-P---I      326
NtCOL2a      -------VSISSID--VSVVPESALS--ET-----SNSHPRLPKGTIDLF-SGP-P---I      323
NtomCOL2     -------VSISSID--VSVVPESALS--ET-----SNSHPRLPKGTIDLF-SGP-P---I      323
SlCOL1       -------VSISSMD--VSVVPESAQS--ET-----SNSHPRPPKGTIDLF-SGP-P---I      311
StCO         -------VSISSMD--VSVVPESALS--ET-----SNSHPRPPKGTIDLF-SGP-P---I      333
StCOL1       -------VSISSMD--VSVVPESALS--ET-----SNSHPRPPKGTIDLF-SGP-P---I      333
AtBBX2       -------VNVSSMD--LGVVPESTTS--DA-----TVSNPRSPKAVTDQP-PYP-P---A      278
AtCO         -------AYISSME--TGVVPESTAC--VT-----TASHPRTPKGTVEQQ-PDP-A---S      293
AtBBX3       -------ASVSSMD--ISVVPESTAS--DI-----TVQHPRTTKETIDQL-SGP-P---T      267
NtBBX3       -------VSSSSLD--VGIVPDGSSIS-EI-----SYPFVRSVNSIIEMGSSAP------      299
AtBBX6       -------VSTSSIE--YGVVPDGNTNN-SV-----NRSTITSST-------TGG------      276
NtBBX4       -------VSSSSMD--VGVVPEHSAMA-DV-----SNTLVMNSS----AD-AGV------      269
NtBBX6       -------VSSSSME--VGVVPDHNTMA-DV-----SNTFVRNSS-----T-DGL------      263
AtBBX4       -------VSTRTID--VPLVPESGGVT-AE-----M-----------TNT-ETP------      220
AtBBX5       -------VSSSSME--VGVVPDGGSVA-DV-----SYPYGGPATSGADPG-TQR------      286
NtBBX46      YFARQ-----S--S--LSFSNQTGEST-AGDHQDCGVSPML-LMGEPPPWGPP------C      346
NtBBX48      CFARQ--A--S--I--LSFSNLGGESN-AGDYQECGASTML-LMGEP-PWYHP------F      340
NtBBX11      CFARQ--Q--S--N--LSVSNLTGESS-AGDYQDYGASSML-LMGEP-PWCSP------C      337
AtBBX7       CFASKPAH--S--N--ISFSGVTGESS-AGDFQECGASSSIQLSGEP-PWYPP------T      302
AtBBX8       CYSSKPAH--S--N--ISFSGITGESN-AGDFQDCGASSMKQLSREPQPWCHP------T      303
AtBBX9       PTLSGNVV--PNMS--LSMSNLTGESN-ATDYQDCGISPGF-LIGDS-PWESN------V      267
AtBBX10      PVLFGQIH--PS-------LNITGENN-AADYQDCGMSPGF-IMSEA-PWETN------F      303
NtBBX49      AFTQGPVH---SRS--LSISNITTESSEATDYQDCGFSPLF-PPC---DWNSE------T      328
NtBBX52      PFLPAPIH--SSMS--LSLSNITGESSATTDYQDCGLSPVF-LNGE--SWDLN------L      356
AtBBX11      ---LNRCEEETVMV--PVIT---------------------------------------S      274
NtBBX9       ------ANEEQSHN--PPIAD-----------------------------------TFQMV      314
NtBBX44      ---QGPATSE-SNN--LPIIKLSSDSG-YTKRKCCGVSKDLNFMEQS-IFVGGE-NTGEE      410
AtBBX12      ---KGQETSK-SNN--IPAAIHSHKSS--------NDSCGLHCTEHI-AITSNRATRLVA      344
AtBBX13      ---VQPTKSE-SNN--RPITFGSEKGS--------NSSSDLHFTEHI-AGTSCKTTRLVA      373
```

```
                                CCT domain           NLS
AtBBX17       --GIEEKRVRSEREARVWRYRDKRKNRLFEKKIRYEVRKVNADKRPRMKGRFVRRSLAID    312
AtBBX16       --GRDGGGSDGEREARVLRYKEKRRTRLFSKKIRYEVRKLNAEQRPRIKGRFVKRTSLLT    392
AtBBX14       -GLHLGDAGDGGREARVSRYREKRRTRLFSKKIRYEVRKLNAEKRPRMKGRFVKRSSIGV    404
AtBBX15       -CLPSSGFGDGGREARVSRYREKRRTRLFSKKIRYEVRKLNAEKRPRMKGRFVKRASLAA    408
NtBBX16       -----VRGGDGGREERVSRYREKRRTRLFSKKIRYQVRKLNAEKRPRLKGRFVKRTTSFS    409
NtBBX14       -MNGHTTMIDRGREARVSRYREKRRTRLFSKTIRYEVRKLNAEKRPRMKGRFVKRSN-LV    433
NtBBX12       TMTGHGPTVDEGREARVLRYKEKRRTRLFSKKIRYEVRKLNAEKRPRMKGRFVKRTN-FA    385
OsHd1         QM--SLHFSSMDREARVLRYREKKKARKFEKTIRYETRKAYAEARPRIKGRFAKRSDVQI    373
SlCOL3        QI--P-LLTPMDREARVLRYREKKKNRKFEKTIRYASRKAYAETRPRIKGRFAKRTDVEA    387
StCOL2        QI--PPQLTPMDREARVLRYREKKKNRKFEKTIRYASRKAYAETRPRIKGRFAKRTDVEA    383
NtCOL2b       QM--PTQLTPMDREARVLRYREKKKNRKFEKTIRYASRKAYAETRPRIKGRFAKRTDVEA    384
NsCOL2        QM--PTQLTPMDREARVLRYREKKKNRKFEKTIRYASRKAYAETRPRIKGRFAKRTDVEA    384
NtCOL2a       QM--PTQLTPMDREARVLRYREKKRNRKFEKTIRYASRKAYAETRPRIKGRFAKRTDVEA    381
NtomCOL2      QM--PTQLTPMDREARVLRYREKKKNRKFEKTIRYASRKAYAETRPRIKGRFAKRTDVEA    381
SlCOL1        QI--PPQLTPMDREARVLRYREKKKNRKFEKTIRYASRKAYAETRPRIKGRFAKRTDVEA    369
StCO          QI--PPQLTPMDREARVLRYREKKKNRKFEKTIRYASRKAYAETRPRIKGRFAKRTDVKA    391
StCOL1        QI--PPQLTPMDREARVLRYREKKKNRKFEKTIRYASRKAYAETRPRIKGRFAKRTDVKA    391
AtBBX2        QM-----LSPRDREARVLRYREKKKMRKFEKTIRYASRKAYAEKRPRIKGRFAKKKDVDE    333
AtCO          QMITVTQLSPMDREARVLRYREKRKTRKFEKTIRYASRKAYAEIRPRVNGRFAKREIEA-    352
AtBBX3        QV--VQQLTPMEREARVLRYREKKKTRKFDKTIRYASRKAYAEIRPRIKGRFAKRIETEA    325
NtBBX3        ----AEKLIGMNREARVLRYREKKKNRKFEKTIRYASRKAYAETRPRIKGRFAKRNDSDG    355
AtBBX6        ----DHQASSMDREARVLRYREKRKNRKFEKTIRYASRKAYAESRPRIKGRFAKRTETEN    332
NtBBX4        ----PNPVSGLDREARVLRYREKRKNRKFEKTIRYASRKAYAETRPRIKGRFAKRTEIEV    325
NtBBX6        ----PNPLSNLDREARVLRYREKRKNRKFEKTIRYASRKAYAETRPRIKGRFAKRTENEV    319
AtBBX4        ----AVQLSPAEREARVLRYREKRKNRKFEKTIRYASRKAYAEMRPRIKGRFAKRTDSRE    276
AtBBX5        ----AVPLTSAEREARVMRYREKRKNRKFEKTIRYASRKAYAEMRPRIKGRFAKRTDTNE    342
NtBBX46       PEP---SSSSTSRSNAVLRYKEKKKTRKFDKRVRYVSRKARADVRRRVKGRFVKAGDAYD    403
NtBBX48       PET---SLPSTSRSNAVLRYKEKKKIRKFDKHVRYASRKARADVRRRVKGRFVKAGDAYD    397
NtBBX11       PES---SMPSTSRSDAVLRYKEKKKTRQFDKRVRYASRKARADVRRRVKGRFVKAGDAYD    394
AtBBX7        LQDNNACSHSVTRNNAVMRYKEKKKARKFDKRVRYASRKARADVRRRVKGRFVKAGEAYD    362
AtBBX8        AQDIIASSHATTRNNAVMRYKEKKKARKFDKRVRYVSRKERADVRRRVKGRFVKSGEAYD    363
AtBBX9        EV----SFNPKLRDEAKKRYKQKKSKRMFGKQIRYASRKARADTRKRVKGRFVKSGETFE    323
AtBBX10       EV----SC-PQARNEAKLRYKEKKLKRSFGKQIRYASRKARADTRKRVKGRFVKAGDSYD    358
NtBBX49       -S------CVRARNEAKMRYNEKKKTRTFRKQIRYASRKVRADTRRRVKGRFVKAGEAYD    381
NtBBX52       EK------SPQKRHEAKMRYNEKKKSRTFGKQIRYESRKARADTRRRVKGRFVKAGEAYD    410
AtBBX11       TRSMTHEINSLERNSALSRYKEKKKSRRYEKHIRYESRKVRAESRTRIRGRFAKAADP--    332
NtBBX9        PKVAHRELNSQERETAVSRYKEKKKTRRYEKHIRYVSRKVRAESRTRIKGRFAKMDMHQ-    373
NtBBX44       ILKADMELLAKNRGNAMQRYKEKKKTRRYDKHIRYESRKARADTRKRVKGRFVKANEAPD    470
AtBBX12       VTNADLEQMAQNRDNAMQRYKEKKKTRRYDKTIRYETRKARAETRLRVKGRFVKATDP--    402
AtBBX13       -TKADLERLAQNRGDAMQRYKEKRKTRRYDKTIRYESRKARADTRLRVRGRFVKASEAPY    432
                          *     **.:*:  * : * :**  **  *: * *:.***.:
```

```
AtBBX17       ------S-----------------------   313
AtBBX16       ------------------------------   392
AtBBX14       -------AH---------------------   406
AtBBX15       -------AASPLGVNY--------------   417
NtBBX16       ------PPGFPYLMNKR-------------   420
NtBBX14       ------PN-YPLLK----------------   440
NtBBX12       ------PTPFPSLNR---------------   394
OsHd1         -----EVDQMFSTAA---LSDGSYGTVPWF   395
SlCOL3        -----EVDQMFSTQL---MTDSSYRIVPSF   409
StCOL2        -----EVYQMFSTQL---MADSSYRIVPSF   405
NtCOL2b       -----EVDQMFSTQL---IADSSYGIVPSF   406
NsCOL2        -----EVDQMFSTQL---IADSSYGIVPSF   406
NtCOL2a       -----EVDQMFSTQL---IADSSYGIVPSF   403
NtomCOL2      -----EVDQMFSTQL---IADSSYGIVPSF   403
SlCOL1        -----EVDQMFSTQL---MTDSNYGIVPSF   391
StCO          -----EVDQMFSTQL---MTDSSYGIVPSF   413
StCOL1        -----EVDQMFSTQL---MTDSSYGIVPSF   413
AtBBX2        -----EANQAFSTMI---TFDTGYGIVPSF   355
AtCO          ------EEQGFNTML---MYNTGYGIVPSF   373
AtBBX3        -----EAEEIFSTSL---MSETGYGIVPSF   347
NtBBX3        AGVDSDIDQIFSGAGFI-ANDSRYGVVLSF   384
AtBBX6        -------DDIFLSHVYASAAHAQYGVVPTF   355
NtBBX4        -------DSLLA-------ADASYGVVPSF   341
NtBBX6        -------DSLIAS------SDASYGVVPSF   336
AtBBX4        -------ND--GGDV---GVYGGFGVVPSF   294
AtBBX5        -------SNDVVGHG---GIFSGFGLVPTF   362
NtBBX46       ------YDPLPTR-SY--------------   412
NtBBX48       ------YDPLNETRSF--------------   407
NtBBX11       ------YDPLSETRSY--------------   404
AtBBX7        ------YDPLTPTRSY--------------   372
AtBBX8        ------YDPMSPTRSY--------------   373
AtBBX9        ------YDPSLVM-----------------   330
AtBBX10       ------YDPSSPTTNN--------------   368
NtBBX49       ------FDPLVTRDM---------------   390
NtBBX52       ------YDPSETKEF---------------   419
AtBBX11       ------------------------------   332
NtBBX9        ------------------------------   373
NtBBX44       ------G-----------------------   471
AtBBX12       ------------------------------   402
AtBBX13       ------P-----------------------   433
```

Supplementary Figure S2. Multiple sequence alignment of CO(L)/BBX proteins from *Arabidopsis thaliana* (At), *Oryza sativa* (Os), *Solanum lycopersicum* (Sl), *Solanum tuberosum* (St) and *Nicotiana tabacum* (Nt) generated with Clustal Omega (Madeira et al., 2022) and used for domain analysis (Figure 1 B, C, D). The corresponding accession numbers are listed in Supplementary Table S9 and S11. Conserved domains were identified by comparison with protein sequence databases using the InterProScan online tool (Jones et al., 2014). Annotation of the conserved B-box zinc finger domains and the CCT domain was carried out manually according to Robson et al. (2001). Characteristic cysteine and histidine residues conserved in the B-box domains (Figure 1C) are highlighted in blue (according to Robson et al., 2001), and the putative nuclear localization signal (NLS) in the CCT domain (Figure 1D) is highlighted in yellow (according to Crocco and Botto, 2013). Asterisks indicate identical amino acids in all sequences, colons indicate conserved substitutions and periods indicate semi-conserved substitutions.

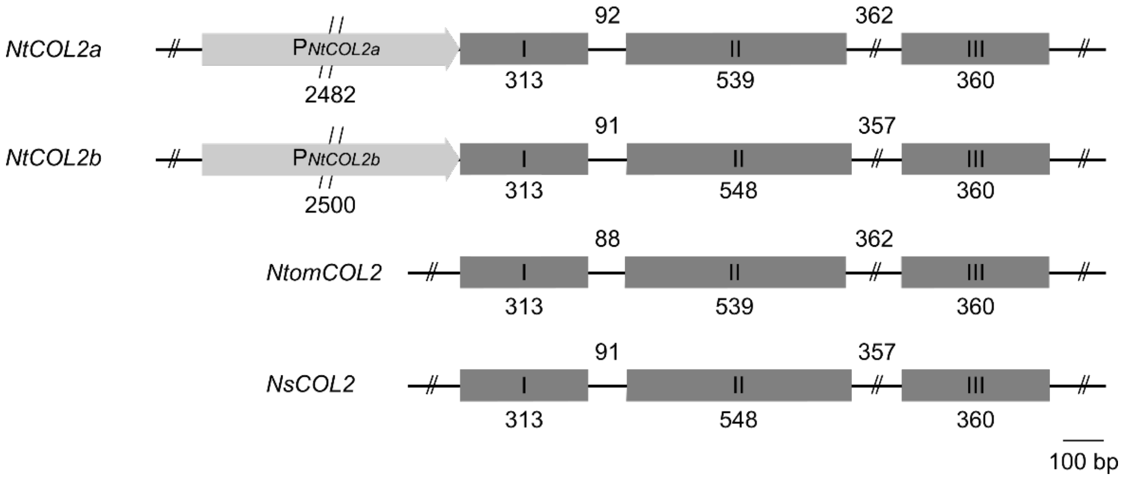

Supplementary Figure S3. Genomic structure of *NtCOL2a, NtCOL2b, NtomCOL2* and *NsCOL2*. Genomic and coding sequences of *NtCOL2a* and *NtCOL2b* were confirmed by isolation from *N. tabacum* cv. SR1 plants. For promoter–reporter studies, the isolated regions included ~2.5 kb of the upstream promoters ($P_{NtCOL2a}$ and $P_{NtCOL2b}$). Alignment with the isolated coding sequences verified the exon structure of the genes in *N. tabacum* cv. SR1, each comprising three exons (I–III). The coding sequence of *NtCOL2a* (1212 bp) was slightly shorter compared to *NtCOL2b* (1221 bp), which nevertheless shared 95.2% identity in a pairwise sequence alignment (Madeira et al., 2019). In the genome of cv. SR1 plants, the exons were separated by two introns with minor differences between *NtCOL2a* and *NtCOL2b*. The *NtCOL2a* and *NtCOL2b* genomic sequences (exons and introns) were 94.2% identical, which indicated the close phylogenetic relationship of the two homologous genes. The isolated promoter sequences showed more variation (33.7% identity in the first 2.4 kb upstream of the genes). The genomic loci (exons and introns) of the ancestral genes *NtomCOL2* and *NsCOL2* were confirmed by isolation from the progenitors. The sequences of *NtomCOL2* and *NsCOL2* showed the same exon structure as the *N. tabacum* homologs. Exons are shown as boxes, introns as lines, and the promoter as an arrow.

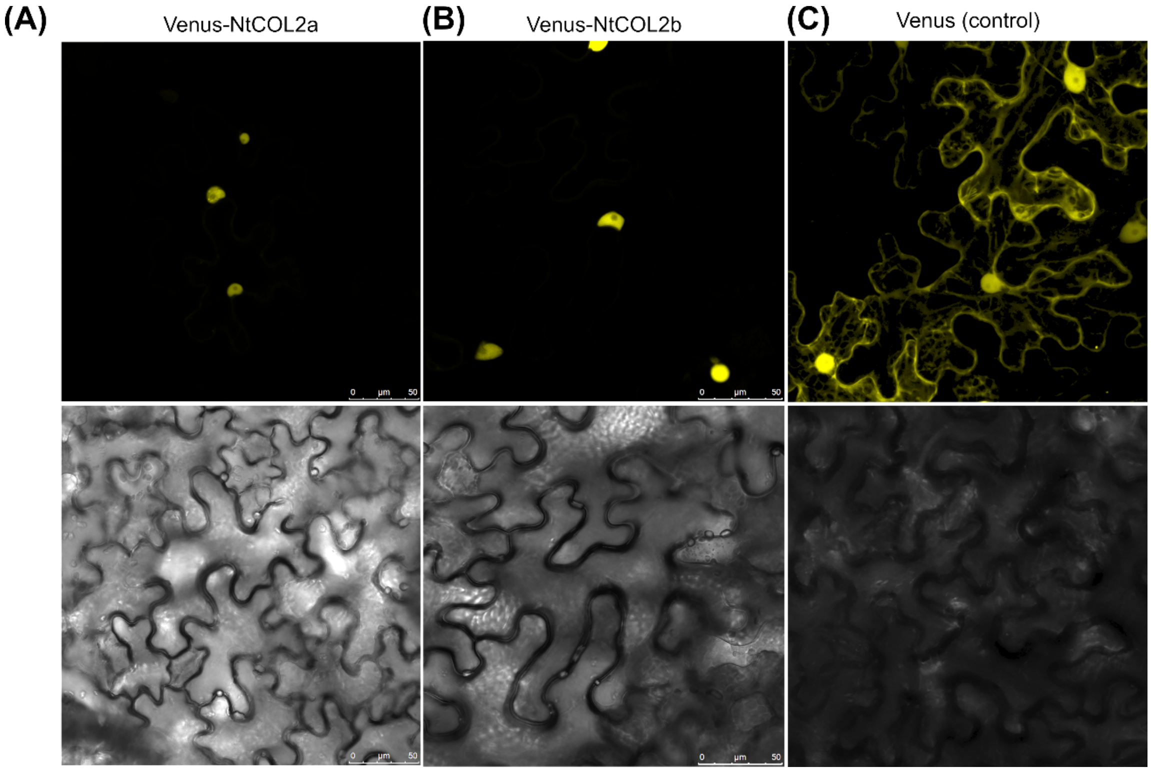

Supplementary Figure S4. Single channel images (brightfield (bottom) and fluorescence (top)) of the overlay pictures from Fig. 1E–G. Subcellular localization of NtCOL2a and NtCOL2b in *N. benthamiana* leaf epidermis cells revealed by Venus fluorescence in abaxial epidermal cells expressing P_{35S}:*Venus-NtCOL2a* (A), P_{35S}:*Venus-NtCOL2b* (B) and P_{35S}:*Venus* (C) detected by confocal laser scanning microscopy. Venus-NtCOL2a (A) and Venus-NtCOL2b (B) fusion proteins are present in the nucleus. Venus localization (C) was used as a control. The representative cells were from *N. benthamiana* plants cultivated for 3 days under continuous light after transient protein expression. Scale bars = 50 µm.

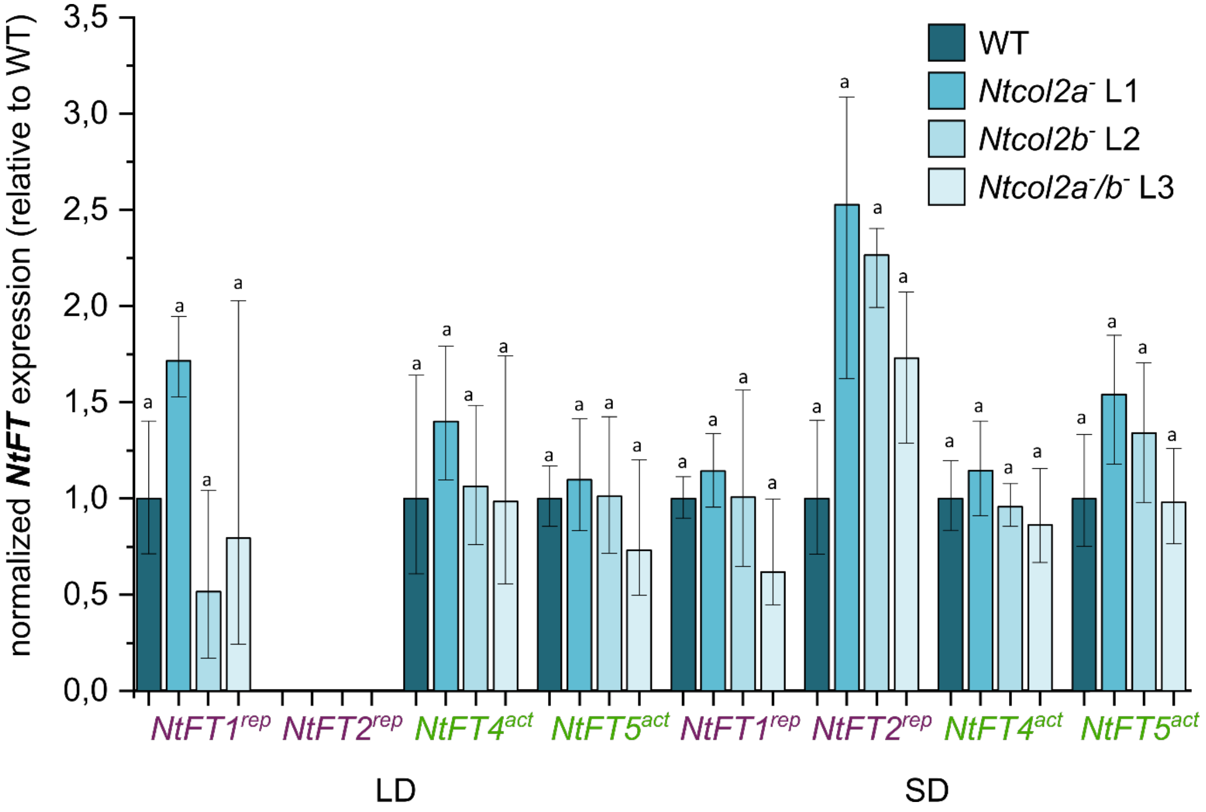

Supplementary Figure S5. Expression levels of *NtFT1*^{rep}, *NtFT2*^{rep}, *NtFT4*^{act} and *NtFT5*^{act} in medial leaves (harvested 4 h after dawn) of nullizygous *Ntcol2a*$^{-}$, *Ntcol2b*$^{-}$ and *Ntcol2a*$^{-}$/*b*$^{-}$ T_2-plants (different shades of light blue) grown under LD and SD conditions, compared to SR1 wild-type plants (dark blue; set to 1). Expression was normalized to the reference gene *NtEF-1α*. Data are means of three biological replicates of medial leaves pooled from three plants for each replicate ± standard errors (SEM). No statistically significant differences between each knock-out line and the WT control were detected by one-way ANOVA and Tukey´s post hoc test ($P < 0.05$).

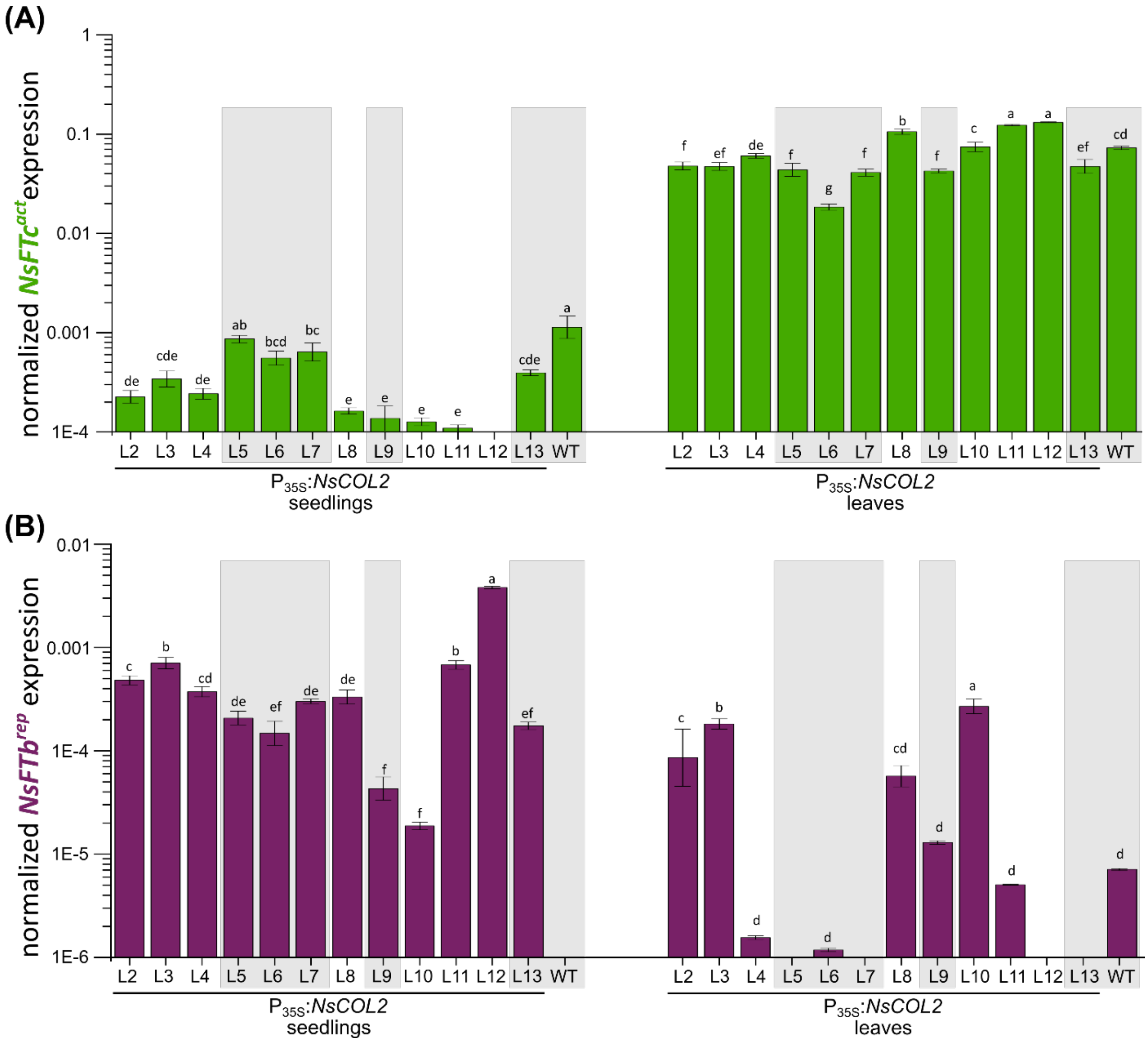

Supplementary Figure S6. Expression levels of *NsFTc*^{act} (A) and *NsFTb*^{rep} (B) in seedlings (left) and leaves (right) of transgenic *NsCOL2* overexpression lines and *N. sylvestris* wild-type (WT) plants cultivated under SD conditions. Non-flowering plant lines are highlighted with a gray box. The same plant lines (and material) is presented in Fig. 7. *NsFTc*^{act} and *NsFTa*^{rep} expression was normalized to the reference gene *NsEF-1α*. Data are means of three technical replicates ± standard errors (SEM) based on log-transformed data. Statistically significant differences between each overexpression line and the WT control (shown using different lower case letters) were determined by one-way ANOVA and Tukey's post hoc test ($P < 0.05$).

Supplementary Materials and Methods

Plant material, growth conditions and harvesting time points

For spatial expression analysis, apical, medial and basal leaves, as well as stems, were separately harvested from vegetative and reproductive wild-type *N. tabacum* plants cultivated under LD or SD conditions (Supplementary Table S1). At dawn, nine plants were sampled and three pools containing three plants each were created to yield three biological replicates per tissue and time point. To analyze the diurnal expression of *NtCOL2a, NtCOL2b, NsCOL2* and *NtomCOL2*, medial leaves were harvested at 4-h intervals from vegetative and reproductive wild-type *N. tabacum*, *N. sylvestris* and *N. tomentosiformis* plants grown under LD and SD conditions (Supplementary Table S1). Reproductive *N. sylvestris* and *N. tomentosiformis* plants were only sampled under LD or SD conditions, respectively. Starting at dawn (zeitgeber time 0 h), material from each species and growth stage (three pools each) was harvested at seven time points (zeitgeber times 0, 4, 8, 12, 16, 20 and 24 h), with each pool containing tissues pooled from three individuals.

Supplementary Table S1: Wild-type tobacco plants sampled for spatial and diurnal expression analysis by quantitative real-time PCR (qPCR). Samples were taken after the indicated periods (weeks after seed sowing, WASS) from vegetative or reproductive plants cultivated under LD or SD conditions.

Analysis	Species	Conditions	Period (WASS)	Growth stage
Spatial / diurnal expression	*N. tabacum*	LD	4.5	vegetative (~7–9 leaves)
			6.5	reproductive (floral buds)
		SD	6	vegetative (~11–12 leaves)
			8	reproductive (floral buds)
Diurnal expression	*N. sylvestris*	LD	6	vegetative (~10–12 leaves)
		LD	8	reproductive (floral buds)
		SD	8	vegetative (~12–13 leaves)
	N. tomentosiformis	LD	7.5	vegetative (~11–13 leaves)
		SD	11.5	vegetative (~11–13 leaves)
		SD	22.5	reproductive (floral buds)

For *NtCOL2a/b* overexpression analysis in *N. tabacum*, leaf tissue was harvested from independent transgenic T_0 plants after callus regeneration under LD conditions. In the T_1 generation, material (whole seedlings or leaves) was harvested and pooled from at least three plants cultivated under LD or SD conditions. For the analysis of *NtFT* expression in *NtCOL2a/b* knockout lines, medial leaves of nullizygous *Ntcol2a*$^{-}$, *Ntcol2b*$^{-}$ and *Ntcol2a*$^{-}$*/b*$^{-}$ plants (T_2 generation) and *N. tabacum* WT at the reproductive stage grown under LD and SD conditions were harvested 4 h after dawn (three pools of medial leaves pooled from three plants for each replicate). For *NsCOL2* overexpression analysis and the analysis of *NsFT* expression in *N. sylvestris*, seedlings (at least three pooled individuals) and leaf tissue from individual P_{35S}:*NtCOL2b* T_1 plants (and *N. sylvestris* WT) cultivated under SD conditions were harvested when the seven transgenic lines started flowering. Leaf tissue from transgenic P_{Q35S}:*NtCOL2a-3xc-myc* or P_{Q35S}:*NtCOL2b-3xc-myc* plants (and VCpBin19 vector control (VC) plants) was used for the immunodetection of the encoded fusion proteins. Samples were taken from vegetative and reproductive T_1 plants cultivated under LD conditions in soil (~3 weeks and ~5 weeks after transfer from sterile MS medium to the greenhouse). Each sample contained material pooled from three individuals. Leaves (one leaf per plant, position ~3–4 counted from the plant tip) of vegetative plants were harvested at six different zeitgeber times during the day, including 1 h before dawn (23 h), 1 h after dawn (1 h), 1 h before the end of the light period (15 h), and 1 h after the end of the light period (17 h). The remaining two samples were taken in the middle of light (8 h) and dark (20 h) periods, respectively. Plants sampled 1 h after the beginning of the light period (1 h) were sampled again when they were reproductive and formed visible floral buds (one leaf per plant, position ~4–5 counted from the plant tip, zeitgeber time 1 h).

Primers and oligonucleotides

The primers and oligonucleotides used in this study are listed in Supplementary Table S2.

Supplementary Table S2: List of primers and oligonucleotides used in this study. Underlines indicate 5′ overhangs for cloning, which may contain restriction sites (in italics) and sequences encoding a protein linker and the 3xc-myc tag (in lowercase letters).

Name	Sequence (5′ to 3′)	Usage (Reference)
**Primers and other oligonucleotides for the cloning of plasmid constructs**		
$P_{NtCOL2a}$ for KpnI<br>$P_{NtCOL2a}$ rev SalI	AGA*GGTACC*TTTGTTCTTGAAGTGGGC<br>AGA*GTCGAC*TGAACTAATTTTGGCTTCTT	Isolation of $P_{NtCOL2a}$, cloning of reporter gene constructs
$P_{NtCOL2b}$ for KpnI<br>$P_{NtCOL2b}$ rev XhoI	AGA*GGTACC*AACTGGACACTTATTTTGAAC<br>AGA*CTCGAG*TGAACTAATTTTTGGCTTATT	Isolation of $P_{NtCOL2b}$, cloning of reporter gene constructs
NtCOL2a/*b* for XhoI<br>*NtCOL2a* rev XbaI	AGA*CTCGAG*ATGTTGAAAAAGGAAAACAGCAGC<br>AGA*TCTAGA*TCAGAATGATGGCACGATTCCATAAC	Cloning of P_{35S}:*Venus-NtCOL2a* construct
NtCOL2b for XhoI<br>*NtCOL2b* rev XbaI	AGA*CTCGAG*ATGTTGAAAAAGGAGAACAGCAGC<br>AGA*TCTAGA*TCAGAATGAAGGGACAATTCCATAAC	Cloning of P_{35S}:*Venus-NtCOL2b* construct
NtCOL2a/*b* for XhoI<br>*NtCOL2a* rev XbaI	AGA*CTCGAG*ATGTTGAAAAAGGAAAACAGCAGC<br>AGA*TCTAGA*TCAGAATGATGGCACGATTCCATAAC	Cloning of P_{35S}:*NtCOL2a* construct
NtCOL2a/*b* for XhoI<br>*NtCOL2b* rev XbaI	AGA*CTCGAG*ATGTTGAAAAAGGAAAACAGCAGC<br>AGA*TCTAGA*TCAGAATGAAGGGACAATTCCATAAC	Cloning of P_{35S}:*NtCOL2b* construct
NtCOL2a/*b* for XhoI<br>*NtCOL2a 3xc-myc* rev XbaI	AGA*CTCGAG*ATGTTGAAAAAGGAAAACAGCAGC<br>ACA*TCTAGA*ttaaagatcctcctcagaaatcaacttttgctcattcaagtcctcttca gaaatgagcttttgttcattcaagtcctcttcagaaatgagcttttgctccatgcttccgcca ccgccagatccaccgcctccGAATGATGGCACGATTCCATAAC	Cloning of P_{Q35S}:*NtCOL2a-3xc-myc* construct
NtCOL2a/*b* for XhoI<br>*NtCOL2b 3xc-myc* rev XbaI	AGA*CTCGAG*ATGTTGAAAAAGGAAAACAGCAGC<br>ACA*TCTAGA*ttaaagatcctcctcagaaatcaacttttgctcattcaaatcctcttca gaaatgagcttttgttcattcaagtcctcttcagaaatgagcttttgctccatgcttccgcca ccgccagatccaccgcctccGAATGAAGGGACAATTCCATAAC	Cloning of P_{Q35S}:*NtCOL2b-3xc-myc* construct
PS *NtCOL2a*$_{\text{exI 71..93 bp}}$ for<br>PS *NtCOL2a*$_{\text{exI 71..93 bp}}$ rev	ATTGAGAATCGGCCCTGCAGTAAA<br>AAACTTTACTGCAGGGCCGATTCT	Cloning of *NtCOL2a*$_{\text{PS1}}$ knockout construct
PS *NtCOL2b*$_{\text{exI 95..117 bp}}$ for<br>PS *NtCOL2b*$_{\text{exI 95..117 bp}}$ rev	ATTGGTCACAGCCCGCACACAAAT<br>AAACATTTGTGTGCGGGCTGTGAC	Cloning of *NtCOL2b*$_{\text{PS1}}$ knockout construct
PS *NtCOL2a/b*$_{\text{exI 269..291 bp}}$ for<br>PS *NtCOL2a/b*$_{\text{exI 269..291bp}}$ rev	ATTGACGGTGGTGACGGCGTGCCA<br>AAACTGGCACGCCGTCACCACCGT	Cloning of *NtCOL2a/b*$_{\text{PS1}}$ knockout construct
**Primers for isolation of genomic sequences**		
NtCOL2a 5′UTR for<br>*NtCOL2a* 3′UTR rev	CATTCCACGTGTCGCCTATG<br>ATCTCTGGTTAAGGACGCGG	Isolation of genomic *NtCOL2a* and *NtomCOL2*
NtCOL2b 5′UTR for<br>*NtCOL2b* 3′UTR rev	ATTAACTTCATTCCACGTGC<br>AGACAAATATCTCATCTATTCCATC	Isolation of genomic *NtCOL2b* and *NsCOL2*
**Primers for isolation of coding sequences**		
NtCOL2a 5’UTR for2<br>*NtCOL2a* 3’UTR rev2	AAAAGAAGCCAAAATTAGTTCA<br>TCTTGTTGGGTCAATCCC	Isolation of *NtCOL2a* coding sequence
NtCOL2a 5′UTR for2<br>*NtCOL2a/b* 3′UTR rev	AAAAGAAGCCAAAATTAGTTCA<br>TTGATCTTCTCTTTGCTGC	Isolation of *NtomCOL2* coding sequence
NtCOL2b 5′UTR for2<br>*NtCOL2a/b* 3′UTR rev	AAAATAAGCCAAAAATTAGTTCA<br>TTGATCTTCTCTTTGCTGC	Isolation of *NtCOL2b* and *NsCOL2* coding sequence
**Primers for analysis of transgene integration and genotyping**		
NtGAPDH for<br>*NtGAPDH* rev	TGGAAGAATTGGGCGATTAGTG<br>GAGCAGCAGCCTTGTCCTTG	Genomic PCR *NtGAPDH* (control) (Schmidt et al., 2020)
Cas9 for<br>*Cas9* rev	ACGTGACCGAGGGAATGAGG<br>TTGCAGGAGATCCAGCGAGG	Identification of genomic *Cas9* integration (Schmidt et al., 2020)
P_{35S} diagn for<br>*NtCOL2a* diagn rev	TTGATGTGATATCTCCACTG<br>TAGGGTACCTGGAATGGGC	Genomic identification of P_{35S}:*NtCOL2a* and P_{Q35S}:*NtCOL2a-3xc-myc* integration

Table S2: List of primers and oligonucleotides used in this study. Continued.

**Name**	**Sequence (5′ to 3′)**	**Usage (Reference)**
P_{35S} diagn for<br>*NtCOL2b* diagn rev	TTGATGTGATATCTCCACTG<br>AGGGTACCTGGAATAGGG	Genomic identification of P_{35S}:*NtCOL2b* and P_{Q35S}:*NtCOL2b-3xc-myc* integration
NtCOL2a 5′UTR for<br>*uidA* screen rev	CATTCCACGTGTCGCCTATG<br>TCTGCATCGGCGAACTGATCG	Genomic identification of $P_{NtCOL2a}$:*uidA* integration (Schmidt et al., 2020)
NtCOL2a 5′UTR for<br>*GFP_{ER}* screen rev	CATTCCACGTGTCGCCTATG<br>AAGTAGTGACAAGTGTTGG	Genomic identification of $P_{NtCOL2a}$:*GFP_{ER}* integration
$P_{NtCOL2b}$ diagn for<br>*uidA* screen rev	TCTCTTGGACAAAGCTCTCC<br>TCTGCATCGGCGAACTGATCG	Genomic identification of $P_{NtCOL2b}$:*uidA* integration (Schmidt et al., 2020)
$P_{NtCOL2b}$ diagn for<br>*GFP_{ER}* screen rev	TCTCTTGGACAAAGCTCTCC<br>AAGTAGTGACAAGTGTTGG	Genomic identification of $P_{NtCOL2b}$:*GFP_{ER}* integration
NtCOL2a 5′UTR for<br>*NtCOL2a* intr1 rev	CATTCCACGTGTCGCCTATG<br>TAGGGTACCTGCATGAAAATATTC	Amplification of *NtCOL2a* (exon I) for sequencing
NtCOL2b 5′UTR for<br>*NtCOL2b* intr1 rev	ATTAACTTCATTCCACGTGC<br>GGGTACCTGCATAACATATTTAGG	Amplification of *NtCOL2b* (exon I) for sequencing
**Primers for qPCR expression analysis**		
qRT *Nt*/*NtomEF-1α* for<br>qRT *Nt*/*NtomEF-1α* rev	AAGCTGACTGTGCTGTCCTGA<br>GGTGGTAGCATCCATCTTGTTG	Expression analysis of *NtEF-1α* and *NtomEF-1α* (Beinecke et al., 2018)
qRT *NsEF-1α* for<br>qRT *NsEF-1α* rev	AAGCTGACTGTGCTGTCCTGA<br>GGTGGTAGCATCCATCTTGTTA	Expression analysis of *NsEF-1α* (Beinecke et al., 2018)
qRT *NtCOL2a*/*NtomCOL2* for<br>qRT *NtCOL2a*/*NtomCOL2* rev	TGCCCATTCCAGGTACCCTA<br>AGAACCCATCATCCTCCGTG	Expression analysis of *NtCOL2a* and *NtomCOL2*
qRT *NtCOL2b/NsCOL2* for<br>qRT *NtCOL2b/NsCOL2* rev	TCCCTATTCCAGGTACCCTG<br>CAAGAACCTATCATCCTCCGTT	Expression analysis of *NtCOL2b* and *NsCO2*
qRT *NtFT1* for<br>qRT *NtFT1* rev	AAGCAACCCAAACCTGAGGGAGTATCTG<br>GCAGCAACAGGCGAATTGAGATTATGAAATCTC	Expression analysis of *NtFT1* (Beinecke et al., 2018)
qRT *NtFT2/NsFTa* for<br>qRT *NtFT2/NsFTa* rev	AGATATCCCTGCAACCACAGAAGCAAC<br>AAACAGCGGCAACAGGCAAATTGAGAC	Expression analysis of *NtFT2/NsFTa* (Beinecke et al., 2018)
qRT *NsFTb* for<br>qRT *NsFTb* rev	CAACCACAGGAGCACACTTC<br>AGTACATAGCAGCAACAGGCG	Expression analysis of *NsFTb* (Beinecke et al., 2018)
qRT *NtFT4* for<br>qRT *NtFT4* rev	GATATCCCAGCAACTACAGATACAAG<br>GAAACGGGCAAACCAAGATTGTAAAC	Expression analysis of *NtFT4* (Beinecke et al., 2018)
qRT *NtFT5* for<br>qRT *NtFT5* rev	AGTTGGTGGAGATGACCTTCGTAC<br>CCATCCTGGAGCATACACTGTCTG	Expression analysis of *NtFT5* (Beinecke et al., 2018)
qRT *NsFTc* for<br>qRT *NsFTc* rev	GATATCCCAGCAACTACAGATACAAG<br>CAACAGGCAAACCAAGATTGTAAAG	Expression analysis of *NsFTc* (Beinecke et al., 2018)
qRT *NsFTd* for<br>qRT *NsFTd* rev	TCCCAAGTTGTTAACCAGCCA<br>CTTTCATAGCACACAATTTCTTGC	Expression analysis of *NsFTd* (Beinecke et al., 2018)

Vectors and cloning strategies

The plasmid vectors used in this study are listed in Supplementary Table S3.

Supplementary Table S3: Plasmid vectors used in this study. Resistance phenotypes: ampicillin (Amp^R), hygromycin (Hyg^R), kanamycin (Kan^R), phosphinothricin (PPT^R), spectinomycin (Sp^R), streptomycin (Str^R).

Name	Resistance	Reference
pBatTL-Venus-*ccdB*	Sp^R, Str^R (bacterial)	Dr. Joachim Uhrig, Dr. Guido Jach (Cologne, Germany); modified by Müller et al. (2010)
pBin19	Kan^R (bacterial and plant)	(Bevan, 1984)
pBin19 Q35S	Kan^R (bacterial and plant)	(Bevan, 1984), modified by Andrea S. Caesar (Münster, Germany)
pBin19Hyg	Kan^R (bacterial), Hyg^R (plant)	(Bevan, 1984), modified by Dr. Lena Grundmann (Münster, Germany
pCR II TOPO	Amp^R, Kan^R (bacterial)	Thermo Fisher Scientific
pENTR4	Kan^R (bacterial)	Thermo Fisher Scientific
pBsGFP$_{ER}$	Kan^R (bacterial)	(Noll et al., 2007)
pBsGUS	Kan^R (bacterial)	(Schmidt et al., 2020)
pDe-Cas9	Sp^R (bacterial), PPT^R (plant)	(Fauser et al., 2014)
pEn-Chimera	Amp^R (bacterial)	(Fauser et al., 2014)
pJET1.2/blunt	Amp^R (bacterial)	Thermo Fisher Scientific
pLab12.10 pDe-Cas9	Sp^R (bacterial), Kan^R(plant)	(Fauser et al., 2014), modified by Annika Wieghaus (Münster, Germany)
pRT104	Amp^R (bacterial)	(Töpfer et al., 1987)

Cloning of constructs for overexpression

The stable overexpression of *NtCOL2a* and *NtCOL2b* in *N. tabacum* was achieved using the overexpression constructs P_{35S}:*NtCOL2a* and P_{35S}:*NtCOL2b* (Supplementary Table S4). The coding sequences of *NtCOL2a* and *NtCOL2b* were amplified by PCR (Phusion High-Fidelity DNA Polymerase, Thermo Fisher Scientific) with the oligonucleotides listed in Supplementary Table S4, including 5' restriction sites. The coding cDNA of each target gene served as PCR template. Following preparative restriction of the insert and vector pRT104 (Töpfer et al., 1987), with XhoI/XbaI (New England Biolabs), the target genes were inserted by ligation using T4 DNA ligase (Promega) between the cauliflower mosaic virus (CaMV) 35S promoter (P_{35S}) and 35S terminator (T_{35S}). For the integration of overexpression cassettes P_{35S}:*NtCOL2a*/T_{35S} and P_{35S}:*NtCOL2b*/T_{35S} into the binary vector pBin19 Hyg, which contained *hpt* instead of *nptII* as the selectable marker gene (Bevan, 1984, modified by Dr. Lena Grundmann, Münster Germany), the pRT104 constructs were digested with HindIII and inserted into the target vector cut with the same enzyme.

Supplementary Table S4: Cloning strategies for overexpression constructs. The indicated restriction enzymes were used to digest PCR amplicons and vectors. For primer sequence data, see Supplementary Table S2.

Entry vector	Construct	Primer	Destination vector
pRT104 (XhoI/XbaI)	P_{35S}:*NtCOL2a*	*NtCOL2a/b* for XhoI<br>*NtCOL2a* rev XbaI	pBin19Hyg (HindIII)
	P_{35S}:*NtCOL2b*	*NtCOL2a/b* for XhoI<br>*NtCOL2b* rev XbaI	

Cloning of constructs for promoter–reporter assays

The sequences of $P_{NtCOL2a}$ and $P_{NtCOL2b}$ (~2.5kb) were fused to reporter genes *uidA* and *GFP$_{ER}$* encoding β-glucuronidase (GUS) and endoplasmic reticulum-localized green fluorescent protein (GFP$_{ER}$), respectively. The promoters were amplified from *N. tabacum* genomic DNA by PCR using primers containing 5′ overhangs with restriction sites to generate the constructs listed in Supplementary Table S5. Amplicons were digested with appropriate enzymes and inserted by ligation using T4 DNA ligase

into appropriately digested (Supplementary Table S5) entry vectors pBsGFP$_{ER}$ (Noll et al., 2007) and pBsGUS (Schmidt et al., 2020) upstream of the reporter genes. For plant transformation, the promoter–reporter cassettes P$_{NtCOL2a}$:*uidA*/ T$_{35S}$, P$_{NtCOL2b}$:*uidA*/ T$_{35S}$, P$_{NtCOL2a}$:*GFP$_{ER}$*/ T$_{35S}$ and P$_{NtCOL2b}$:*GFP$_{ER}$*/ T$_{35S}$ were each transferred by digestion with appropriate enzymes and ligation to appropriately digested binary destination vector pBin19 (Bevan, 1984) (Supplementary Table S5).

Supplementary Table S5: Cloning strategies for promoter–reporter constructs. The indicated restriction enzymes were used to digest PCR amplicons and vectors. For primer sequence data, see Supplementary Table S2.

Entry vector	Construct	Primer	Destination vector
pBsGFP$_{ER}$ (KpnI/XhoI)	P$_{NtCOL2a}$:*GFP$_{ER}$*	P$_{NtCOL2a}$ for KpnI<br>P$_{NtCOL2a}$ rev SalI	pBin19 (KpnI/BamHI)
	P$_{NtCOL2b}$:*GFP$_{ER}$*	P$_{NtCOL2b}$ for KpnI<br>P$_{NtCOL2b}$ rev XhoI	
pBsGUS (KpnI/XhoI)	P$_{NtCOL2a}$:*uidA*	P$_{NtCOL2a}$ for KpnI<br>P$_{NtCOL2a}$ rev SalI	pBin19 (KpnI/BamHI)
	P$_{NtCOL2b}$:*uidA*	P$_{NtCOL2b}$ for KpnI<br>P$_{NtCOL2b}$ rev XhoI	

Cloning of constructs for the immunodetection of fusion proteins

For the immunodetection of NtCOL2a and NtCOL2b, the proteins were C-terminally fused with three repeats of the human c-myc proto-oncogene epitope tag (3xc-myc) (Evan et al., 1985). The coding sequences of *NtCOL2a* and *NtCOL2b* were amplified by PCR using primers containing the indicated restrictions sites to generate the constructs listed in Supplementary Table S6. For the C-terminal fusion of the proteins with the tag, the reverse primers lacked the stop codon of *NtCOL2a* or *NtCOL2b* and included 5′ overhangs, which encoded a linker peptide of 10 amino acids (containing glycine and serine) followed by the 3xc-myc epitope tag and a stop codon. The tag in triplicate contained a synthetic peptide corresponding to the C-terminal amino acids of the human c-myc protein (positions 410-419, Evan et al., 1985). The XhoI/XbaI-digested PCR fragments were inserted by ligation into the appropriately digested binary destination vector pBin19 Q35S (Bevan, 1984; modified by Andrea S. Caesar, Münster, Germany) containing an expression cassette including a version of the CaMV 35S promoter (designated P$_{Q35S}$), the tobacco mosaic virus (TMV) Ω 5′-translational leader, and the CaMV 35S terminator (T$_{35S}$) from vector pFGCGW (Zhang and Galbraith, 2012). The PCR fragments were inserted downstream of the P$_{Q35S}$ and TMV Ω 5′-leader for the constitutive overexpression of *NtCOL2a -3xc-myc* and *NtCOL2b-3xc-myc*.

Supplementary Table S6: Cloning strategies for immunodetection constructs. The indicated restriction enzymes were used to digest PCR amplicons and the destination vector. For primer sequence data, see Supplementary Table S2.

Destination vector	Construct	Primer
pBin19 Q35S (XhoI/XbaI)	P$_{Q35S}$:*NtCOL2a-3xc-myc*	*NtCOL2a/b* for XhoI<br>*NtCOL2a 3xc-myc* rev XbaI
	P$_{Q35S}$:*NtCOL2b-3xc-myc*	*NtCOL2a/b* for XhoI<br>*NtCOL2b 3xc-myc* rev XbaI

Cloning of constructs for subcellular localization

For the analysis of subcellular protein localization in *N. benthamiana* leaf epidermal cells, NtCOL2a and NtCOL2b were N-terminally fused to YFP variant Venus (Nagai et al., 2002) and transiently expressed under the control of CaMV P$_{35S}$. The *NtCOL2a* and *NtCOL2b* coding sequence were amplified by PCR using primers containing the restriction sites indicated in Supplementary Table S7. They were then digested with appropriate enzymes and ligated into pENTR4 (Thermo Fisher Scientific) between the attachment sites. *NtCOL2a* and *NtCOL2b* were transferred to destination vector pBatTL-*Venus-ccdB* (Müller et al., 2010) by recombinational cloning using Gateway LR Clonase II

Enzyme Mix (Thermo Fisher Scientific) and were inserted downstream of the *Venus* gene to generate the P_{35S}:*Venus-NtCOL2a* and P_{35S}:*Venus-NtCOL2b* constructs. The original pBatTL vector was provided by Dr. Joachim Uhrig and Dr. Guido Jach (Cologne, Germany).

Supplementary Table S7: Cloning strategy for subcellular localization constructs. The indicated restriction enzymes were used to digest PCR amplicons and the entry vector. For primer sequence data, see Supplementary Table S2.

Entry vector	Construct	Primer	Destination vector
pENTR4 (SalI/XbaI)	P_{35S}:*Venus-NtCOL2a*	*NtCOL2a/b* for XhoI<br>*NtCOL2a* rev XbaI	pBatTL-*Venus-ccdB*
pENTR4 (SalI/XbaI)	P_{35S}:*Venus-NtCOL2b*	*NtCOL2b* for XhoI<br>*NtCOL2b* rev XbaI	pBatTL-*Venus-ccdB*

Cloning of constructs for gene knockouts

The constructs listed in Supplementary Table S8 were generated for the targeted editing of *NtCOL2a* and *NtCOL2b* using the CRISPR/Cas9 system. Based on the genome sequence of *N. tabacum* cv. Basma Xanthi (Sierro et al., 2014), we used CCTop (Stemmer et al., 2015; https://cctop.cos.uni-heidelberg.de) to find gene-specific protospacers (PSs), each targeting a site in exon I or exon II of the corresponding gene. For the simultaneous knockout of *NtCOL2a* and *NtCOL2b*, one protospacer (designated *NtCOL2a*$_{PS1}$) targeted both genes. Potential protospacer off-targets were predicted by screening the *N. tabacum* genome with CCTop for related sites with NGG-type and NRG-type protospacer adjacent motifs (PAMs). Only sequences containing a maximum of two mismatches in the 12-bp core region of the protospacer and four mismatches in total were considered as potential off-targets (Stemmer et al. (2015) and references therein). Protospacers were generated by annealing (5 min at 95 °C, followed by cooling to room temperature for 20 min) two single-stranded oligonucleotides (Supplementary Table S8), which included 5′ overhangs compatible with BbsI-linearized entry vector pEn-Chimera (Fauser et al., 2014). The cassettes containing the gene-specific sgRNAs were each transferred to destination vector pLab12.10 pDe-Cas9 (Fauser et al., 2014; modified by Annika Wieghaus, Münster, Germany) by recombinational cloning as described above. The destination vector was based on pDe-Cas9 (Fauser et al., 2014) with the native resistance cassette being replaced with the kanamycin resistance cassette from vector pLab12.10 (Xing et al., 2014), which consisted of a mannopine synthase promoter, a kanamycin resistance gene (*nptII*), and mannopine synthase polyA signal. The original vectors pEn-Chimera and pDe-Cas9 were kindly provided by Dr. Holger Puchta (Karlsruhe, Germany).

Supplementary Table S8: Cloning strategy for CRISPR/Cas9 knockout constructs. The indicated restriction enzyme was used for linearization of the entry vector. The numbers shown in the oligonucleotide names correspond to the protospacer (PS) location in the coding region of the target gene(s) (in base pairs, bp). For oligonucleotide sequence data, see Supplementary Table S2.

Entry vector	Construct	Oligonucleotide	Destination vector
pEN-Chimera (BbsI)	*NtCOL2a*$_{PS1}$	PS *NtCOL2a*$_{exI\ 71..93\ bp}$ for<br>PS *NtCOL2a*$_{exI\ 71..93\ bp}$ rev	pLab12.10 pDe-Cas9
	NtCOL2b$_{PS1}$	PS *NtCOL2b*$_{exI\ 95..117\ bp}$ for<br>PS *NtCOL2b*$_{exI\ 95..117\ bp}$ rev	
	NtCOL2a/b$_{PS1}$	PS *NtCOL2a/b*$_{exI\ 269..291\ bp}$ for<br>PS *NtCOL2a/b*$_{exI\ 269..291bp}$ rev	

Accession numbers

The accession numbers of gene and protein sequences used in this study are listed in Supplementary Table S9–S11.

Supplementary Table S9: Accession numbers of proteins in the initial BLAST analysis, where 17 Arabidopsis CO(L)/BBX proteins were used as queries against tobacco sequences in the NCBI nr database. The analysis identified 27 protein sequences in *N. tabacum*, some of which were newly assigned in this study (green) or previously described Zhao et al. 2022 (yellow). The transcripts of some of the newly assigned proteins were already known and described by Song et al. 2022 (blue). For the assignment of already known sequences identified in this study see Supplementary Table S10.

**Protein query sequences (*A. thaliana*, from Zhao et al., 2022)**			**Protein sequences identified in *N. tabacum* (Zhao et al., 2022 ; this study)**			
**BBX-name**	**CO(L)-name**	**Accession number, data library TAIR**	**COL-name** (used in this study)	**Accession number, data library NCBI (nr)**	**COL name** (Zhao et al., 2022)	**BBX-name** (for details see Table S10)
AtBBX1<br>AtBBX2<br>AtBBX3	AtCO<br>AtCOL1<br>AtCOL2	At5g15840<br>At5g15850<br>At3g02380	NtCOL2a<br>NtCOL2b	NP_001311813.1<br>XP_016462705.1	NtCOL2<br>-	NtBBX1<br>NtBBX2
AtBBX4	AtCOL3	At2g24790		XP_016463845.1<br>XP_016478497.1	-<br>-	NtBBX4<br>NtBBX5
AtBBX5	AtCOL4	At5g24930		XP_016495106.1<br>XP_016509100.1	-<br>NtCOL4	NtBBX6<br>NtBBX7
AtBBX6	AtCOL5	At5g57660		XP_016435645.1	NtCOL5	NtBBX3
AtBBX14	AtCOL6	At1g68520		XP_016463604.1<br>XP_016507682.1	-<br>-	NtBBX14<br>NtBBX15
AtBBX16	AtCOL7	At1g73870		XP_016434941.1<br>XP_016478271.1	NtCOL16b<br>NtCOL6	NtBBX16<br>NtBBX17
AtBBX17	AtCOL8	At1g49130			-	
AtBBX7	AtCOL9	At3g07650		XP_016477957.1<br>XP_016497584.1	-<br>NtCOL9	NtBBX46<br>NtBBX45
AtBBX8	AtCOL10	At5g48250		XP_016466834.1<br>XP_016497773.1	-<br>NtCOL10	NtBBX48<br>NtBBX47
AtBBX9	AtCOL11	At4g15250		XP_016435216.1<br>XP_016516093.1	-<br>-	NtBBX49<br>NtBBX50
AtBBX10	AtCOL12	At3g21880		XP_016488408.1<br>XP_016461866.1	-<br>-	NtBBX52<br>NtBBX51
AtBBX11	AtBBX11	At2g47890		XP_016509923.1<br>XP_016495983.1	NtCOL13<br>-	NtBBX9<br>NtBBX8
AtBBX12	AtCOL14	At2g33500		XP_016501680.1<br>XP_016500065.1	NtCOL14<br>-	NtBBX44<br>NtBBX10
AtBBX13	AtCOL15	At1g28050		XP_016446102.1<br>XP_016460898.1	-<br>-	NtBBX11<br>NtBBX53
AtBBX15	AtCOL16	At1g25440		XP_016453930.1<br>XP_016494942.1	NtCOL16a<br>-	NtBBX12<br>NtBBX13

Supplementary Table S10: Assignment of the *N. tabacum* protein sequences identified in this study to the known *NtBBX* (Song et al., 2022) and NtCOL (Zhao et al., 2022) sequences. *NtBBX* sequences were assigned to NtBBX/NtCOLs by BlastX and newly assigned sequences in this study are highlighted green. # The evolutionary origin of NtCOLs/NtBBXs was assigned using BlastP analysis of the proteins of interest against the NCBI nr database selecting only *N. tomentosiformis* (Ntom) and *N. sylvestris* (Ns) sequences.

**phylogenetic group of BBX proteins** (Fig. S1 and Song et al., 2022)	***BBX* name** (Song et al., 2022 and this study)	**Transcript** Accession number, data library NCBI (nt)	**Protein** Accession number, data library NCBI (nr)	**COL-name** (used in this study)	**COL-name** (Zhao et al., 2022)	**Origin** #
I	*NtBBX1*	XM_016584401.1	NP_001311813.1	NtCOL2a	NtCOL2	Ntom
I	*NtBBX2*	XM_016607219.1	XP_016462705.1	NtCOL2b		Ns
I	*NtBBX3*	XM_016580159.1	XP_016435645.1		NtCOL5	Ns
I	*NtBBX4*	XM_016608359.1	XP_016463845.1			Ntom
I	*NtBBX5*	XM_016623011.1	XP_016478497.1			Ns
I	*NtBBX6*	XM_016639620.1	XP_016495106.1			Ntom
I	*NtBBX7*	XM_016653614.1	XP_016509100.1		NtCOL4	Ns
II	*NtBBX8*	XM_016640497.1	XP_016495983.1			Ns
II	*NtBBX9*	XM_016654437.1	XP_016509923.1		NtCOL13	Ntom
II	*NtBBX10*	XM_016644579.1	XP_016500065.1			Ns
II	*NtBBX11*	XM_016590616.1	XP_016446102.1			Ntom
III	*NtBBX12*	XM_016598444.1	XP_016453930.1		NtCOL16a	Ntom
III	*NtBBX13*	XM_016639456.1	XP_016494942.1			Ns
III	*NtBBX14*	XM_016608118.1	XP_016463604.1			Ntom
III	*NtBBX15*	XM_016652196.1	XP_016507682.1			Ns
III	*NtBBX16*	XM_016579455.1	XP_016434941.1		NtCOL16b	Ntom
III	*NtBBX17*	XM_016622785.1	XP_016478271.1		NtCOL6	Ns
IV	*NtBBX18*	XM_016604881.1	XP_016460367.1			Ntom
IV	*NtBBX19*	XM_016627591.1	XP_016483077.1			Ns
IV	*NtBBX20*	XM_016623094.1	XP_016478580.1			Ns
IV	*NtBBX21*	XM_016652435.1	XP_016507921.1			Ntom
IV	*NtBBX22*	XM_016615553.1	XP_016471039.1			Ntom
IV	*NtBBX23*	XM_016656120.1	XP_016511606.1			Ns
IV	*NtBBX24*	XM_016643247.1	XP_016498733.1			Ntom
IV	*NtBBX25*	XM_016645077.1	XP_016500563.1			Ns
(IV)	*NtBBX26*	XM_016620558.1	XP_016476044.1			Ns
(IV)	*NtBBX27*	XM_016629035.1	XP_016484521.1			Ntom
IV	*NtBBX28*	XM_016603147.1	XP_016458633.1			Ns
IV	*NtBBX29*	XM_016607710.1	XP_016463196.1			Ntom
IV	*NtBBX30*	XM_016643950.1	XP_016499436.1			Ns
IV	*NtBBX31*	XM_016658627.1	XP_016514113.1			Ntom
IV	*NtBBX32*	XM_016590865.1	XP_016446351.1			Ns
IV	*NtBBX33*	XM_016620324.1	XP_016475810.1			Ntom
IV	*NtBBX34*	XM_016631354.1	XP_016486840.1			Ntom
IV	*NtBBX35*	XM_016656924.1	XP_016512410.1			Ns
V	*NtBBX36*	XM_016580655.1	XP_016436141.1			Ntom
V	*NtBBX37*	XM_016618260.1	XP_016473746.1			Ns
V	*NtBBX38*	XM_016586479.1	XP_016441965.1			Ns
V	*NtBBX39*	XM_016660406.1	XP_016515892.1			Ntom
V	*NtBBX40*	XM_016582192.1	XP_016437678.1			Ns
V	*NtBBX41*	XM_016650470.1	XP_016505956.1			Ntom
V	*NtBBX42*	XM_016638592.1	XP_016494078.1			Ntom
V	*NtBBX43*	XM_016647357.1	XP_016502843.1			Ns
II	*NtBBX44*	XM_016646194.1	XP_016501680.1		NtCOL14	Ntom
II	*NtBBX45*	XM_016642098.1	XP_016497584.1		NtCOL9	Ns
II	*NtBBX46*	XM_016622471.1	XP_016477957.1			Ntom
II	*NtBBX47*	XM_016642287.1	XP_016497773.1		NtCOL10	Ns
II	*NtBBX48*	XM_016611348.1	XP_016466834.1			Ntom
II	*NtBBX49*	XM_016579730.1	XP_016435216.1			Ntom
II	*NtBBX50*	XM_016660607.1	XP_016516093.1			Ns
II	*NtBBX51*	XM_016606380.1	XP_016461866.1			Ns
II	*NtBBX52*	XM_016632922.1	XP_016488408.1			Ntom
II	*NtBBX53*	XM_016605412.1	XP_016460898.1			Ns

Supplementary Table S11: Accession numbers of genes and proteins identified, described and/or used in this study, which are not listed in Table S9 and Table S10.

Gene/ protein	Accession number(s), data library	Reference
NtCOL2a	OR764757 (coding sequence), Genbank<br>OR764753 (genomic sequence), GenBank	this study<br>this study
NtCOL2b	OR764759 (coding sequence), Genbank<br>OR764755 (genomic sequence), GenBank	this study<br>this study
NtomCOL2	OR764758 (coding sequence), Genbank<br>OR764754 (genomic sequence), GenBank	this study<br>this study
NsCOL2	OR764760 (coding sequence), Genbank<br>OR764756 (genomic sequence), GenBank	this study<br>this study
NtFT1^{rep}	JX679067 (coding sequence), GenBank<br>AYMY01080681.1 (genomic sequence), GenBank	(Harig et al., 2012)<br>(Sierro et al., 2014, assigned to *NtFT1*^{rep} in this study)
NtFT2^{rep}	JX679068 (coding sequence), GenBank<br>AWOK01464621.1 (genomic sequence), GenBank	(Harig et al., 2012)<br>(Sierro et al., 2014, assigned to *NtFT2*^{rep} in this study)
NtFT3^{rep}	JX679069 (coding sequence), GenBank<br>AYMY01125653.1 (genomic sequence), GenBank	(Harig et al., 2012)<br>(Sierro et al., 2014, assigned to *NtFT3*^{rep} in this study)
NtFT4^{act}	JX679070 (coding sequence), GenBank<br>genomic sequence	(Harig et al., 2012)<br>this study
NtFT5^{act}	KY306470 (coding sequence), GenBank<br>AYMY01148166.1 (genomic sequence), GenBank	(Beinecke et al., 2018)<br>(Sierro et al., 2014, assigned to *NtFT5*^{act} in this study)
NtFT6^{act}	KY306472.1 (coding sequence), GenBank	(Beinecke et al., 2018)
NtFT7^{act}	KY306474.1 (coding sequence), GenBank	(Beinecke et al., 2018)
NtomFTα^{rep}	KY306466.1 (coding sequence), GenBank<br>ASAG01015492.1 (genomic sequence), GenBank	(Beinecke et al., 2018)<br>(Sierro et al., 2013, assigned to *NtomFTα*^{rep} in this study)
NtomFTβ^{act}	KY306469.1 (coding sequence), GenBank<br>ASAG01071062.1 (genomic sequence), GenBank	(Beinecke et al., 2018)<br>(Sierro et al., 2013, assigned to *NtomFTβ*^{act} in this study)
NtomFTγ^{act}	KY306471.1 (coding sequence), GenBank<br>ASAG01034513.1 (genomic sequence), GenBank	(Beinecke et al., 2018)<br>(Sierro et al., 2013, assigned to *NtomFTγ*^{act} in this study)
NsFTa^{rep}	KY306467.1 (coding sequence), GenBank<br>ASAF01051554.1 (genomic sequence), GenBank	(Beinecke et al., 2018)<br>(Sierro et al., 2013, assigned to *NsFTa*^{rep} in this study)
NsFTb^{rep}	KY306468.1 (coding sequence), GenBank<br>ASAF01185429.1 (genomic sequence), GenBank	(Beinecke et al., 2018)<br>(Sierro et al., 2013, assigned to *NsFTb*^{rep} in this study)
NsFTc^{act}	KY306473.1 (coding sequence), GenBank<br>ASAF01108824.1/01287909.1 (genomic sequence), GenBank	(Beinecke et al., 2018)<br>(Sierro et al., 2013, assigned to *NsFTc*^{act} in this study)
NsFTd^{act}	KY306475.1 (coding sequence), GenBank<br>ASAF01036279.1 (genomic sequence), GenBank	(Beinecke et al., 2018)<br>(Sierro et al., 2013, assigned to *NsFTd*^{act} in this study)
NtomCOL2	XP_009630583.1 (protein sequence), GenBank	identified as NtomCOL2 in this study
NsCOL2	XP_009765376.1 (protein sequence), GenBank	identified as NsCOL2 in this study
SlCO1	NP_001233839.1 (protein sequence), GenBank	(Ben-Naim et al., 2006)
SlCO3	NP_001316711.1 (protein sequence), GenBank	(Ben-Naim et al., 2006)
StCO	NP_001274795.1 (protein sequence), GenBank	(González-Schain et al., 2012)
StCOL1	PGSC0003DMP400017796 (protein sequence), Plant TFDB	(Abelenda et al., 2016)
StCOL2	PGSC0003DMP400017799 (protein sequence), Plant TFDB	(Abelenda et al., 2016)
OsHd1	BAB17628.1 (protein sequence), GenBank	(Yano et al., 2000)
AtBBX18	At2g21320, TAIR	(Zhao et al., 2022)
AtBBX19	At4g38960, TAIR	(Zhao et al., 2022)
AtBBX20	At4g39070, TAIR	(Zhao et al., 2022)
AtBBX21	At1g75540, TAIR	(Zhao et al., 2022)
AtBBX22	At1g78600, TAIR	(Zhao et al., 2022)
AtBBX23	At4g10240, TAIR	(Zhao et al., 2022)
AtBBX24	At1g06040, TAIR	(Zhao et al., 2022)
AtBBX25	At2g31380, TAIR	(Zhao et al., 2022)
AtBBX26	At1g60250, TAIR	(Zhao et al., 2022)
AtBBX27	At1g68190, TAIR	(Zhao et al., 2022)
AtBBX28	At4g27310, TAIR	(Zhao et al., 2022)
AtBBX29	At5g54470, TAIR	(Zhao et al., 2022)
AtBBX30	At4g15248, TAIR	(Zhao et al., 2022)
AtBBX31	At3g21890, TAIR	(Zhao et al., 2022)
AtBBX32	At3g21150, TAIR	(Zhao et al., 2022)

Quantitative real-time PCR (qPCR)

Primer combinations used for expression analysis by qPCR in this study are listed in Supplementary Table S12.

Supplementary Table S12: Primers used for expression analysis by quantitative real-time PCR (qPCR). Annealing temperatures (T) are given in °C. For oligonucleotide sequence data, see Supplementary Table S2.

Target gene	Primer name	T [°C]
NtEF-1α / *NtomEF-1α*	qRT *Nt*/*NtomEF-1α* for<br>qRT *Nt*/*NtomEF-1α* rev	66.7
NsEF-1α	qRT *NsEF-1α* for<br>qRT *NsEF-1α* rev	66.2
NtCOL2a / *NtomCOL2*	qRT *NtCOL2a*/*NtomCOL2* for<br>qRT *NtCOL2a*/*NtomCOL2* rev	65
NtCOL2b / *NsCOL2*	qRT *NtCOL2b*/*NsCOL2* for<br>qRT *NtCOL2b*/*NsCOL2* rev	65
NtFT1	qRT *NtFT1* for<br>qRT *NtFT1* rev	69.2
NtFT2 / NsFTa	qRT *NtFT2/NsFTa* for<br>qRT *NtFT2/NsFTa* rev	70
NsFTb	qRT *NsFTb* for<br>qRT *NsFTb* rev	66.5
NtFT4	qRT *NtFT4* for<br>qRT *NtFT4* rev	67
NtFT5	qRT *NtFT5* for<br>qRT *NtFT5* for	64.7
NsFTc	qRT *NsFTc* for<br>qRT *NsFTc* rev	66.7
NsFTd	qRT *NsFTd* for<br>qRT *NsFTd* rev	67

Computational investigations of *Nicotiana FT* promoter regions

The results of computational analysis of *Nicotiana FT* promoter regions for the presence of CO-specific regulatory elements (COREs) are shown in Supplementary Table S13.

Supplementary Table S13: CO-specific regulatory elements (COREs) in *Nicotiana FT* promoter regions. We checked 5 kb of upstream promoter sequence (for accession numbers see Supplemental Table S11) using PLANTPAN3.0 (Chow et al., 2019; http://plantpan.itps.ncku.edu.tw) for the COREs TGTG($N_{2–3}$)ATG (Tiwari et al., 2010) and TGTGGT (Abelenda et al., 2016). (+) indicates sense strand; (-) indicates reverse strand

Target FT promoter	TGTGNNATG	TGTGNNNATG	TGTGGT
NtFT1^{rep} / NtomFTα^{rep}	4225 (-) / 4271 (-)	9 (-) / 9 (-)<br>1912 (-) / 1958 (-)<br>- / 3131 (+)	1208 (+) / 1284 (+)<br>1264 (+) / 1340 (+)<br>2993 (+) / 3039 (+)<br>3033 (+) / 3079 (+)
NtFT2^{rep} / NsFTa^{rep}		1225 (-) / 1224 (-)<br>1328 (-) / 1327 (-)<br>2221 (-) / 2124 (-)<br>2323 (-) / 2226 (-)	1266 (-) / 1265 (-)<br>2890 (+) / 2793 (+)<br>2962 (+) / 2865 (+)
NtFT3^{rep} / NsFTb^{rep}			1321 (+) / 1321 (+)<br>1377 (+) / 1377 (+)<br>4886 (-) / 4887 (-)
NtFT4^{act} / NtomFTβ^{act}<br>(1kb only)	- / 4102 (-)		134 (-) / 134 (-)<br>- / 1666 (+)<br>- / 1889 (-)
NtFT5^{act} / NtomFTγ^{act}	3428 (+) / 3414 (+)	1580 (-) / 1566 (-)	559 (-) / 545 (-)

References

Abelenda, J. A., Cruz-Oró, E., Franco-Zorrilla, J. M., and Prat, S. (2016). Potato StCONSTANS-like1 suppresses storage organ formation by directly activating the FT-like StSP5G repressor. Current Biology 26, 872–881. doi: 10.1016/j.cub.2016.01.066

Beinecke, F. A., Grundmann, L., Wiedmann, D. R., Schmidt, F. J., Caesar, A. S., Zimmermann, M., et al. (2018). The FT/FD-dependent initiation of flowering under long-day conditions in the day-neutral species Nicotiana tabacum originates from the facultative short-day ancestor Nicotiana tomentosiformis. The Plant Journal 96, 329–342. doi: 10.1111/tpj.14033

Bevan, M. (1984). Binary Agrobacterium vectors for plant transformation. Nucleic Acids Res 12, 8711–8721. doi: 10.1093/nar/12.22.8711

Chow, C.-N., Lee, T.-Y., Hung, Y.-C., Li, G.-Z., Tseng, K.-C., Liu, Y.-H., et al. (2019). PlantPAN3.0: a new and updated resource for reconstructing transcriptional regulatory networks from ChIP-seq experiments in plants. Nucleic Acids Res 47, D1155-D1163. doi: 10.1093/nar/gky1081

Crocco, C. D., and Botto, J. F. (2013). BBX proteins in green plants: Insights into their evolution, structure, feature and functional diversification. Gene 531, 44–52. doi: 10.1016/j.gene.2013.08.037

Evan, G. I., Lewis, G. K., Ramsay, G., and Bishop, J. M. (1985). Isolation of monoclonal antibodies specific for human c-myc proto-oncogene product. Molecular and Cellular Biology 5, 3610–3616. doi: 10.1128/mcb.5.12.3610

Fauser, F., Schiml, S., and Puchta, H. (2014). Both CRISPR/Cas-based nucleases and nickases can be used efficiently for genome engineering in Arabidopsis thaliana. The Plant Journal 79, 348–359. doi: 10.1111/tpj.12554

Harig, L., Beinecke, F. A., Oltmanns, J., Muth, J., Müller, O., Rüping, B., et al. (2012). Proteins from the FLOWERING LOCUS T-like subclade of the PEBP family act antagonistically to regulate floral initiation in tobacco. The Plant Journal 72, 908–921. doi: 10.1111/j.1365-313X.2012.05125.x

Jones, P., Binns, D., Chang, H.-Y., Fraser, M., Li, W., McAnulla, C., et al. (2014). InterProScan 5: genome-scale protein function classification. Bioinformatics 30, 1236–1240. doi: 10.1093/bioinformatics/btu031

Madeira, F., Park, Y. M., Lee, J., Buso, N., Gur, T., Madhusoodanan, N., et al. (2019). The EMBL-EBI search and sequence analysis tools. Nucleic Acids Res 47, W636—W641. doi: 10.1093/nar/gkz268

Madeira, F., Pearce, M., Tivey, A. R. N., Basutkar, P., Lee, J., Edbali, O., et al. (2022). Search and sequence analysis tools services from EMBL-EBI. Nucleic Acids Res 50, W276-W279. doi: 10.1093/nar/gkac240

Müller, B., Noll, G. A., Ernst, A. M., Rüping, B., Groscurth, S., Twyman, R. M., et al. (2010). Recombinant artificial forisomes provide ample quantities of smart biomaterials for use in technical devices. Applied Microbiology and Biotechnology 88, 689–698. doi: 10.1007/s00253-010-2771-4

Nagai, T., Ibata, K., Park, E. S., Kubota, M., Mikoshiba, K., and Miyawaki, A. (2002). A variant of yellow fluorescent protein with fast and efficient maturation for cell-biological applications. Nature Biotechnology 20, 87–90. doi: 10.1038/nbt0102-87

Noll, G. A., Fontanellaz, M. E., Rüping, B., Ashoub, A., van Bel, A. J. E., Fischer, R., et al. (2007). Spatial and temporal regulation of the forisome gene for1 in the phloem during plant development. Plant Molecular Biology 65, 285–294. doi: 10.1007/s11103-007-9217-0

Robson, F., Costa, M. M., Hepworth, S. R., Vizir, I., Piñeiro, M., Reeves, P. H., et al. (2001). Functional importance of conserved domains in the flowering-time gene CONSTANS demonstrated by analysis of mutant alleles and transgenic plants. The Plant Journal 28, 619–631. doi: 10.1046/j.1365-313x.2001.01163.x

Schmidt, F. J., Zimmermann, M. M., Wiedmann, D. R., Lichtenauer, S., Grundmann, L., Muth, J., et al. (2020). The major floral promoter NtFT5 in tobacco (Nicotiana tabacum) is a promising target for crop improvement. Frontiers in Plant Science 10, 1–16. doi: 10.3389/fpls.2019.01666

Sierro, N., Battey, J. N. D., Ouadi, S., Bakaher, N., Bovet, L., Willig, A., et al. (2014). The tobacco genome sequence and its comparison with those of tomato and potato. Nature Communications 5, 3833. doi: 10.1038/ncomms4833

Sierro, N., Battey, J. N. D., Ouadi, S., Bovet, L., Goepfert, S., Bakaher, N., et al. (2013). Reference genomes and transcriptomes of Nicotiana sylvestris and Nicotiana tomentosiformis. Genome Biology 14, R60-R60. doi: 10.1186/gb-2013-14-6-r60

Song, K., Li, B., Wu, H., Sha, Y., Qin, L., Chen, X., et al. (2022). The Function of BBX Gene Family under Multiple Stresses in Nicotiana tabacum. Genes 13. doi: 10.3390/genes13101841

Stemmer, M., Thumberger, T., Del Sol Keyer, M., Wittbrodt, J., Mateo, J. L., and Maas, S. (2015). CCTop: An intuitive, flexible and reliable CRISPR/Cas9 target prediction tool. PloS one 10, e0124633-e0124633. doi: 10.1371/journal.pone.0124633

Tamura, K., Stecher, G., and Kumar, S. (2021). MEGA 11: Molecular Evolutionary Genetics Analysis version 11. Molecular Biology and Evolution 38, 3022-3027. doi:10.1093/molbev/msab120

Tiwari, S. B., Shen, Y., Chang, H.-C., Hou, Y., Harris, A., Ma, S. F., et al. (2010). The flowering time regulator CONSTANS is recruited to the FLOWERING LOCUS T promoter via a unique cis-element. The New Phytologist 187, 57–66. doi: 10.1111/j.1469-8137.2010.03251.x

Töpfer, R., Matzeit, V., Gronenborn, B., Schell, J., and Steinbiss, H. H. (1987). A set of plant expression vectors for transcriptional and translational fusions. Nucleic Acids Res 15, 5890. doi: 10.1093/nar/15.14.5890

Xing, S., van Deenen, N., Magliano, P., Frahm, L., Forestier, E., Nawrath, C., et al. (2014). ATP citrate lyase activity is post-translationally regulated by sink strength and impacts the wax, cutin and rubber biosynthetic pathways. The Plant Journal 79, 270–284. doi: 10.1111/tpj.12559

Zhang, C., and Galbraith, D. W. (2012). RNA interference-mediated gene knockdown within specific cell types. Plant Molecular Biology 80, 169–176. doi: 10.1007/s11103-012-9937-7

Zhao, X., Yu, F., Guo, Q., Wang, Y., Zhang, Z., and Liu, Y. (2022). Genome-Wide Identification, Characterization, and Expression Profile Analysis of CONSTANS-like Genes in Woodland Strawberry (Fragaria vesca). Frontiers in Plant Science 13, 931721. doi.org/10.3389/fpls.2022.931721
